# Investigating Stability and Tautomerization of Gossypol—A Spectroscopy Study

**DOI:** 10.3390/molecules24071286

**Published:** 2019-04-02

**Authors:** Lulu Wang, Yanxia Liu, Yagang Zhang, Akram Yasin, Letao Zhang

**Affiliations:** 1 Xinjiang Technical Institute of Physics and Chemistry, Chinese Academy of Sciences, Urumqi 830011, China; wanglulu@ms.xjb.ac.cn (L.W.); liuyanxia@ms.xjb.ac.cn (Y.L.); akram@ms.xjb.ac.cn (A.Y.); zhanglt@ms.xjb.ac.cn (L.Z.); 2 University of Chinese Academy of Sciences, Beijing 100049, China; 3 Department of chemical and environmental engineering, Xinjiang Institute of Engineering, Urumqi 830026, China

**Keywords:** gossypol, tautomerization, ^1^H NMR, UV-vis, HPLC-QTOF-MS

## Abstract

The stability of gossypol was investigated by the spectroscopic method. Gossypol was dissolved in three different solvents (CHCl_3_, DMSO, and CH_3_OH) under different storage conditions (dark and with nitrogen protection, natural light and with nitrogen protection, ambient air conditions) for different time intervals (0 days, 3 days, 5 days, 7 days, 15 days, 30 days, and 45 days) at room temperature. Then, the stability of gossypol was investigated by ^1^H NMR, UV-vis, and HPLC-QTOF-MS spectrometry. Results showed that gossypol existed in aldehyde–aldehyde form in chloroform within five days. Then, both aldehyde–aldehyde and lactol–lactol tautomeric forms existed and maintained a stable solution for 45 days. Gossypol dissolved in methanol mainly existed in aldehyde–aldehyde form. Only a tiny amount of lactol–lactol was found in freshly prepared methanol solution. Gossypol was found to only exist in lactol–lactol form between 30–45 days. Gossypol existed in aldehyde–aldehyde, lactol–lactol, and ketol–ketol forms in dimethyl sulfoxide, and there was a competitive relationship between aldehyde–aldehyde and lactol–lactol form during the 45 days. Among all the solvents and conditions studied, gossypol was found to be highly stable in chloroform. Under the tested conditions, the natural light and atmospheric oxygen had little effect on its stability. Although the spectroscopy data seemed to be changed over time in the three different solvents, it was actually due to the tautomeric transformation rather than molecular decomposition.

## 1. Introduction

**Scheme 1:**
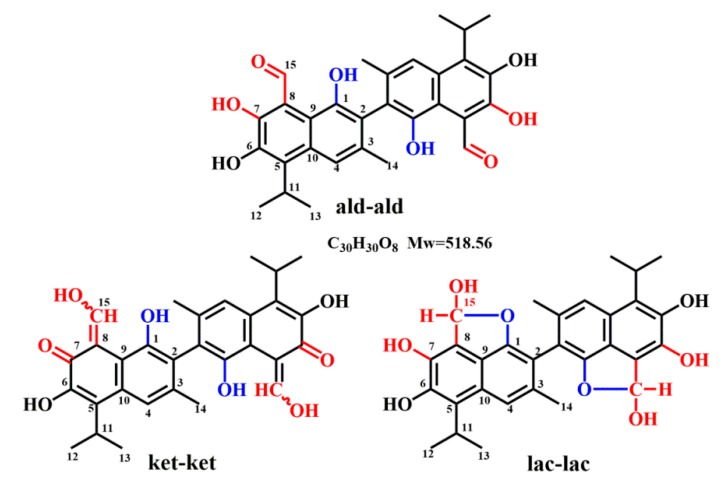
Structures of three tautomeric forms of gossypol.

Gossypol, a yellow polyphenolic aldehyde isolated from various Gossypium species, was discovered by Longmore and Marchlewski 100 years ago [1,2]. Adams et al. [3,4,5,6,7] were the pioneers who reported and elucidated the gossypol structure to be 1,1′,6,6′,7,7′-hexahydroxy-3,3′-dimethyl-5,5′-disopropyl-(2,2′-binaphthalene)-8,8′-dicarboxaldehyde. Gossypol was a dissymmetry molecule that has restricted rotation around the binaphthyl bond, and therefore, two enantiomers with different biological activity exist. Gossypol was found to exist in three tautomeric isomers, which were aldehyde–aldehyde, lactol–lactol, and ketol–ketol (Scheme 1). The early work has been focused on its physiological, chemical properties, and bioactivity [8,9,10,11,12,13,14]. Noticeably, gossypol has been studied and used as male anti-fertility drug in the late 1970s and early 1980s [15]. Methods were developed to detoxify the gossypol in cottonseeds meal and determine the total gossypol content [16]. It was proposed that gossypol was not stable in various solvents [17,18,19]. Jaroszewski et al. [20] proposed that according to the calculations and experimental data, gossypol with aldehyde form was not thermally racemizable, and gossypol with acetal form was thermally racemizable. Nomeir et al. [21,22] found that gossypol only accounted for 0–40% of the initial concentrations after being stored for 29 days by HPLC method. Marciniak et al. [23,24] showed that the UV-vis absorption spectra of gossypol changed drastically in solution over time. However, none of those studies reported to what structures gossypol had changed. Due to its unique structure and accessibility in large quantities, gossypol was a desirable drug lead compound.

The main obstacle in the application of gossypol in medical therapy was its high toxicity and complex structure. For this reason, studies were carried out to transform gossypol into diastereoisomeric Schiff bases to separate the racemic mixture. However, it was very difficult to obtain the enantiomers of gossypol [24,25]. Further studies on gossypol Schiff base indicated that the compound existed in solution as two tautomeric forms of enamine–enamine and imine–imine [26] However, it was difficult to evaluate the stability of gossypol without systematical and sufficient data. Studies using IR and NMR showed that gossypol mainly existed in aldehyde–aldehyde tautomeric form in CHCl_3_, CH_2_Cl_2_, and CH_3_CN, whereas it existed in in lactol–lactol and the aldehyde–aldehyde tautomer in DMSO. The ketol–ketol form was the main tautomeric form in alkaline solution. It seemed that solvents would influence the tautomeric transformation of gossypol. However, the influence and consequence of different storage conditions are not reported. 

Along these lines, in the work reported here, we focused on answering questions, including: is gossypol stable? What influences the stability of gossypol? If not stable, what will gossypol be under various conditions? We took a spectroscopy approach by combining the results of ^1^H NMR, HPLC-QTOF-MS, and UV-vis analysis, and systematically investigated the stability and tautomerization of gossypol in various solvents including chloroform, methanol, and dimethyl sulfoxide. To the best of our knowledge, our work was the first attempt at answering these important questions based on comprehensive spectroscopy investigation. The stability and tautomerization of gossypol in various solvents under different storage conditions over time were systematically studied, compared, and discussed. 

## 2. Results and Discussion

### 2.1. ^1^H NMR

The proton NMR was taken for samples stored under different storage conditions over time. The proton NMR spectrums of each gossypol sample are shown in Figure 1, Figure 2 and Figure 3. It was observed that three different storage conditions had little effect on the proton ^1^H NMR spectrum in terms of proton signals. These observations implied that the natural light and atmospheric oxygen on the stability of gossypol were negligible. The ^1^H NMR spectra of gossypol solution under three different storage condition over time in three different solvents were systematically compared (Supporting Information, Figures S1–S30). Therefore, the ambient air condition was chosen for the gossypol storage in the subsequent studies. 

Comparison of the spectra of gossypol in different solvents for on day 0 showed the characteristic pattern of the 15–CHO [27]. A singlet for the CHO proton was observed at 11.13 ppm in CDCl_3_ and at 11.32 ppm in CD_3_OD. The CHO proton was observed at 11.13 ppm and 11.16 ppm in *d*_6_. These results implied that in all three different fresh solvents, gossypol exists in aldehyde form [28]. For gossypol in *d*_6_, it was observed that 3.59-ppm peak intensity increased 3.74-ppm peak intensity decreased after five days, and there was a reciprocal relationship between them. The intensity decrease or increase was due to the conversion of different competition forms between aldehyde (3.74 ppm) and lactol (3.59 ppm). For gossypol in CD_3_OD, new peaks were observed at 6.81 ppm and 6.86 ppm, respectively. Results implied that the 15-CH (lactol) proton is asymmetrical in CD_3_OD [28].

According to the ^1^H NMR spectra, no chemical decomposition had taken place for gossypol stored in different solvents within five days. The signal changes were proposed to be the tautomeric transformation of gossypol. Additional experiments were carried out. The ^1^H NMR spectra of gossypol were obtained in three solvents (CHCl_3_, DMSO, and CH_3_OH) after 45 days.

The chemical shifts and assignments of the signals for gossypol in chloroform at different time intervals are summarized in Table 1. It was previously observed that gossypol in chloroform existed in aldehyde–aldehyde form and tiny quantities of lactol–lactol form within five days [27,28,29,30]. From day 7, the general pattern of the spectrum changed, and the signals from the lactol–lactol form were more obviously (Table 4). The signals are shifted downfield, and instead of one singlet, two closely spaced singlets were observed. For example, for a 7-OH proton, two separated signals at 15.19 ppm and 15.23 ppm showed up instead of a singlet at 15.20 ppm. Furthermore, signals at 11.12 and ppm 11.17 ppm, which belonged to the 15-CHO protons, were observed in different intensities. These observations could be due to the asymmetric localization nature of the gossypol 7-OH and 15-CHO group [31]. Furthermore, two new peaks appeared at 8.45 ppm and 7.33 ppm, of which the latter signal is in good agreement with the chemical shift of the hemiacetal proton (7.05 ppm) of the hexamethylether of (±) gossypol. This observation was interpreted as spectroscopic evidence of two tautomers, aldehyde and lactol, in CDCl_3_ (Table 1 and Table 4) [28]. 

The results showed that gossypol mainly existed in aldehyde tautomeric form in CDCl_3_. It was also found that small quantities of lactol–lactol forms started showing up after two days, and the ratio between the two forms did not change until 45 days (Supporting Information Figure S10). 

The chemical shifts of gossypol in methanol at different time intervals were shown in Table 2. Gossypol was found to have three major proton signal changes in methanol over time. The freshly made solution of gossypol in methanol was slightly different from the one in CDCl_3_ in the first stage. 

For gossypol in CD_3_OD, signals were observed at 7.01 ppm, 6.99 ppm, and 5.51 ppm within seven days, and then disappeared after 15 days. These signals were assigned to the H4, H4′ (Ph-H) and the 15-CH=OH of gossypol ketol–ketol form [23,32] which was different from the 15, 15′-dimethyl ethers of the dilactol form of gossypol [33]. 

The second signals change occurred from 8 h to 15 days. The proton signals of H4 and H4′(Ph-H) protons at 7.78 ppm and 7.69 ppm became signals at 7.42 ppm, 7.78 ppm, and 7.69 ppm from 8 h until three days. These peaks then became separated signals at 7.34 ppm, 7.43 ppm, 7.69 ppm, and 7.78 ppm from day 5 until day 15. These observed changes of chemical shifts were proposed due to the asymmetrical aldehyde–lactol forms [34]. The chemical shifts at 6.81 ppm and 6.77 ppm were assigned to the protons of hemiacetal of gossypol lactol–lactol form [33]. It should be noted that it is possible to observed pairs of enantiomers: (SAX,R,R) and (RAX,S,S), (SAX,R,R) and (SAX,S,S), and (SAX,S,R) and (RAX,S,R), which are diastereoisomers.

The chemical shifts of gossypol protons in dimethyl sulfoxide stored over time were summarized in Table 3. Noticeably, for gossypol in DMSO-*d*_6_ compared with in CDCl_3_ and CD_3_OD, it always existed in three tautomeric forms for the freshly made solution until 45 days. The spectrum demonstrated its most complex pattern. The protons of –CH_3_, (CH_3_)_2_CH–, O–H, –CHO, and the Ph–H group observed two, four, and multiple separated signals. 

The observed chemical shifts and intensity changes implied equilibrium among the symmetrical tautomeric forms of aldehyde, ketol, and lactol (including asymmetrical aldehyde–lactol forms) that occurred in solvents [28]. Whereas, the content of gossypol aldehyde forms at day 7 was inversely related to the content of gossypol in *d*_6_ at day 15. The signal intensity of the H14, H14’(Ph–CH_3_) (lac) single at 2.31 ppm and 15–OH–CH (lac) at 9.02 ppm and 9.10 ppm increased at day 7, but decreased at day 15. It was interesting noting that the singlets at 5.51 ppm and 5.57 ppm that were observed in CD_3_OD and DMSO-*d*_6_ completely vanished after 15 days. The 15-CH=OH proton of the ketol form at 9.50 ppm implied that gossypol in DMSO-*d*_6_ was in the ketol form, and the ketol was unstable under neutral or acidic conditions and converted to the aldehyde tautomer [35,36].

The comparison of the ^1^H NMR spectrum of gossypol in DMSO-*d*_6_ at different times was summarized (Supporting Information Figure S30), and the results were consistent with the UV-vis spectra.

**Figure 1:**
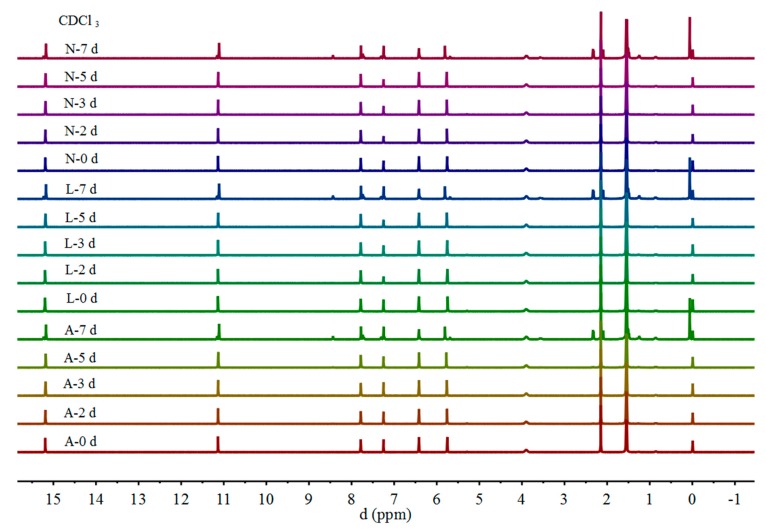
^1^H NMR spectrum of the gossypol in chloroform-d under different storage conditions over time (N: normal condition without protecting from air and light; L: under natural light and nitrogen protection; A: stored in dark and under nitrogen protection).

**Figure 2:**
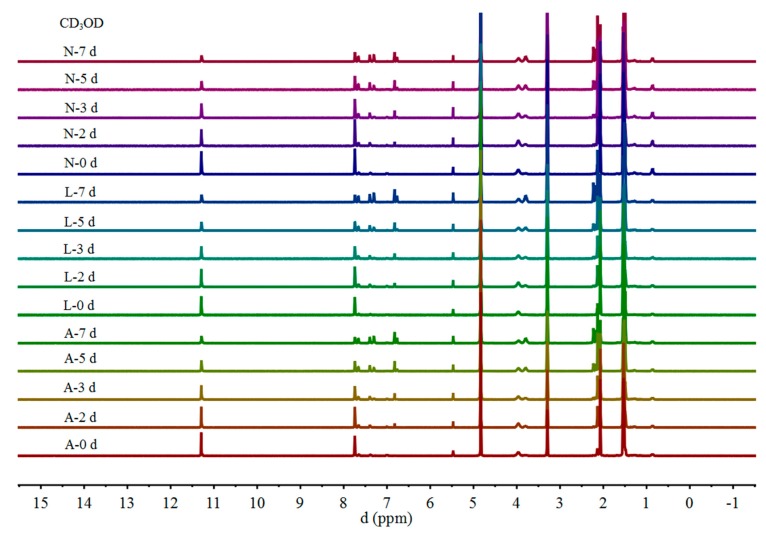
^1^H NMR spectrum of the gossypol in methanol-d_4_ under different storage conditions in different days (N: normal condition without protecting from air and light; L: under natural light and nitrogen protection; A: stored in dark and under nitrogen protection).

**Figure 3:**
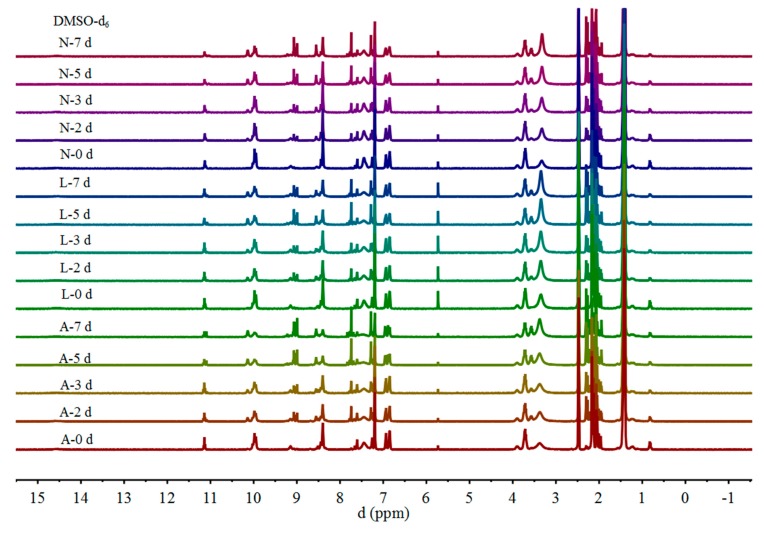
^1^H NMR spectrum of the gossypol DMSO-*d*_6_ solution under different storage conditions in different days (N: normal condition without protecting from air and light; L: under natural light and nitrogen protection; A: stored in dark and under nitrogen protection).

**Figure 4:**
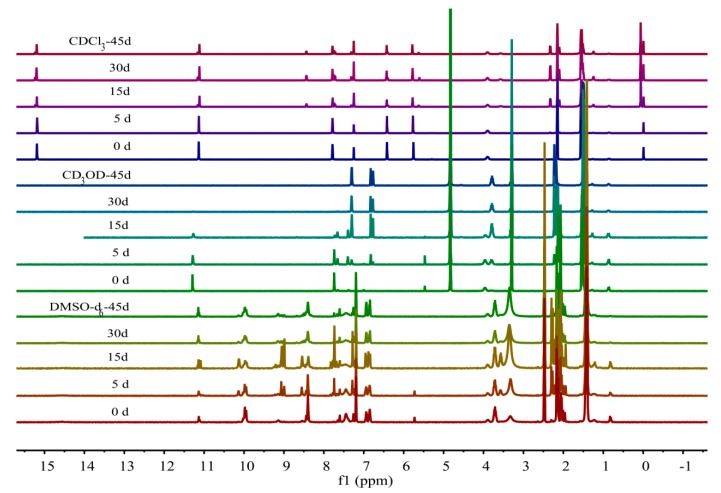
^1^H NMR spectra of the gossypol solutions under normal condition without protect from air and light storage conditions for different days.

According to the NMR study, ratios for the different tautomers to explain the tautomerization ratios under different conditions are elaborated in Table 4. The results are consistent with Figure 1, Figure 2, Figure 3 and Figure 4.

It was observed that proton signals did not change during 30 days and 45 days, and the general pattern was in stabilized lactol–lactol form. Considering that the toxicity of gossypol was believed to be from two aldehyde groups in the molecule [14], the stable lactol–lactol form could potentially reduce the toxicity of gossypol.

The ^1^H NMR spectrum of gossypol in different solvents over time showed that the tautomeric form had fundamental influences on gossypol instability. However, their prevalence and importance remained elusive, because they were found to exist only transiently [37,38]. Indeed, the different tautomeric form was also believed to be in equilibrium with each other in the fluid state [39,40,41,42].

### 2.2. Gossypol Solution Analyzed by UV-vis Measurement

**Figure 5:**
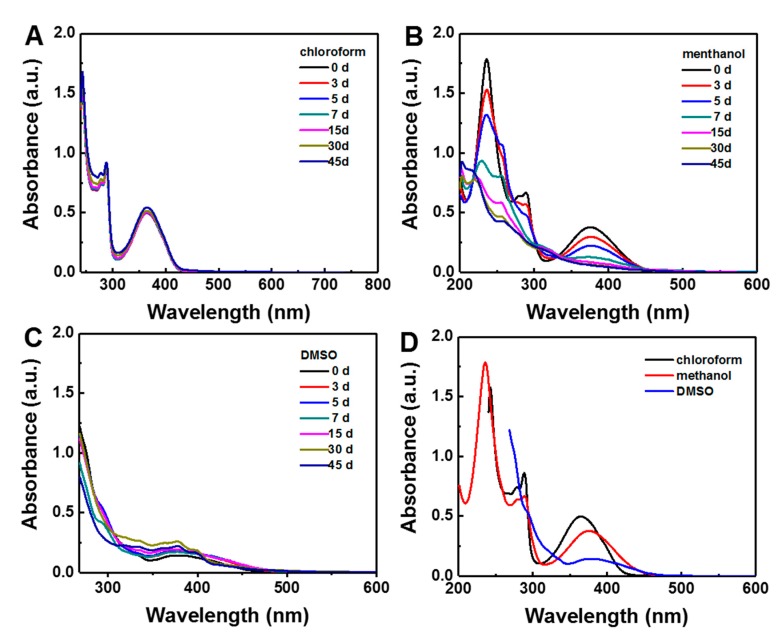
UV-vis spectra of the gossypol solution for different days (c = 0.025 mmol·L^−1^): (**A**) gossypol CHCl_3_ solution; (**B**) gossypol CH_3_OH solution; (**C**) gossypol DMSO solution; and (**D**) the absorption curve of gossypol in each solvent.

The position of the maximum absorbance and the molar absorption coefficient of gossypol significantly depended on the solvent used. The UV-Vis absorption spectra of gossypol in chloroform, methanol, and DMSO [23] over time are presented in Figure 5. As discussed in the previous part, ^1^H NMR showed that gossypol existed in aldehyde and lactol tautomeric form in chloroform. Gossypol existed in aldehyde and lactol form in methanol at day 30, and then was mainly in lactol form at day 45. Gossypol existed in aldehyde, lactol, and ketol forms in dimethyl sulfoxide. By comparison with the UV-Vis spectra of the freshly made gossypol solutions, as shown in Figure 5D, the B band spectra of π → π* at λ_max_ = 364 nm (gossypol in CHCl_3_) got shifted to λ_max_ = 380 nm (gossypol in DMSO) with the H-bonding interactions [43,44]. The pattern of the spectrum was similar for characteristic bands, and they mainly existed in aldehyde–aldehyde form. 

The UV-vis spectrum of gossypol in methanol changed significantly, as can be seen from Figure 5B. Additional storage with time in methanol solution led to the shape and intensity change of the three well-separated bands at 376 nm, 289 nm, and 236 nm. New bands at 311 nm, 254 nm, 219 nm, and 202 nm were observed after 15 days. This could be attributed to the lactol–lactol tautomer form [23]. It was also found that the long wavelength absorption band of π → π* observed at λ_max_ = 376 nm shifted to the lactol–lactol tautomeric form of gossypol (λ_max_ = 311 nm). After 30 days, the absorption spectra of gossypol actually ceased to change. This result was consistent with the ^1^H NMR data.

In the case of gossypol in chloroform, the spectra of freshly prepared solutions of gossypol in CH_3_OH and CHCl_3_ were very similar with three well-separated bands at 364 nm, 288 nm, and 242 nm. Gossypol in chloroform was found to be quite stable. The result showed that its formation of new bands was much slower than that in methanol. Taking these observations into account, one could conclude that gossypol in chloroform was fairly stable and mainly existed in aldehyde–aldehyde tautomeric forms over the period of 45 days. This result was also consistent with the ^1^H NMR data (Figures S9 and S10).

Gossypol in DMSO showed peaks at 380 nm, 347 nm, and 268 nm. There was no spectral shift observed over time. The 268-nm absorbance decreased within seven days and then increased after day 15. This could be attributed to the lactol tautomer, which mainly existed within seven days and then transformed to aldehyde form after 15 days, and the aldehyde tautomer existed until 30 days. However, by the time of 45 days, the lactol tautomer was predominating. 

Furthermore, the absorbance value at 263 nm at day 7 was less than that at day 45. This was possibly due to the formation of ketol form in small quantities in the DMSO after 15 days. The results implied that the lactol and aldehyde forms transformed into each other, and the ratio of lactol to aldehyde form was inversely proportional.

### 2.3. Gossypol Solution Analyzed by HPLC-QTOF-MS Measurements

**Figure 6:**
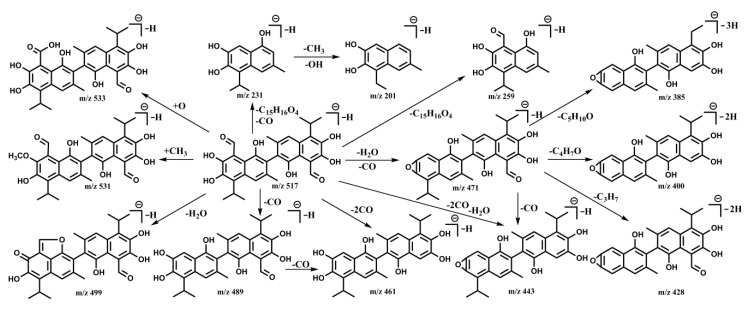
The main fragmentation pathway of gossypol molecular ion at *m*/*z* 531 and 517.

**Figure 7:**
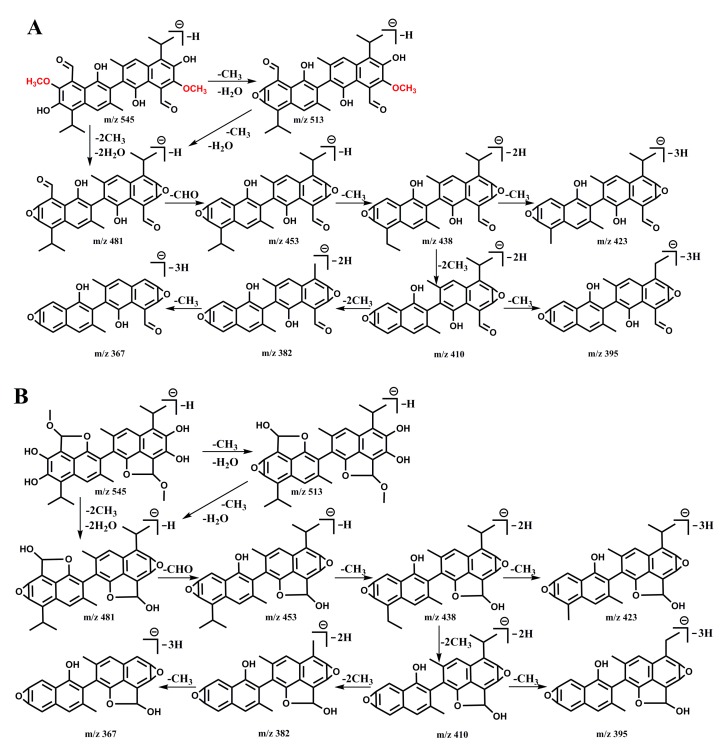
The two possible main fragmentation pathway of the gossypol molecular ion at *m*/*z* 545: (**A**) gossypol with aldehyde form was oxidized (**B**) gossypol with lactol form was oxidized.

For the HPLC-QTOF-MS analysis, the retention time depends on the molecular weight of analyte, and the fragment ion of gossypol can help explain the different structure of gossypol according to the fragmentation pathway. With negative ion mode, multistage ion mass spectra of gossypol fragments were screened according to fragment ion abundance, as shown in Table 5 (gossypol in chloroform) and Table 6 (gossypol in methanol). According to the fragment information of ions (MS^n^), the molecular structure of gossypol and its fragmentation pathway could be proposed [45,46] (Figure 6 and Figure 7). The molecular ion for gossypol [M − 1]^−^ (*m*/*z* 517) was the base peak (Table 5 and Table 6). 

The retention time of gossypol was found to be similar in each solvent over time at ambient temperature. The spectrum for freshly made samples of gossypol in chloroform and methanol were fairly simple. It showed two chromatographic peaks at the retention times of 8.65 min and 11.26 min for two the major ions [M + 16]^−^ ion (*m*/*z* 533) and the [M − 1]^−^ ion (*m*/*z* 517) (Supporting Information Figure S37). The major ions for gossypol in methanol were *m*/*z* 545, 531 and 517 at the retention times of 9.68 min, 10.69 min, and 10.61 min, respectively (Supporting Information Figure S50). Fragment ions were the [M + 16]^−^ ion (*m*/*z* 533), [M + 14]^−^ ion (*m*/*z* 531), and [M + 28]^−^ ion (*m*/*z* 545). The *m*/*z* 533, 531, and 545 ions were possibly due to the oxidation of the 15-CHO to –COOH and the oxidation of –OH to –OCH_3_. The common feature of all the HPHPLC-QTOF-MS results of gossypol over 30 days was the presence of the peaks at *m*/*z* 531 and 517, as well as fragmentation ions at *m*/*z* 485, 471, 459, 428, 259, and 231.

For gossypol in methanol, the major fragments were also found at *m*/*z* 481, 453, 438, 423, 410, 382, 395, 382, and 367; the *m*/*z* 453–367 ions probably resulted from the sequential loss of –CH_3_. The other fragments were at *m*/*z* 489, 471, 443, 259, and 231. The *m*/*z* 489, 471, and 443 ions could result from the loss of H_2_O and CO. The fragments at *m*/*z* 259 and 231 were an indication of the cleavage of the binaphthalene bond.

Some of the HPLC and MS data of gossypol have been reported [46,47,48,49,50,51,52]. The results of HPLC-QTOF-MS showed that the major ions and corresponding retention times were unchanged over the tested time period.

Based on the above results, one could conclude that the vibrating and bending of gossypol and interconversion of different tautomeric forms was highly dependent upon the solvents. With three different tautomeric forms, fresh gossypol in solvents initially existed as the aldehyde–aldehyde tautomer. After three to five days of forms, it transformed into the transition state, and then to form a more stable state according to the solvent. The infrared spectra showed that the transformation of the tautomeric forms of gossypol was dynamic in each solvent, whereas in chloroform, the aldehyde–aldehyde tautomer was predominant, and in methanol, the lactol–lactol tautomer was predominant after 15 days. 

In summary, previous work proposed that gossypol was not stable in various solvents. Some of the reported studies were based on experiments mostly using one single analytical method such as UV-vis or HPLC-QTOF-MS. Others were based on indirect characterization via Schiff bases derivatives. In the work reported here, a broad amount of spectroscopic techniques were combined together, and the influence of different storage conditions were also put into consideration and carefully investigated. Interestingly, results showed that among all the solvents and conditions studied, gossypol was found to be fairly stable in chloroform and the natural light. Atmospheric oxygen showed little effect on its stability. Although the spectra of gossypol in different solvents over time under different storage conditions indeed showed some or even significant changes, these changes were actually mainly due to the dynamic interconversion of the three tautomeric forms of gossypol rather than molecular decomposition and chemical degradation.

## 3. Materials and Methods 

### 3.1. Sample Preparation for Spectroscopy Analysis

Gossypol was purchased from Sigma-Aldrich (Shanghai, China). Methanol, chloroform, and dimethyl sulfoxide were all chromatographic grades unless otherwise notified. 

First, 5.0 mg of gossypol was dissolved in 0.70 mL of CDCl_3_, CD_3_OD, and DMSO-*d*_6_ in 5-mm quartz NMR tubes, respectively. The samples were stored under various conditions including: (1) under dark and with nitrogen protection; (2) natural light and with nitrogen protection; and (3) regular condition without protection from light and air. The samples were stored under a specific condition for 0 h, 8 h, 2 days, 3 days, 5 days, 7 days, 15 days, 30 days and 45 days at room temperature, and ^1^H NMR measurements were taken at different time intervals. 

Then, 15.0 mg of gossypol was dissolved in 3.0 mL of methanol, chloroform, and dimethyl sulfoxide in screw-capped vials, respectively. The samples were stored under regular condition without protection from light and air. The samples were stored under a specific condition for 0 days, 3 days, 5 days, 7days, 15 days, 30 days, and 45 days at room temperature, and HPLC-QTOF-MS and UV-vis measurements were taken at different time intervals.

### 3.2. ^1^H NMR Measurements

The ^1^H NMR spectra of gossypol were recorded on a VARIAN 400 MHz spectrometer (Varian, Palo Alto, CA, USA). The ^1^H NMR measurements of gossypol solutions were carried out at the operating frequency 400.22 MHz; scan times, nt = 32; line width 1 b = 1.5-Hz spectral width, sw = 6377.55 Hz; acquisition time, at = 1.28 s; T = 295.0 K; and TMS was used as the internal standard. 

### 3.3. UV-vis Measurements 

UV-vis measurements were taken with a Shimadzu UV-2600 double-beam UV-vis spectrophotometer (Shimadzu, Kyoto, Japan) equipped with 10-mm quartz absorption cells. The spectrums of the Gossypol solutions samples were recorded over the wavelength range of 200 to 600 nm.

### 3.4. HPLC-QTOF-MS Measurements 

High performance liquid chromatography quadruple time of flight mass spectrometry (HPLC-QTOF-MS) spectra was obtained with 4000 QSTAR Elite (AB SCIEX LLC, Redwood, CA, USA). The measurements were carried out using a model Agilent-HPLC system in combination with a model quadruple time of flight mass spectrometer. First, 5 μL of the diluted gossypol solutions was injected into a Phenomenex column (250 × 4.60 mm, Synergi 4u Hydro-RP 80A) at 30 °C with a flow rate of 300 μL·min^−1^. Acetonitrile (A) and 0.1% (*v*/*v*) formic acid in water (B) were used as mobile phase. Gossypol was eluted with a gradient of 50% A and 50% B (*v*/*v*) from 0 to 8 min followed by 100% A from 8 to 20 min.

Mass detection was carried out with electrospray ionization (ESI) in negative-ion full scan mode. The settings of the mass spectrometer were as follows: spray voltage, 4.5 kV; source temperature, 450 °C maximum injection time, 250 ms; scan rage, 100 to 1500. Nitrogen was used as a sheath gas (pressure 40 psi) and auxiliary gas (pressure 60 psi). The in-source collision-induced dissociation energy (CID) was set at 50 eV.

## 4. Conclusions

^1^H NMR, UV-vis spectroscopy, and HPLC-QTOF-MS spectrometry were used to investigate the stability of gossypol under different storage conditions. Gossypol was dissolved in three different solvents and stored under dark and nitrogen protection, natural light and nitrogen protection, and ambient air conditions at room temperature for different storage times up to as long as 45 days. Among all the solvents and conditions studied, gossypol was found to be fairly stable in chloroform, and the natural light and atmospheric oxygen have little effect on its stability. The freshly dissolved gossypol in chloroform was mainly in aldehyde–aldehyde form within five days; then, both aldehyde–aldehyde and lactol–lactol tautomeric forms existed until 45 days. Gossypol freshly dissolved in methanol was in aldehyde–aldehyde and ketol–ketol form. However, both aldehyde–aldehyde and ketol–ketol forms transformed into lactol–lactol form after 30 days. The freshly dissolved gossypol in dimethyl sulfoxide were in aldehyde–aldehyde, lactol–lactol, and ketol–ketol forms, and there was a reciprocal conversion between aldehyde–aldehyde and lactol–lactol forms over 45 days. Although the spectra of gossypol in different solvents over time under different storage conditions indeed showed some or even significant changes, these changes were actually mainly due to the dynamic interconversion of the three tautomeric forms of gossypol rather than molecular decomposition and chemical degradation. 

The spectroscopic data on the stability of gossypol could be useful for gossypol-based drug design, the biological activity of gossypol, and explaining the stability of gossypol derivatives for organic synthesis. 

## Figures and Tables

**Table 1:** ^1^H NMR chemical shifts (ppm) of gossypol in CDCl_3_ for different days under normal condition without protecting from air and light (ald: aldehyde, lac: lactol, ket: ketol).

Proton	Gossypol-CDCl_3_								
	0 days	1/3 days	2 days	3 days	5 days	7 days	15 days	30 days	45 days
H_12_,_12’_; H_13_,_13’_ (lac)		1.53	1.53	1.53	1.53	1.53	1.53	1.53	1.53
H_12_,_12_^’^; H_13_,_13’_ (ald)	1.56	1.56	1.56	1.56	1.56	1.56	1.56	1.56	1.56
H_14_, H_14’_ (lac)		2.34	2.34	2.34	2.34	2.34	2.34	2.34	2.34
H_14_, H_14_ (ald)	2.16	2.16	2.16	2.16	2.16	2.11, 2.16	2.11, 2.16	2.11, 2.16	2.11, 2.16
H_11_, H_11′_ (lac)		3.58	3.58	3.58	3.58	3.58	3.57	3.57	3.57
H_11_, H_11′_ (ald)	3.91	3.91	3.91	3.91	3.91	3.91	3.91	3.91	3.91
1-OH, 1′-OH	5.77	5.77	5.77	5.77	5.77	5.70, 5.82	5.64, 5.79	5.61, 5.79	5.64, 5.79
6-OH, 6′-OH	6.43	6.43	6.43	6.43	6.43	6.41 6.42	6.41, 6.42	6.41, 6.42	6.41, 6.44
H_4_, H_4′_ (lac)		7.32	7.32	7.32	7.32	7.32	7.33	7.28, 7.33	7.28, 7.33
H_4_, H_4′_ (ald)	7.79	7.79	7.79	7.79	7.79	7.74, 7.79	7.74, 7.79	7.74, 7.79	7.74, 7.79
15-CH 15′-CH (lac)		8.45	8.45	8.45	8.45	8.45	8.45	8.45	8.45
15-CHO	11.14	11.14	11.14	11.14	11.14	11.12 11.17	11.12, 11.17	11.12, 11.17	11.12, 11.17
7-OH, 7′-OH	15.20	15.20	15.20	15.20	15.20	15.19 15.24	15.19, 15.24	15.19, 15.24	15.19, 15.24

**Table 2:** ^1^H NMR chemical shifts (ppm) of gossypol in CD_3_OD for different days under normal condition without protecting from air and light.

Proton	Gossypol-CD_3_OD								
	0 day	1/3 day	2 days	3 days	5 days	7 days	15 days	30 days	45 days
H_12_; H_13_ (lac)							1.53	1.52	1.52
H_12_; H_13_ (ald)	1.57	1.57	1.56	1.56	1.56	1.55	1.57		
H_14_, H_14’_ (lac)			2.24	2.22, 2.26	2.24	2.24	2.24	2.22	2.22
H_14_, H_14’_ (ald)	2.14	2.14	2.14	2.12, 2.17	2.14	2.13	2.14		
H_11_, H_11′_ (lac)		3.84	3.84	3.83	3.84	3.83	3.84	3.82	3.81
H_11_, H_11′_ (ald)	4.01	4.01	4.00	4.00	4.00	3.99	4.00		
H_4_, H_4′_ (lac)		7.42	7.42	7.43	7.34, 7.43	7.34, 7.43	7.34, 7.43	7.32	7.32
H_4_, H_4′_ (ald)	7.69, 7.78	7.69, 7.78	7.70, 7.78	7.70, 7.78	7.70, 7.78	7.69, 7.77	7.70, 7.78		
H_4_, H_4′_ (ket)	7.01 6.99	7.01, 6.99	7.01, 6.99	7.01, 6.99	6.99	6.99			
15,15′-CH (lac)		6.86	6.86	6.81, 6.86	6.81, 6.86	6.81, 6.85	6.81, 6.86	6.79, 6.85	6.79, 6.85
15-CHO	11.32	11.33	11.32	11.32	11.32	11.31	11.32		
15-CH=OH	5.51	5.51	5.51	5.51	5.51	5.51			

**Table 3:** ^1^H NMR chemical shifts (ppm) of gossypol in DMSO-*d*_6_ for different days under normal condition without protecting from air and light.

Proton	Gossypol-DMSO-*d*_6_								
	0 days	1/3 days	2 days	3 days	5 days	7 days	15 days	30 days	45 days
H_12_,_12’_; H_13_,_13’_ (lac)	1.45	1.45	1.45	1.45	1.45	1.45	1.45	1.45	1.45
H_12_,_12_’; H_13_,_13’_ (ald)	1.43	1.43	1.43	1.43	1.43	1.43	1.43	1.43	1.43
H_14_, H_14’_ (lac)		2.31	2.31	2.31	2.25, 2.32	2.25, 2.32	2.25, 2.32	2.32	2.32
H_14_, H_14’_ (ald)	2.17	2.17	2.17	2.17	2.08, 2.18	2.08, 2.18	2.08, 2.18	2.08, 2.18	2.06, 2.18
H_14_, H_14’_ (ket)	2.08	2.04	2.04	2.04	1.98	1.98	2.08	1.98	2.08
H_11_, H_11′_ (lac)			3.59	3.59	3.59	3.59	3.59	3.59	3.59
H_11_, H_11′_ (ald)	3.74	3.74	3.74	3.74	3.74	3.74	3.74	3.74	3.74
H_11_, H_11′_ (ket)	3.93	3.93	3.93	3.93	3.93	3.93	3.93	3.93	3.93
1,1′-OH	7.47	7.46	7.46	7.46	7.46	7.46	7.49	7.47	7.49
6-OH,6′-OH	8.43, 8.47	8.43, 8.47	8.43, 8.47	8.43, 8.47	8.43, 8.58	8.43, 8.58	8.44, 8.56	8.45, 8.55	8.44, 8.56
H_4_, H_4′_ (lac)	7.22, 7.30	7.22, 7.30	7.22, 7.30	7.22, 7.30	7.22, 7.30	7.22, 7.30	7.22, 7.29	7.22, 7.30	7.22, 7.29
H_4_, H_4′_ (ald)	7.64, 7.80	7.64, 7.77	7.64, 7.77	7.64, 7.77	7.64, 7.77	7.64, 7.77	7.67, 7.78	7.67, 7.78	7.67, 7.78
15-CH,15′-CH(lactol)	6.88, 6.96	6.87, 6.96	6.88, 6.96	6.88, 6.96	6.88, 6.96	6.88, 6.96	6.89, 6.97	6.89, 6.97	6.89, 6.97
15-CHO	11.16, 11.13	11.16, 11.13	11.16, 11.13	11.16, 11.13	11.16, 11.13	11.16, 11.13	11.11, 11.17	11.11, 11.17	11.11, 11.17
7-OH,7′-OH	9.98, 10.00	9.98, 10.00	9.98, 10.00	9.98, 10.11	9.98, 10.16	9.98, 10.16	9.98, 10.16	9.98, 10.16	9.98, 10.16
15-CH=OH	5.75	5.75	5.75	5.75	5.75	5.75			
(15-OH-CH)(lac)	9.06, 9.16	9.06, 9.16	9.06, 9.16	9.02, 9.10	9.02, 9.10	9.02, 9.10	9.02, 9.09	9.02, 9.13	9.02, 9.09

**Table 4:** Ratio of gossypol tautomers for different days under normal condition without protecting from air and light.

	0 day	2 days	3 days	5 days	7 days	15 days	30 days	45 days
**CDCl_3_**	ald	ald:lac 98%:2%	ald:lac 98%:2%	ald:lac 97%:3%	ald:lac 91%:9%	ald:lac 89%:11%	ald:lac 85%:15%	ald:lac 80%:20%
**CD_3_OD**	ald:ket 92%:8%	ald:lac:ket 79%:16%:5%	ald:lac:ket 72%:18%:10%	ald:lac:ket 66%:31%:3%	ald:lac:ket 55%:41%:4%	ald:lac 22%:78%	lac	lac
**DMSO-*d*_6_**	ald:lac:ket 20%:74%:6%	ald:lac:ket 29%:65%:6%	ald:lac:ket 30%:66%:4%	ald:lac:ket 26%:69%:5%	ald:lac:ket 34%:64%:2%	ald:lac 39%:61%	ald:lac 26%:74%	ald:lac 28%:72%

**Table 5:** The MS^n^ fragment ion of gossypol in CHCl_3_ for different days.

Gossypol in CHCl_3_			
Time	MS^1^ (*m*/*z*)	t_R_ (min)	MS^2^ (*m*/*z*)
0 day	533.20	8.65	
	517.21	11.32	
3 days	533.22	8.67, 9.34	515.21, 487.23, 472.17, 462.17, 459.22, 444.19, 434.17, 429.18, 417.18, 401.16, 385.13, 275.11, 257.10, 232.11, 231.12, 203.05, 188.07
	531.21	9.86,10.6	503.22, 485.20, 460.15, 457.20, 443.15, 430.58, 401.15, 274.10, 272.09, 259.12, 244.09, 232.12, 231.12, 203.05, 188.06, 179.08
	517.23	11.32	471.22, 461.23, 259.12, 231.12, 232.12, 201.11
5 days	531.28	10.33	485.25, 460.28, 273.12, 257.10, 245.13, 231.14, 179.13
	517.24	11.31	489.33, 471.22, 461.27, 443.31, 428.28, 259.11, 232.09, 231.13, 215, 201, 188.08
7 days	531.16	10.66, 12.08	485.17, 471.21, 459.23, 273.07, 257.05, 245.09, 231.10, 203.09, 179.10
	517.14	11.41	489.20, 471.15, 461.18, 443.20, 428.14, 260, 10, 259.09, 231.09, 201.09, 188.05
15 days	531.20	10.47, 12.02	485.20, 459.25, 273.09, 257.07, 245.10, 231.11, 179.16
	517.21	11.3	489.24, 471.20, 461.22, 443.25, 428.20, 259.11, 232.12, 231.11, 201.10, 188.06
30 days	531.21	9.87, 10.54	503.20, 485.20, 459.21, 401.13, 273.10, 257.16, 245.22, 231.12
	517.20	11.42	471.20, 461.23, 259.11, 232.11, 231.11, 201.11, 188.07

**Table 6:** The MS^n^ fragment ion of Gossypol in CH_3_OH for different days.

Gossypol in CH_3_OH			
Time	MS^1^ (*m*/*z*)	t_R_ (min)	MS^2^ (*m*/*z*)
0 day	545.28	9.68	
	531.21	10.69	
	517.22	11.61	
3 days	545.20	9.72	513.20, 481.14, 453.15, 438.14, 423.13, 410.11, 395.12, 382.14
	531.19	10.83,12.09	499.21, 471.15, 443.18, 428.13, 400.19, 273.09, 257.07, 245.11, 231.10
	517.14	11.06,	489.20, 471.15, 461.18, 443.19, 428.14, 259.08, 231.09, 232.09, 215.07, 188.04
5 days	545.30	9.75	481.21, 453.24, 438.22, 423.23, 410.20, 395.21, 382.26
	531.19	10.87	471.23, 443.30, 428.22
	517.26	11.67	489.32, 471.22, 461.27, 443.31, 428.27, 259.12, 232.13, 230.12
7 days	545.19	9.68,9.87	513.21, 481.14, 438.12, 423.12, 410.11, 395.12, 382.14, 367.16
	531.18	10.78, 11.00	499.22, 471.23, 443.30, 428.24
	517.14	11.53,12.01	501.00, 499.00
15 days	545.25	9.75	513.25, 481.19, 453.20, 438.18, 423.16, 410.17, 395.16, 382.18, 367.16
	531.24	10.86	485.35, 471.21, 443.25, 428.20, 273.11, 257.11, 245.13, 231.12
	517.20	11.59	471.21, 461.24, 259.11, 232.12, 231.11, 215.10, 201.11, 188.08
30 days	545.23	9.74,11.3	481.18, 453.19, 438.16, 410.16, 395.19
	531.24	10.92,	503.24, 491.24
	517.23	11.55	441.18, 413.21
	577.00	6.74,8.73	485.33, 469.22, 453.18

## Supplementary Materials

Additional tables and figures can be found in Supporting Information.
