# Supplementary material for: Investigating Stability and Tautomerization of Gossypol—A Spectroscopy Study
Source: Molecules. 2019 Apr 2;24(7):1286. doi: 10.3390/molecules24071286 (PMC6479638; doi:10.3390/molecules24071286)
Supplement: Supplementary file 1 [file molecules-24-01286-s001.pdf]

Supporting Information

Investigating stability and tautomerization of gossypol - a spectroscopy study

Lulu Wang[1, 2], Yanxia Liu[1, 2], Yagang Zhang*[,1, 2,3], Akram Yasin [1], Letao Zhang[1]

[1] Xinjiang Technical Institute of Physics and Chemistry, Chinese Academy of Sciences, Urumqi 830011, China; wanglulu@ms.xjb.ac.cn (L.W.); ygzhang@ms.xjb.ac.cn (Y.Z.); zhanglt@ms.xjb.ac.cn (L.Z.); akram@ms.xjb.ac.cn (A.K.); liuyanxia@ ms.xjb.ac.cn (L.Y.);

[2] University of Chinese Academy of Sciences, Beijing 100049, China

[3] Department of chemical and environmental engineering, Xinjiang Institute of Engineering, Urumqi 830026, China

* Correspondence: ygzhang@ms.xjb.ac.cn; Tel.: +86-18129307169

1.^{1}H NMR spectrum of Gossypol solutions. S2

2. UV-VIS absorbance spectra of Gossypol solutions. S17

3. ART-FTIR spectra of Gossypol solutions S20

4. HPLC-QTOF-MS spectrum of Gossypol solutions S25

1.^{1}H NMR spectrum of Gossypol solutions.

Sample Preparation for ^1H NMR Spectroscopy Analysis: 5.0 mg of gossypol was dissolved in 0.70 mL of $CDCl_3$, CD_3OD and DMSO-d_6 in 5 mm NMR tube respectively. The samples were stored under various conditions including: (1) N: normal condition without protecting from air and light; (2) L: under natural light and nitrogen protection; (3) A: stored in dark and under nitrogen protection condition. The samples were stored under a specific condition for 0 h, 8 h, 2 days, 3 days, 5 days, 7days, 15 days, 30 days and 45 days at room temperature and ^1H NMR measurements were taken at different time intervals.

The ^1H NMR spectra of gossypol were recorded on a VARIAN 400 MHz spectrometer. The ^1H NMR measurements of gossypol solutions were carried out at the operating frequency 400.22 MHz; scan times, nt=32; line width 1b=1.5Hz spectral width, sw=6377.55Hz; acquisition time, at =1.28s; T=295.0 K and TMS was used as the internal standard.

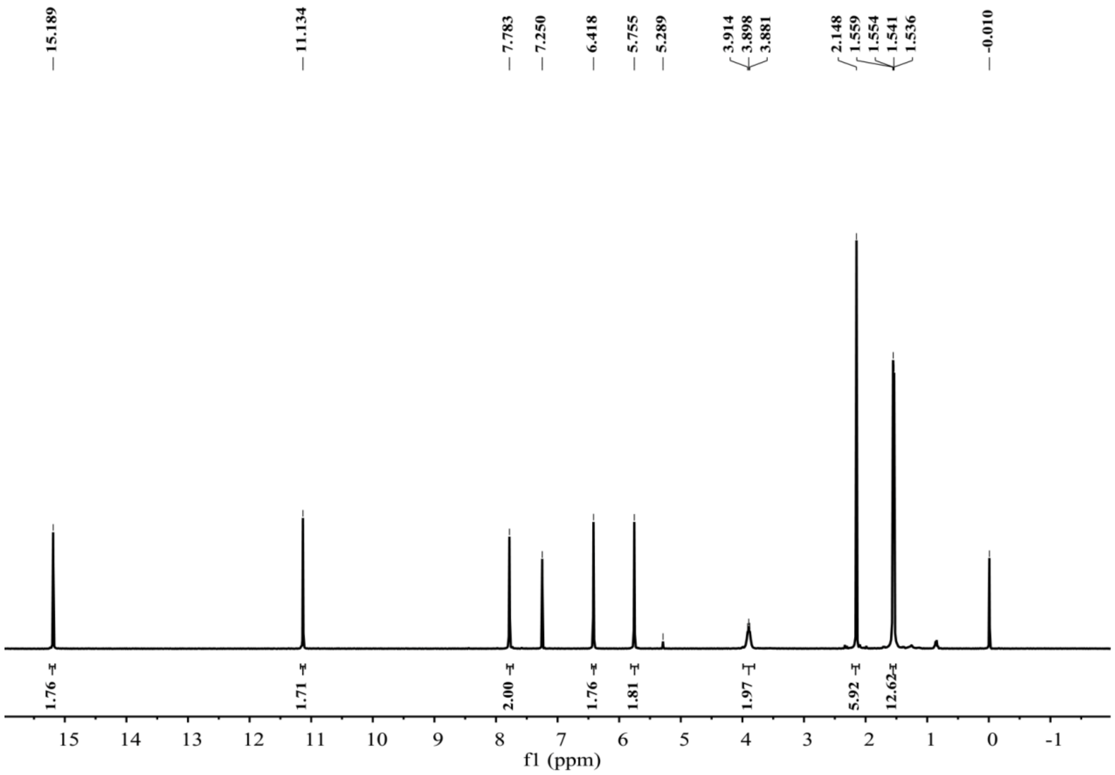

Figure S1. ^1H NMR spectrum of Gossypol-$CDCl_3$ fresh solution under normal condition without protect from air and light storage condition.

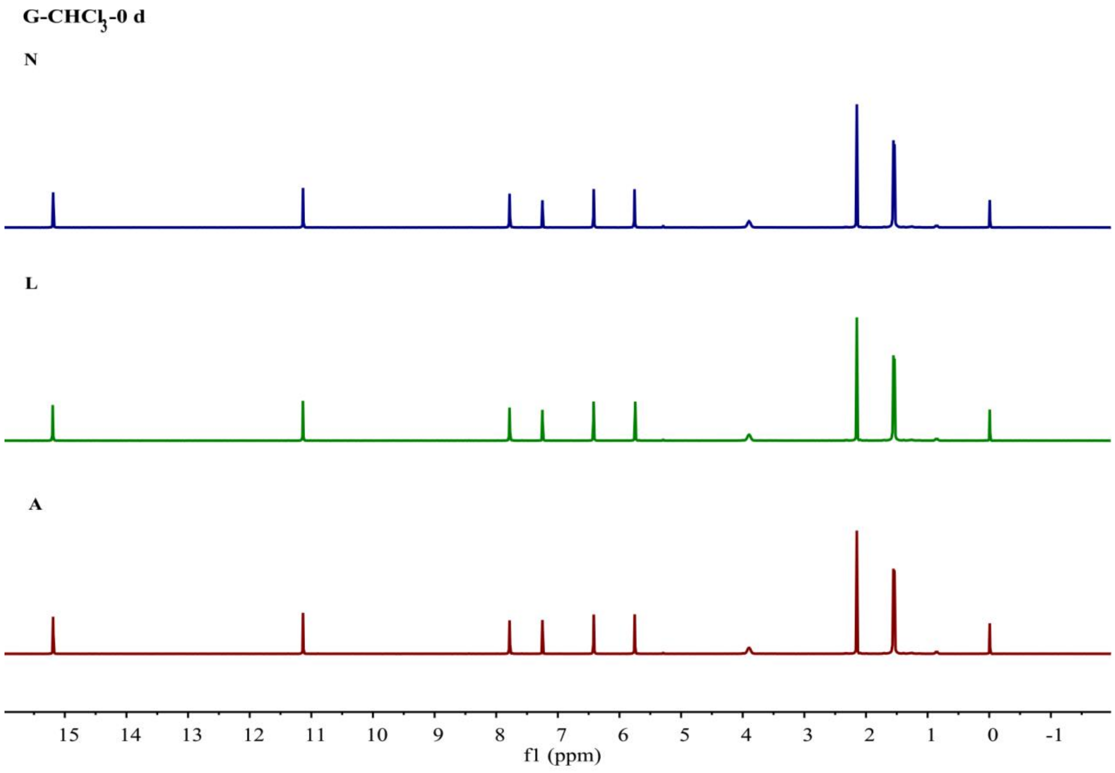

Figure S2. ^{1}H NMR spectrum of the gossypol in $CDCl_3$ solution under different storage conditions in 0 day (N: normal condition without protecting from air and light; L: under natural light and nitrogen protection; A: stored in dark and under nitrogen protection condition).

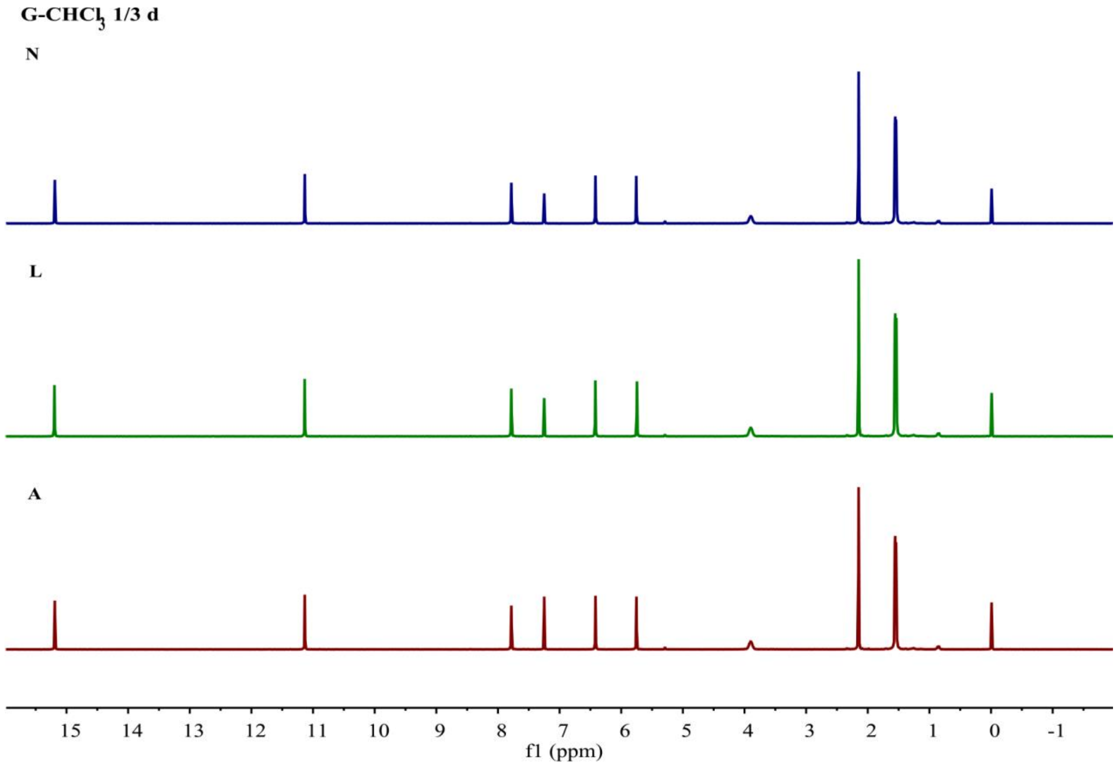

Figure S3. ^{1}H NMR spectrum of the gossypol in $CDCl_3$ solution under different storage conditions in 1/3 days.

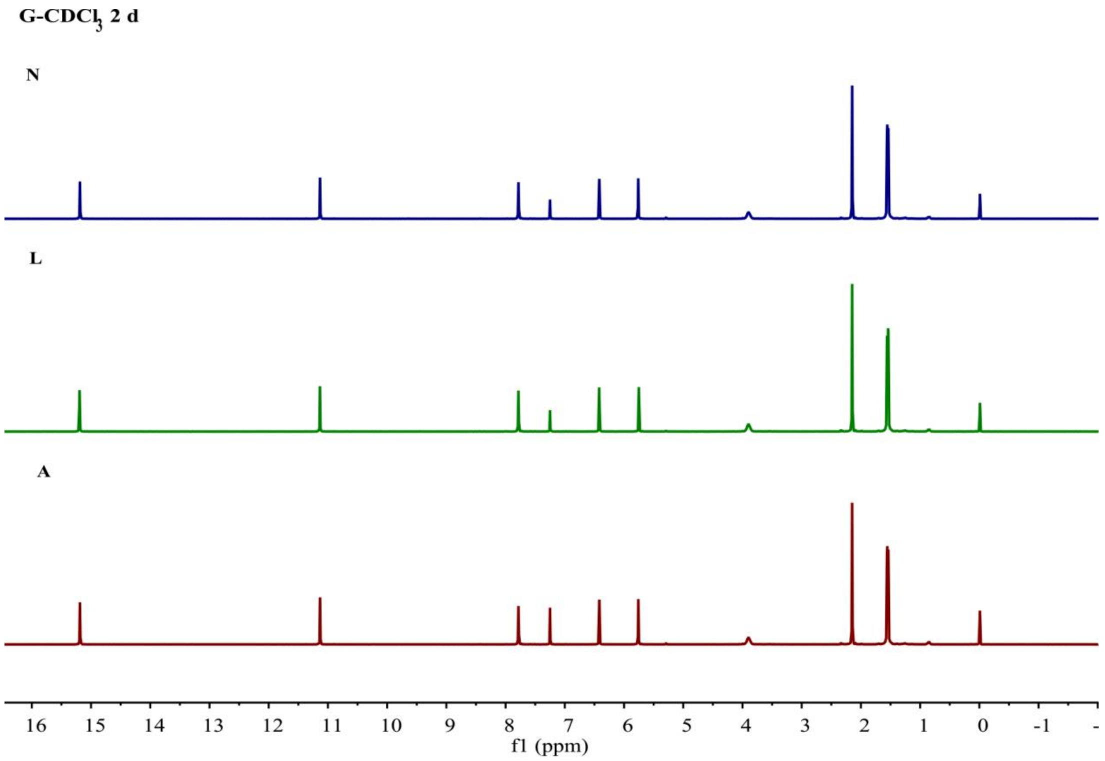

Figure S4. ^{1}H NMR spectrum of the gossypol in $CDCl_3$ solution under different storage conditions in 2 days.

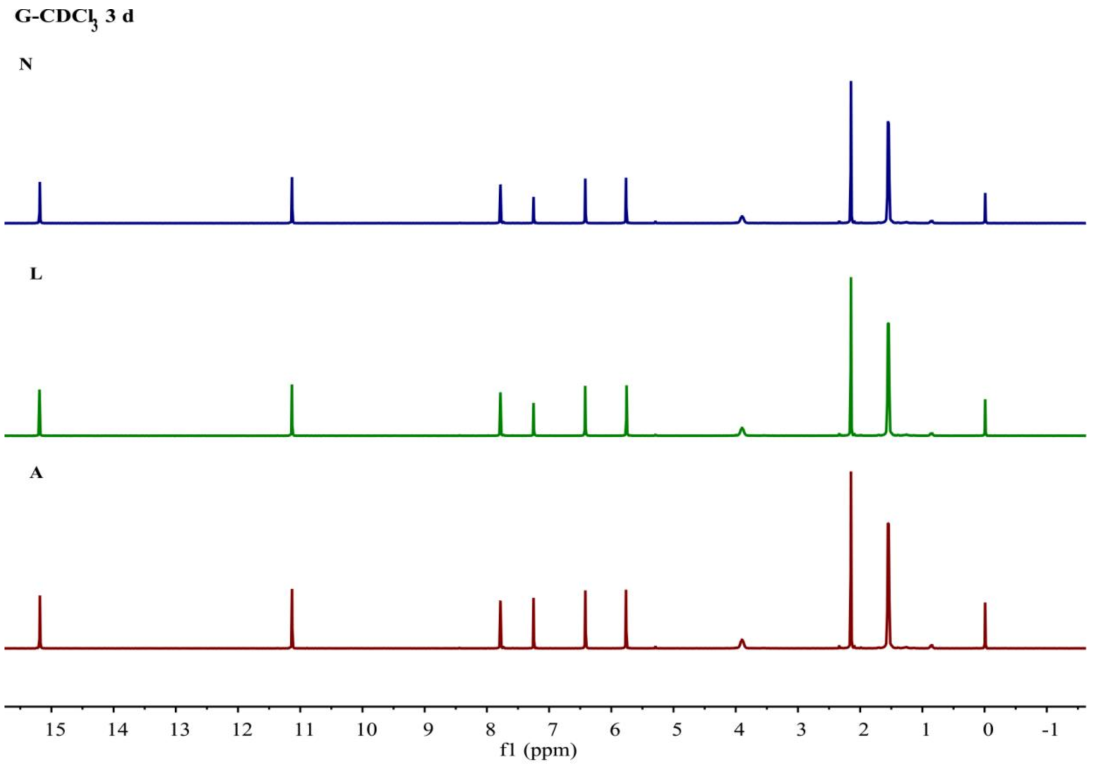

Figure S5. ^{1}H NMR spectrum of the gossypol in $CDCl_3$ solution under different storage conditions in 3 days.

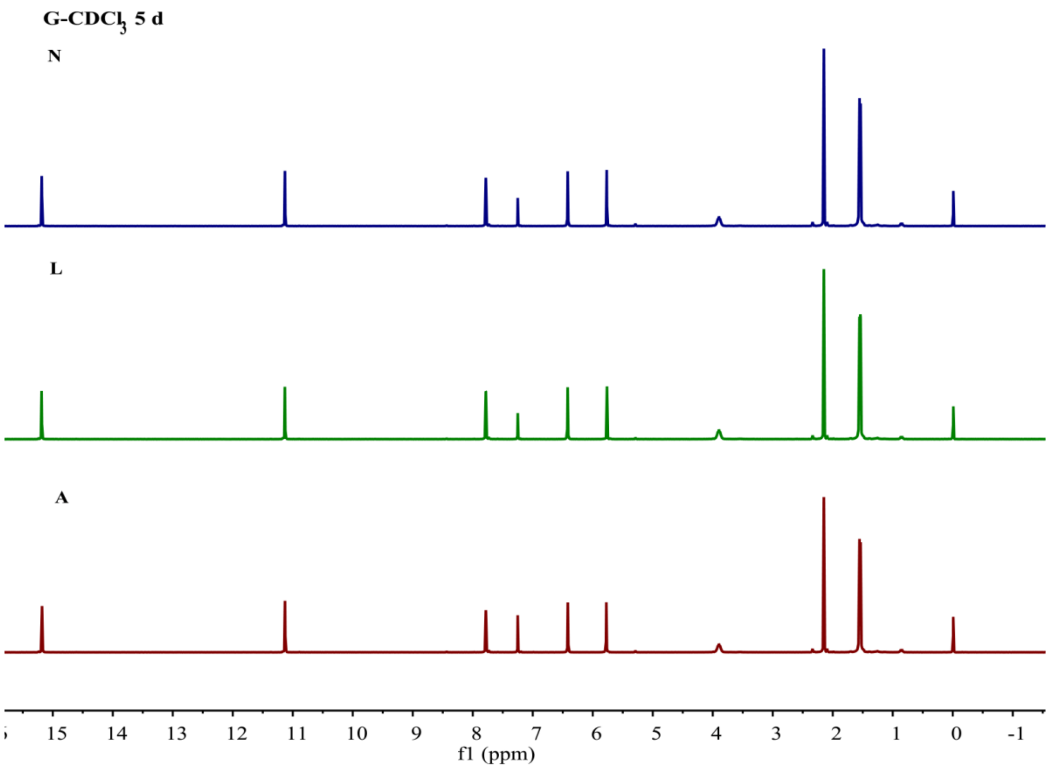

Figure S6. ^{1}H NMR spectrum of the gossypol in $CDCl_3$ solution under different storage conditions in 5 days.

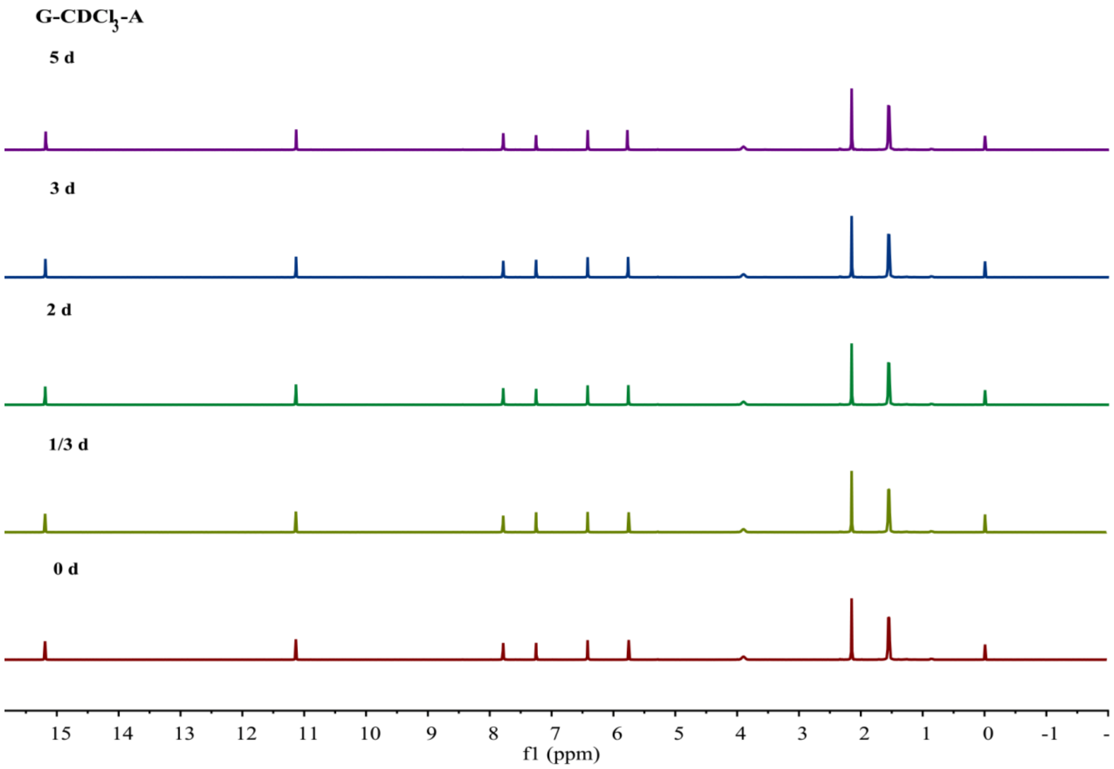

Figure S7. ^{1}H NMR spectrum of the gossypol in $CDCl_3$ solution under stored in dark and under nitrogen protection storage condition in different days.

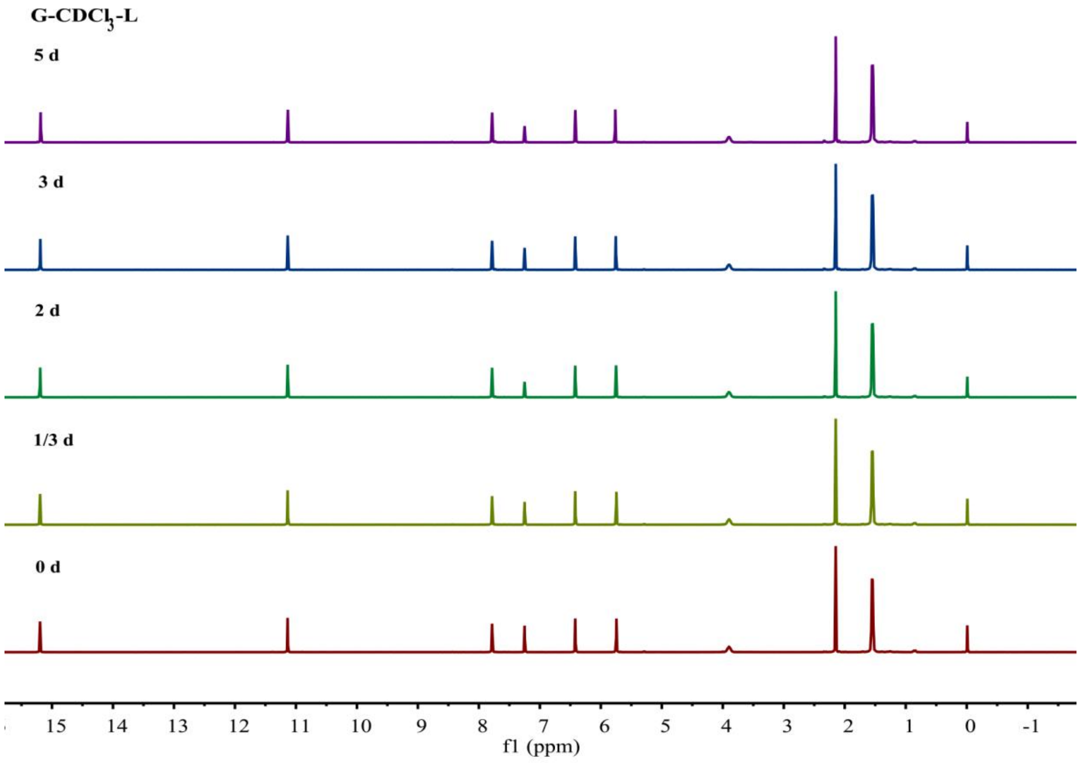

Figure S8.^{1}H NMR spectrum of the gossypol in $CDCl_3$ solution under natural light and nitrogen protection storage condition in different days.

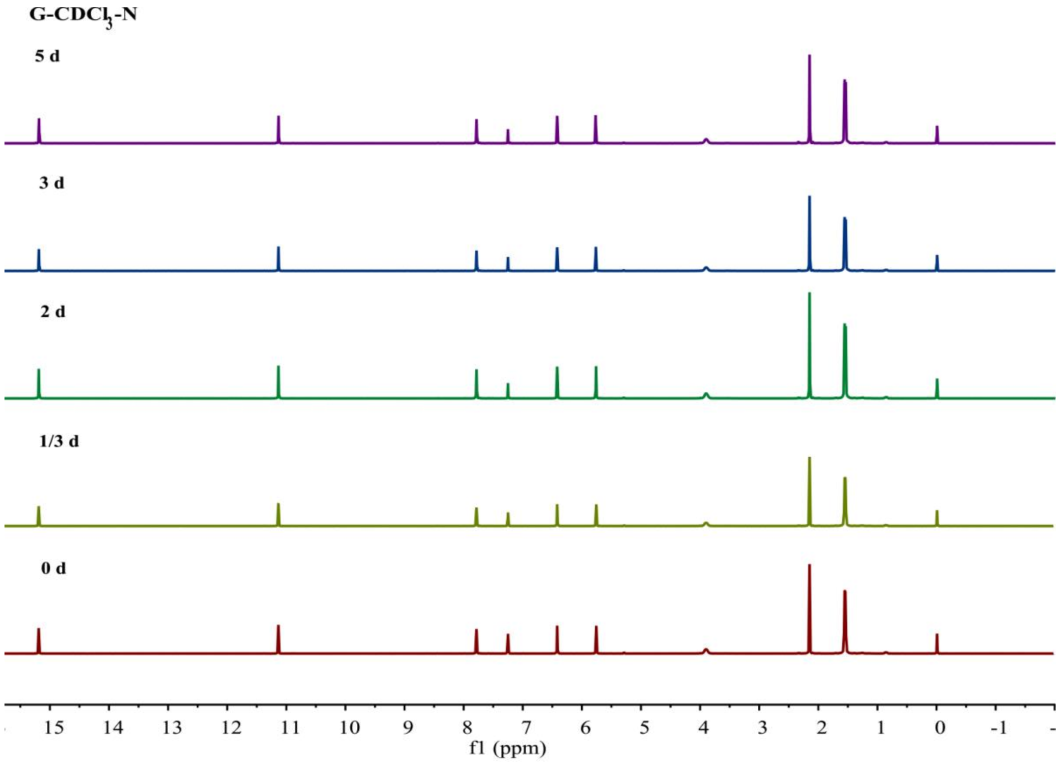

Figure S9. ^{1}H NMR spectrum of the gossypol in $CDCl_3$ solution under normal condition without protect from air and light storage condition in different days (0-5days).

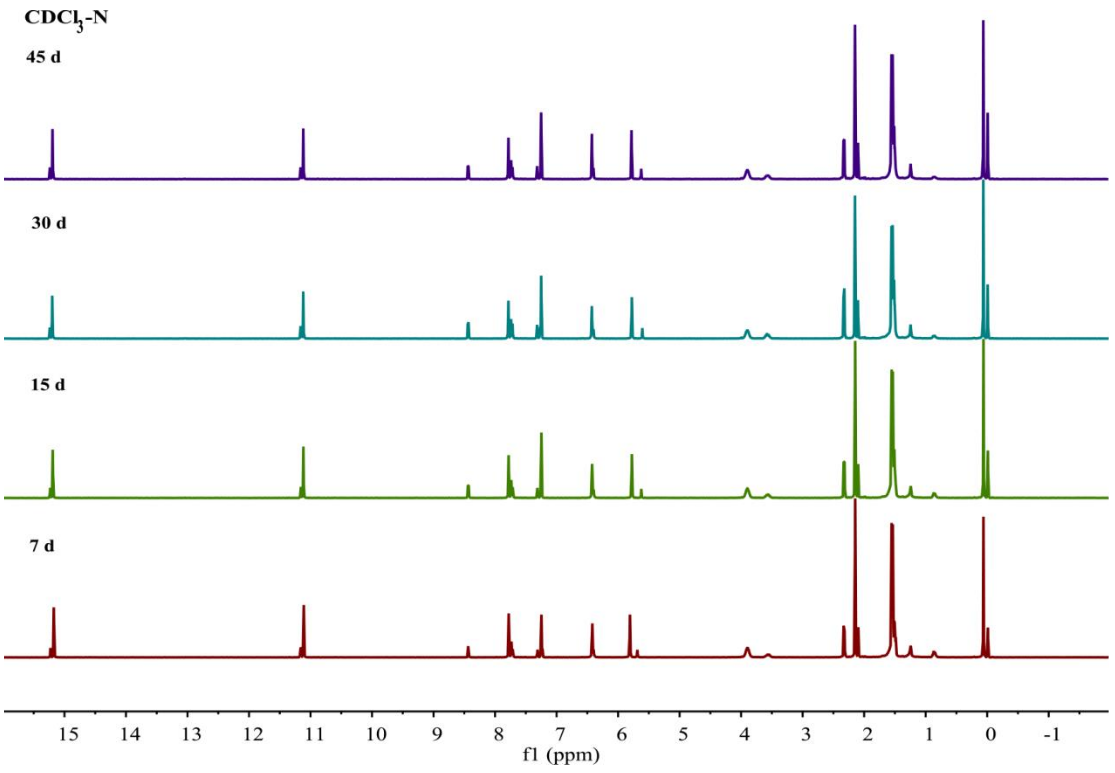

Figure S10. ^{1}H NMR spectrum of the gossypol in $CDCl_3$ solution under normal condition without protect from air and light storage condition in different days (7-45days).

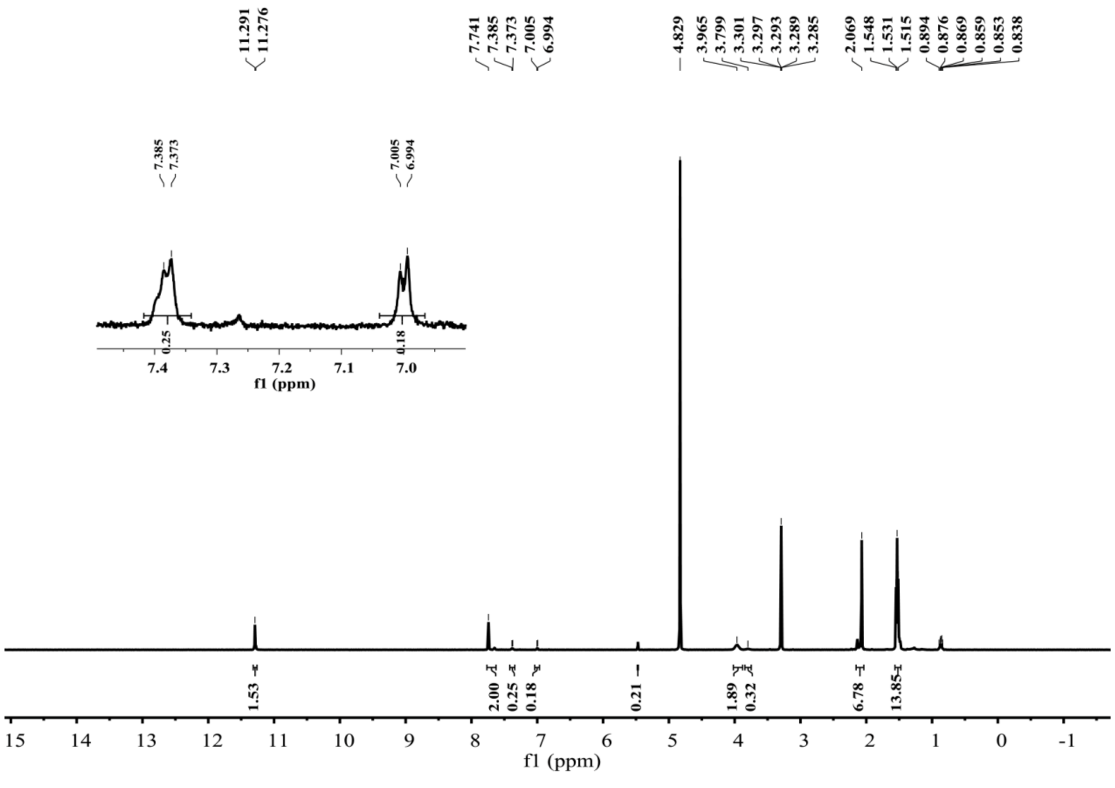

Figure S11. ^{1}H NMR spectrum of Gossypol-CD_3OD fresh solution under normal condition without protect from air and light storage condition.

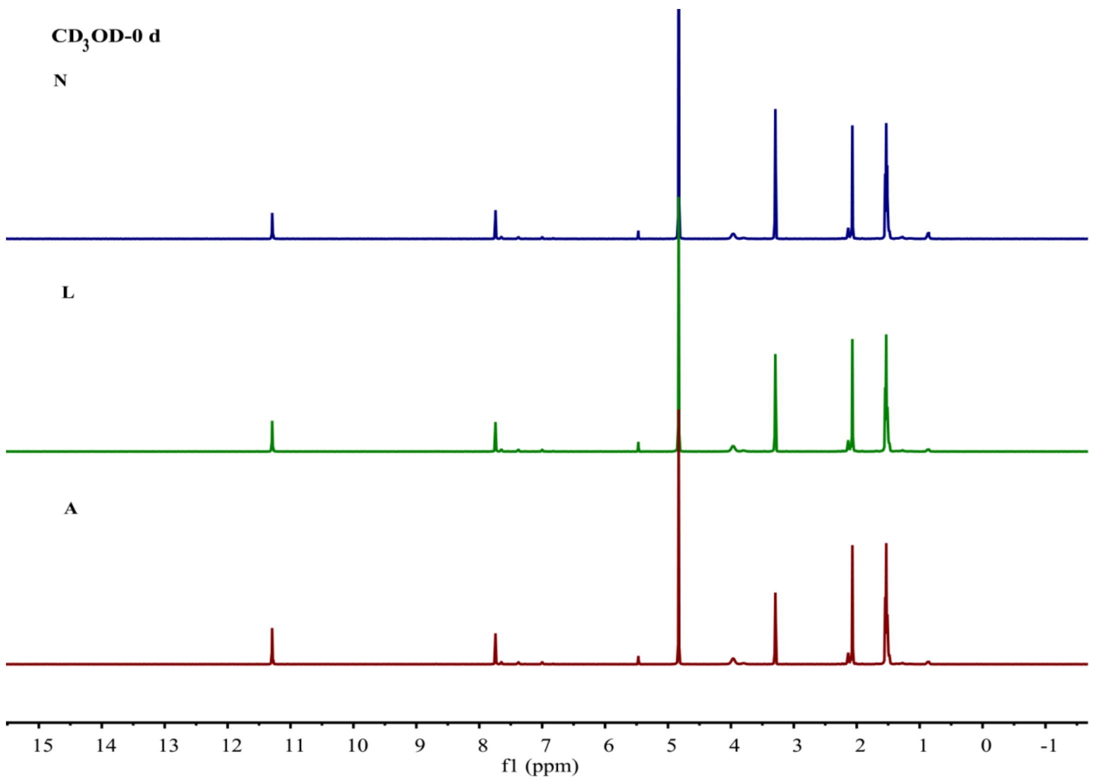

Figure S12. ^{1}H NMR spectrum of the gossypol in CD_3OD solution under different storage conditions in 0 day (N: normal condition without protecting from air and light; L: under natural light and nitrogen protection; A: stored in dark and under nitrogen protection condition).

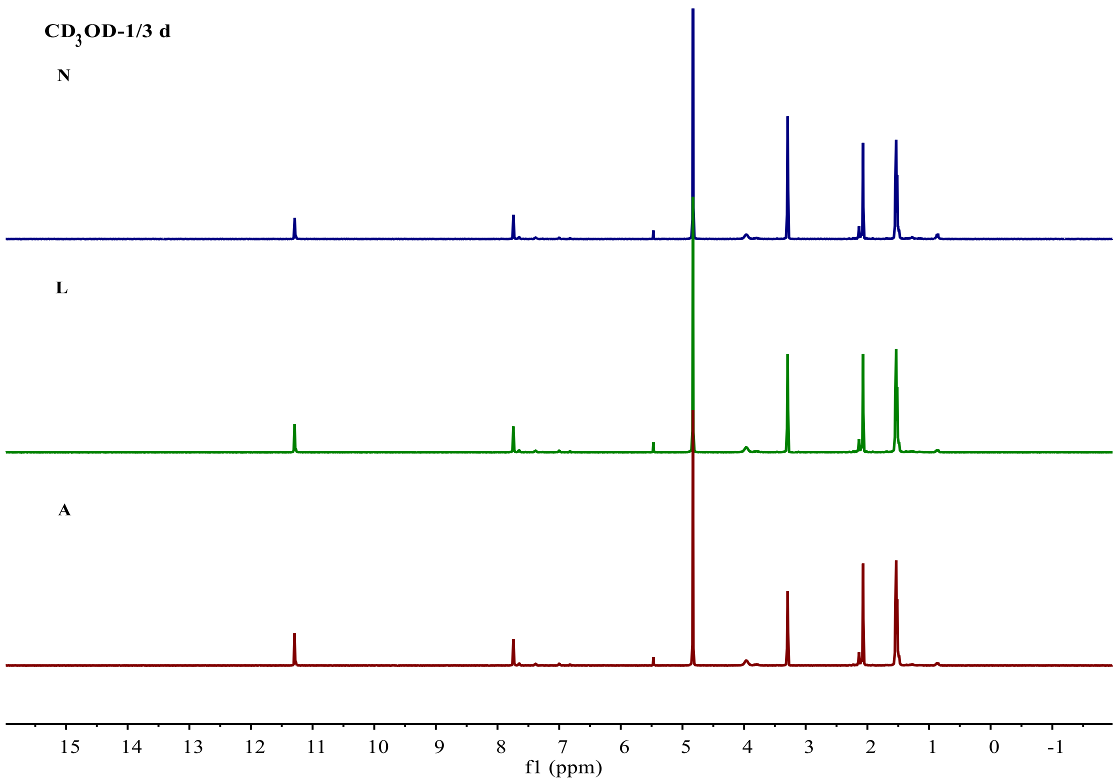

Figure S13. ^{1}H NMR spectrum of the gossypol in CD_3OD solution under different storage conditions in 1/3 days.

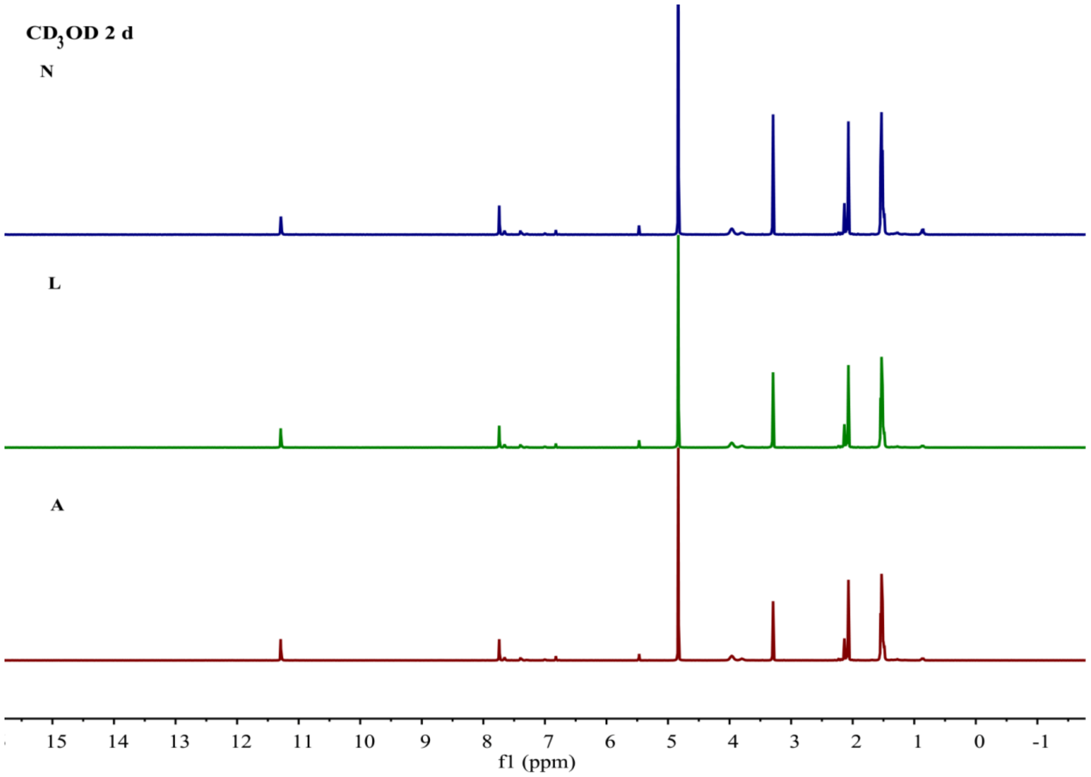

Figure S14. ^{1}H NMR spectrum of the gossypol in CD_3OD solution under different storage conditions in 2 days.

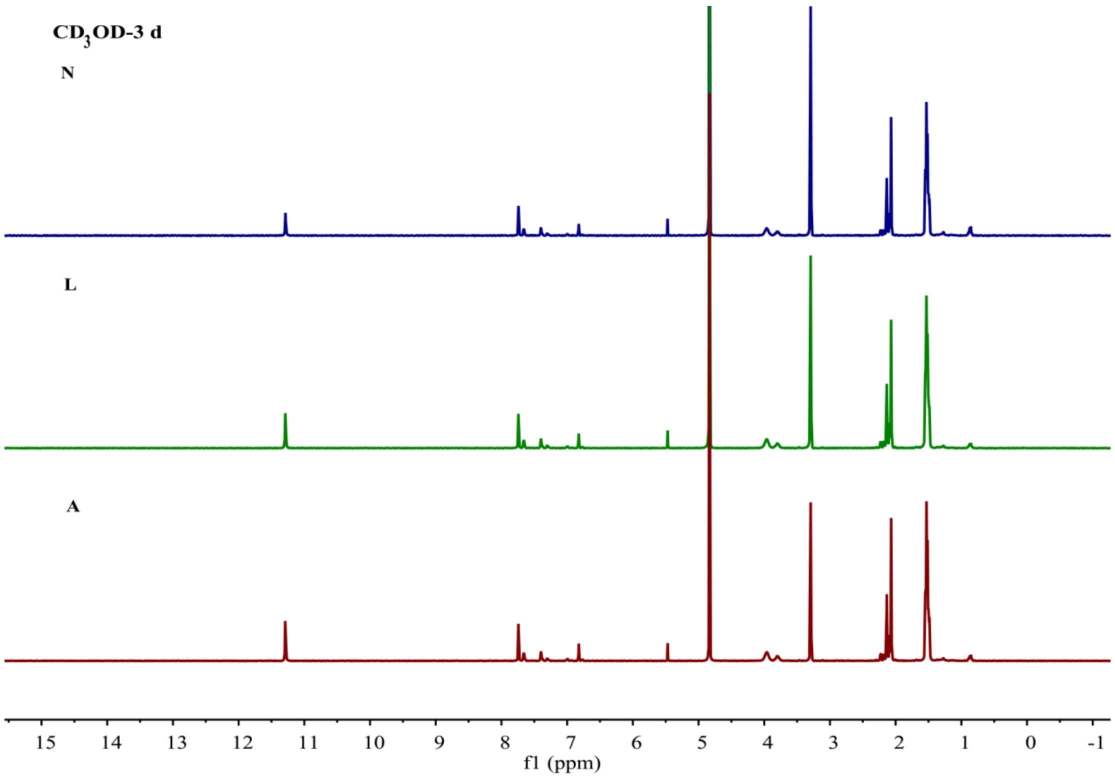

Figure S15. ^{1}H NMR spectrum of the gossypol in CD_3OD solution under different storage conditions in 3 days.

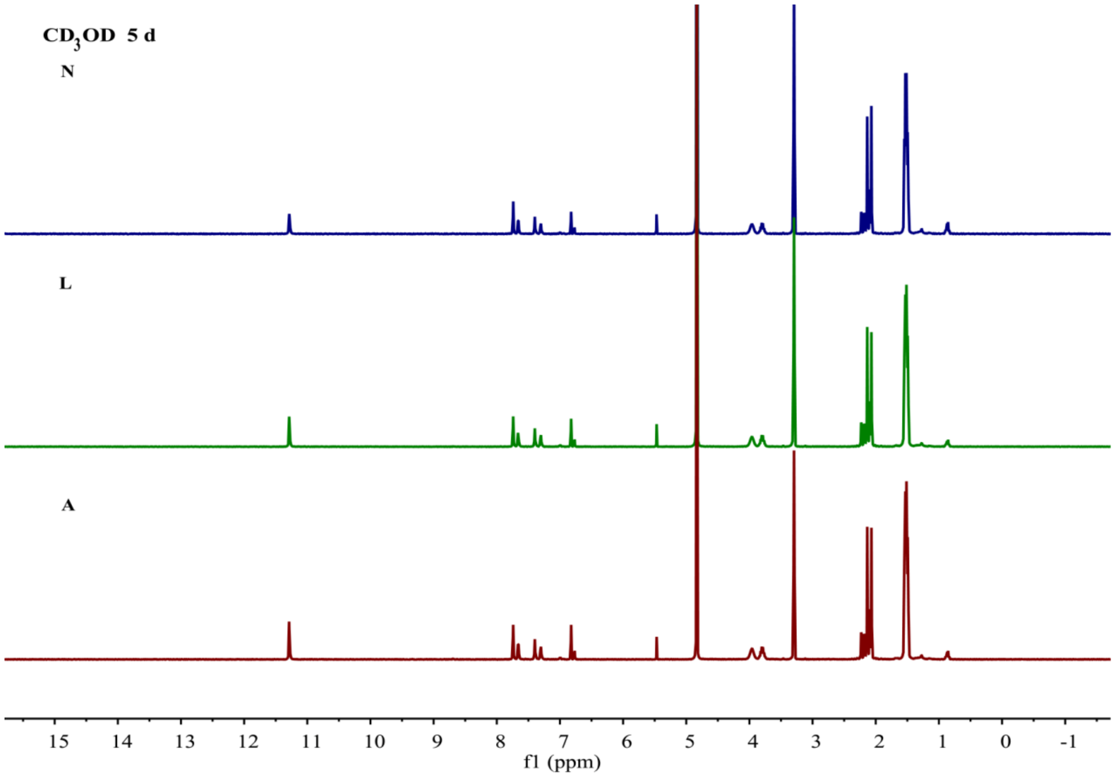

Figure S16. ^{1}H NMR spectrum of the gossypol in CD_3OD solution under different storage conditions in 5 days.

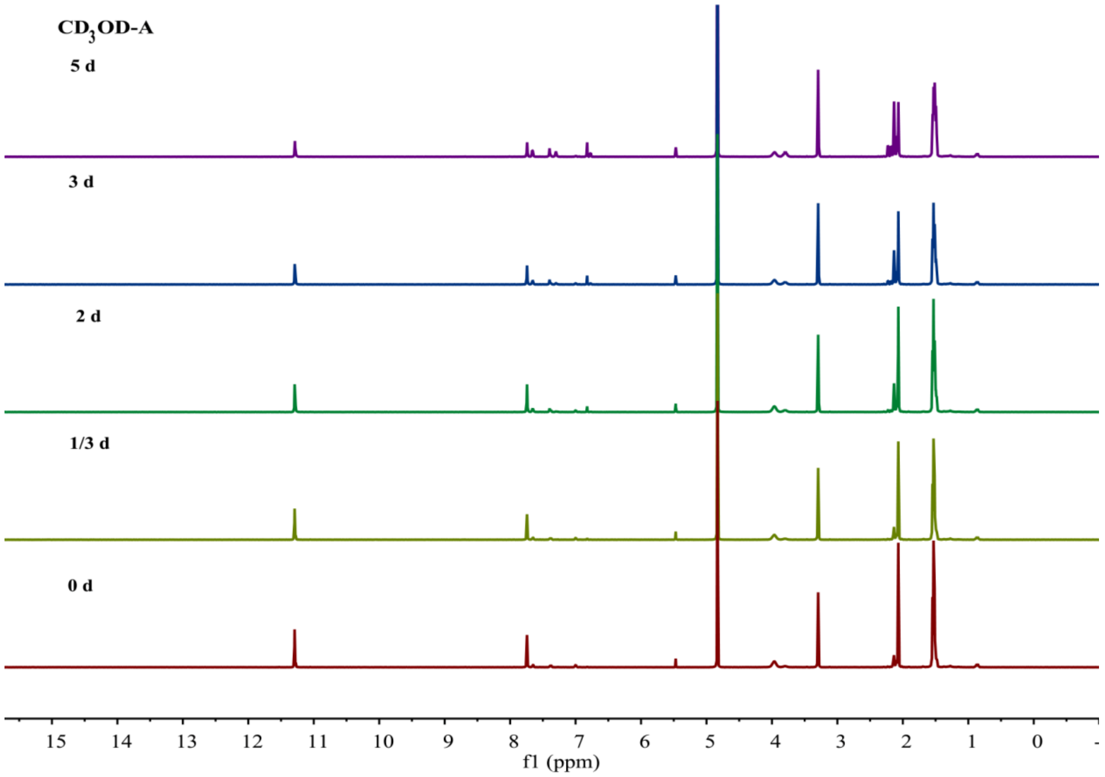

Figure S17. ^{1}H NMR spectrum of the gossypol in CD_3OD solution under stored in dark and under nitrogen protection condition storage condition in different days.

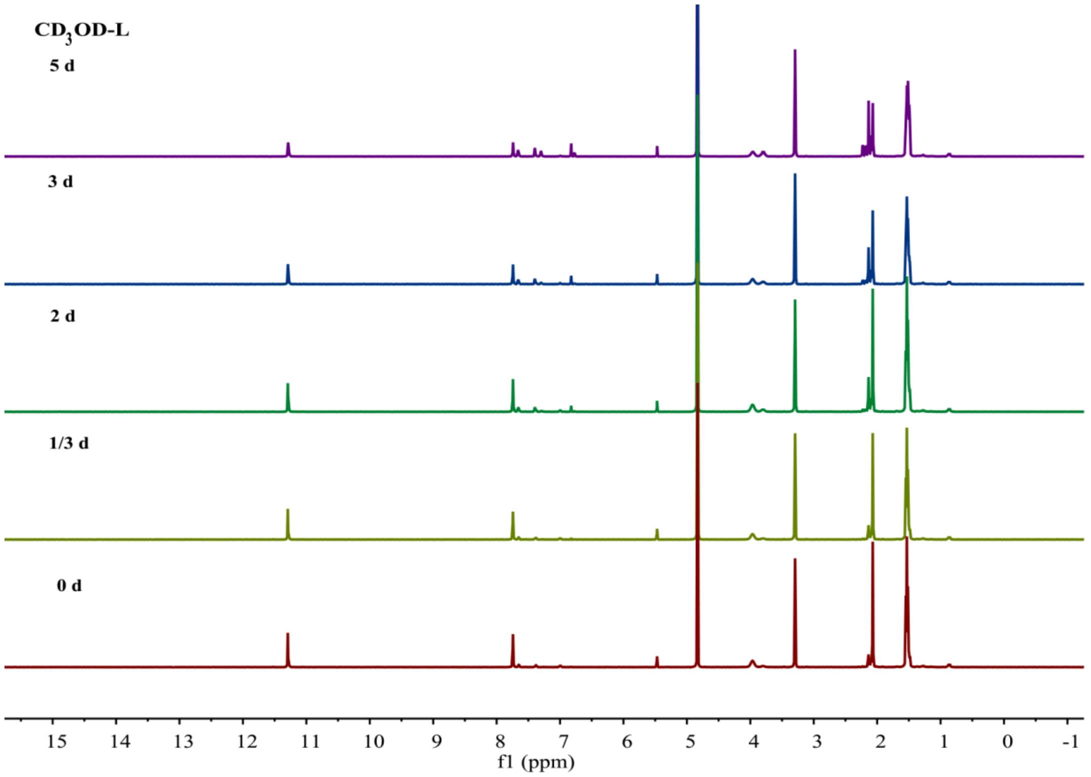

Figure S18.^{1}H NMR spectrum of the gossypol in CD_3OD solution under natural light and nitrogen protection storage condition in different days.

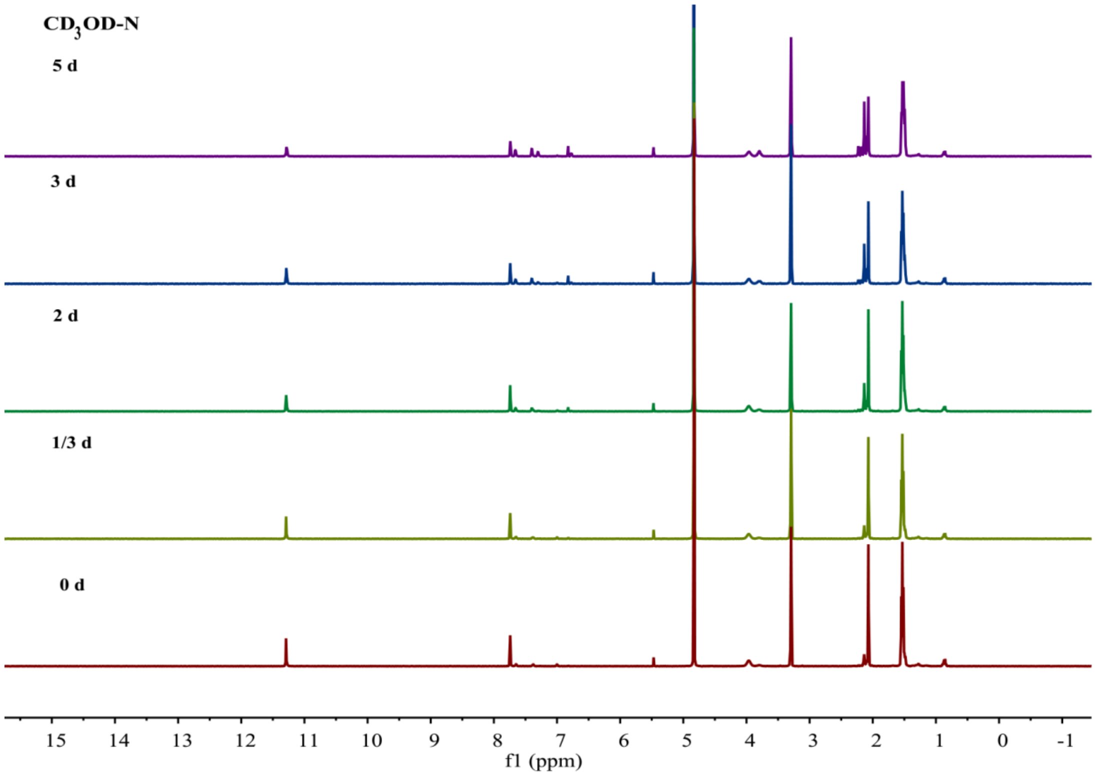

Figure S19. ^{1}H NMR spectrum of the gossypol in CD_3OD solution under normal condition without protect from air and light storage condition in different days (0-5 days).

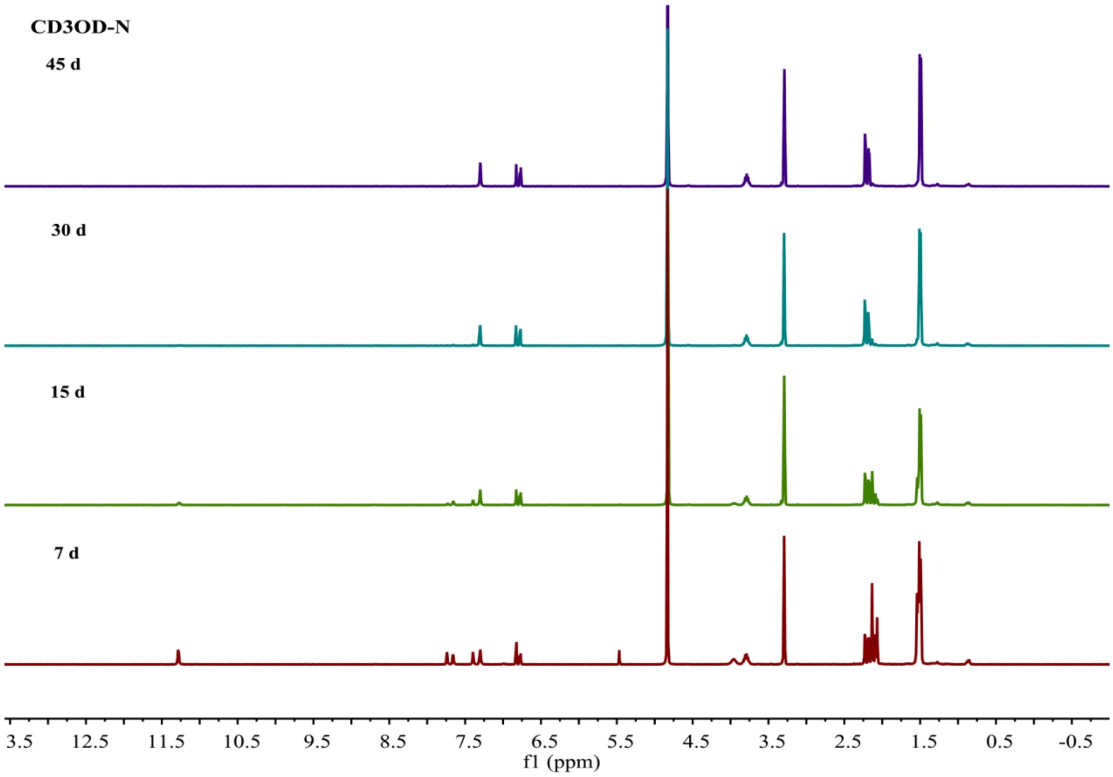

Figure S20. ^{1}H NMR spectrum of the gossypol in CD_3OD solution under normal condition without protect from air and light storage condition in different days (7-45 days).

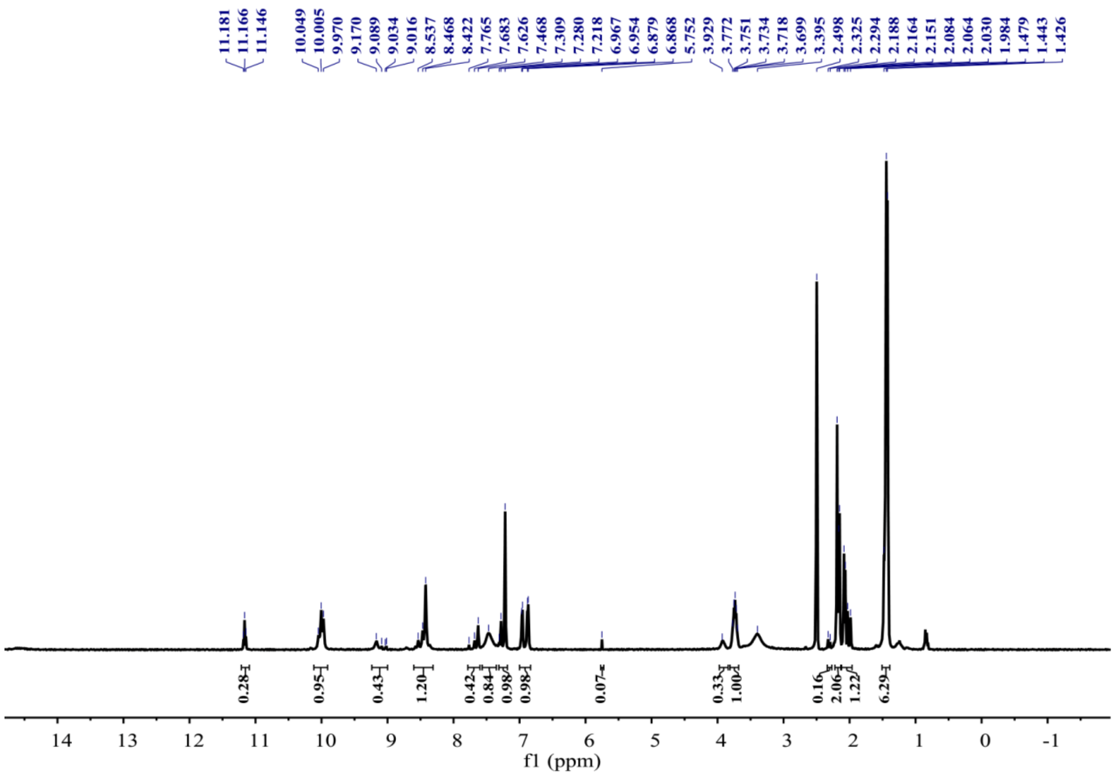

Figure S21. ^{1}H NMR spectrum of Gossypol- DMSO-d_6 fresh solution under normal condition without protect from air and light storage condition.

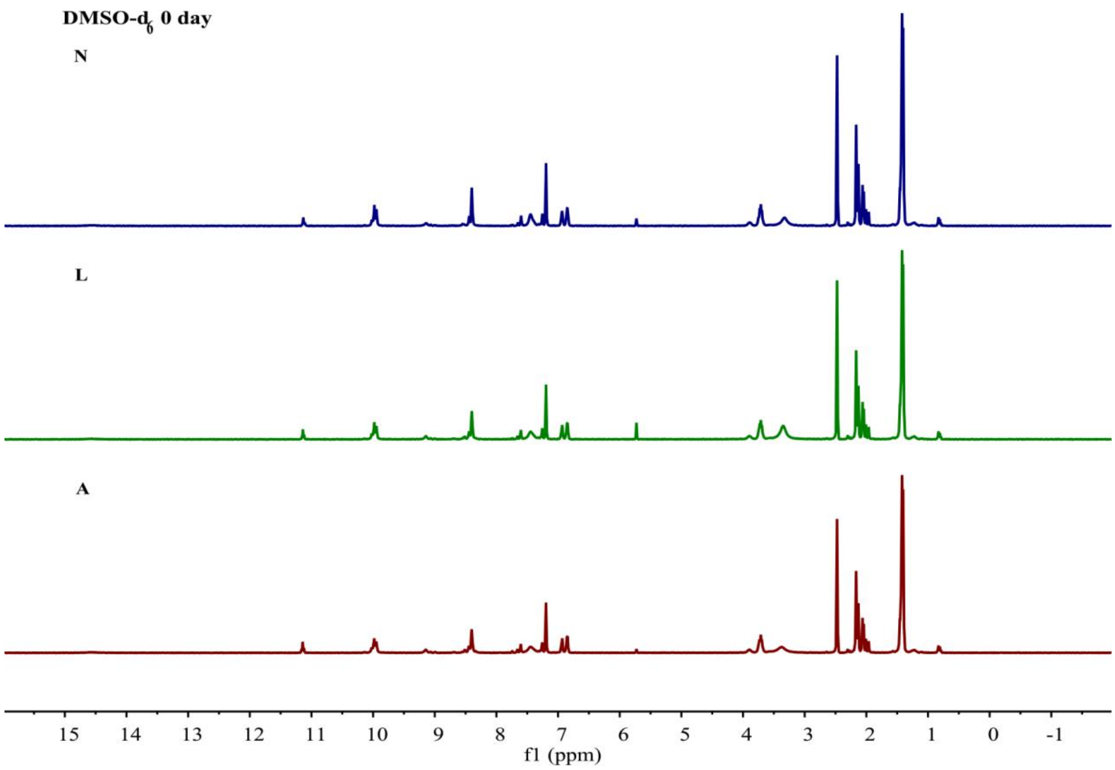

Figure S22. ^{1}H NMR spectrum of the gossypol in DMSO-d_6 solution under different storage conditions in 0 day (N: normal condition without protecting from air and light; L: under natural light and nitrogen protection; A: stored in dark and under nitrogen protection condition).

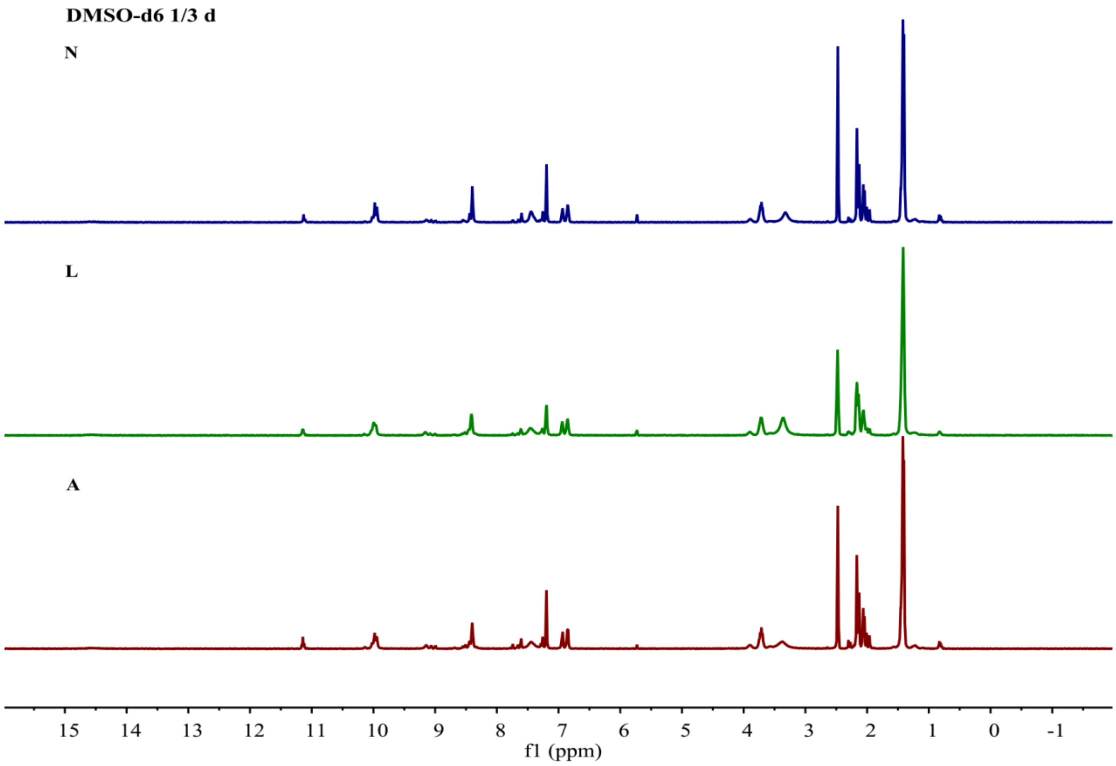

Figure S23. ^{1}H NMR spectrum of the gossypol in DMSO-d_6 solution under different storage conditions in 1/3 days.

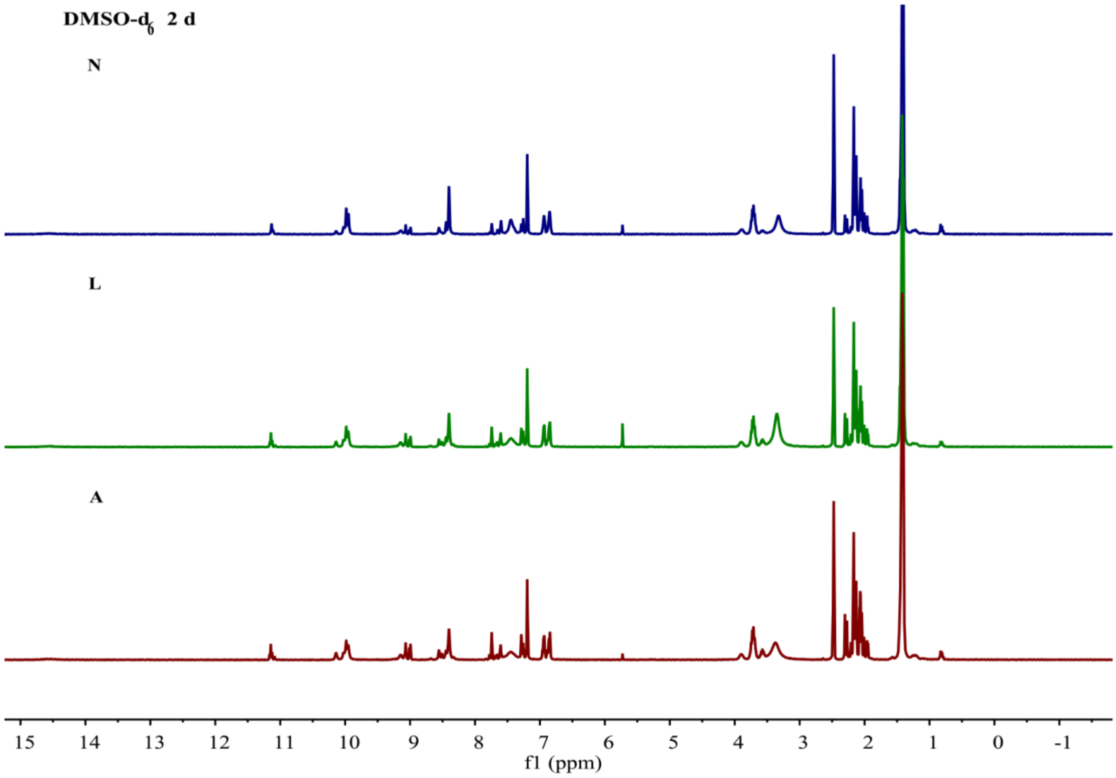

Figure S24. ^{1}H NMR spectrum of the gossypol in DMSO-d6 solution under different storage conditions in 2 days.

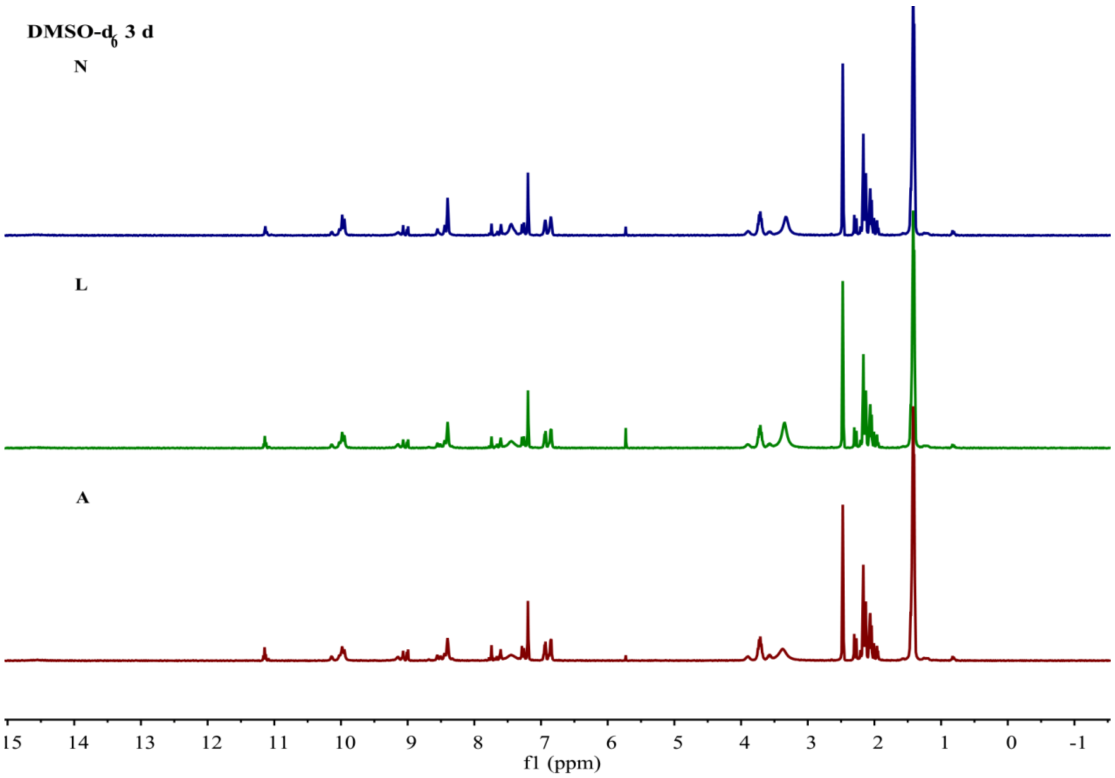

Figure S25. ^{1}H NMR spectrum of the gossypol in DMSO-d6 solution under different storage conditions in 3 days.

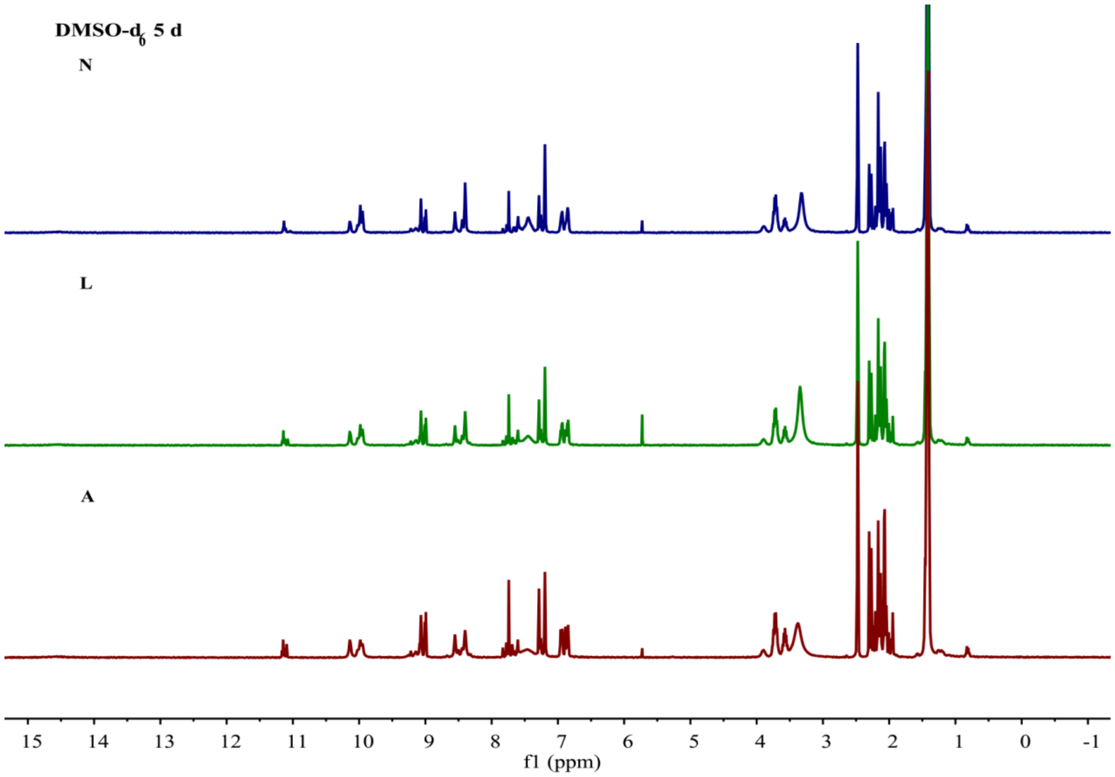

Figure S26. ^{1}H NMR spectrum of the gossypol in DMSO-d6 solution under different storage conditions in 5 days.

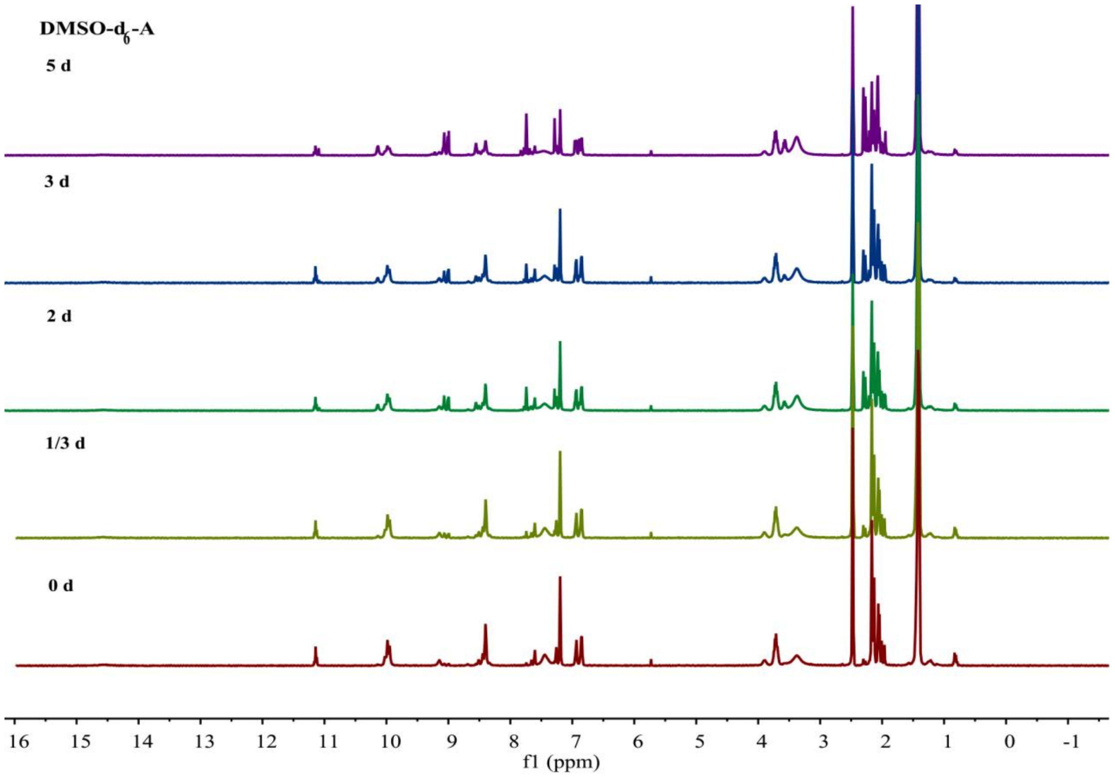

Figure S27. ^{1}H NMR spectrum of the gossypol in DMSO-d6 solution under stored in dark and under nitrogen protection condition storage condition in different days.

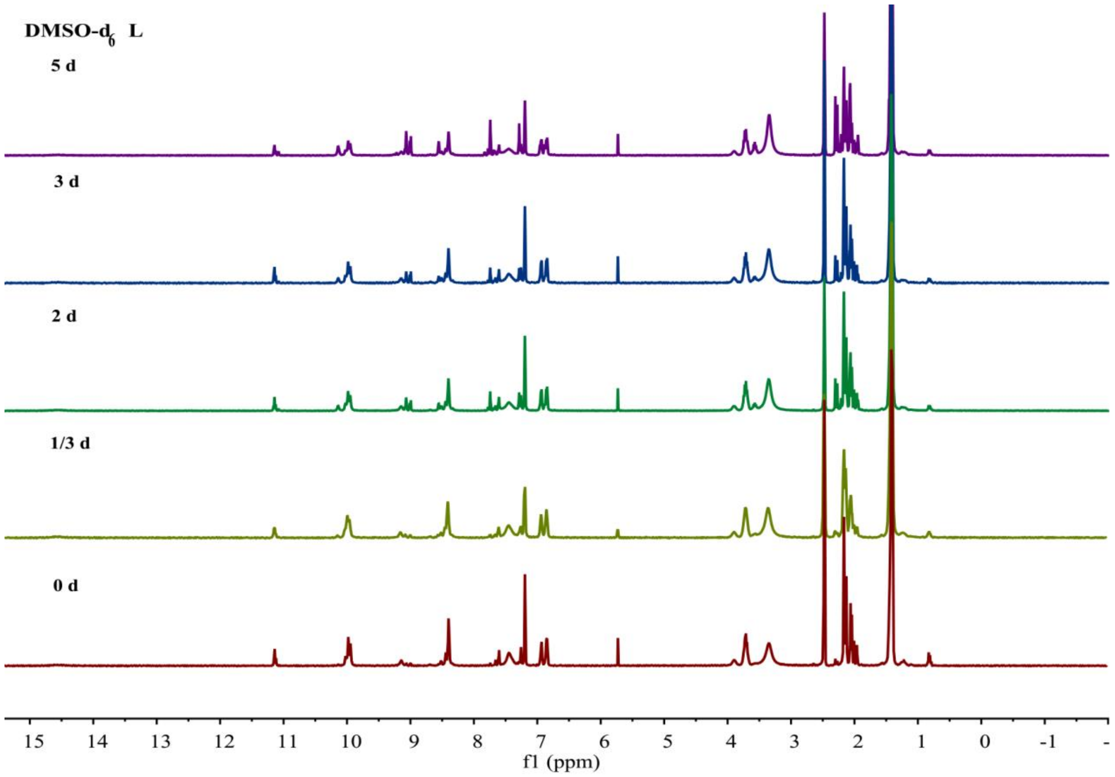

Figure S28.H NMR spectrum of the gossypol in DMSO-d6 solution under natural light and nitrogen protection storage condition in different days.

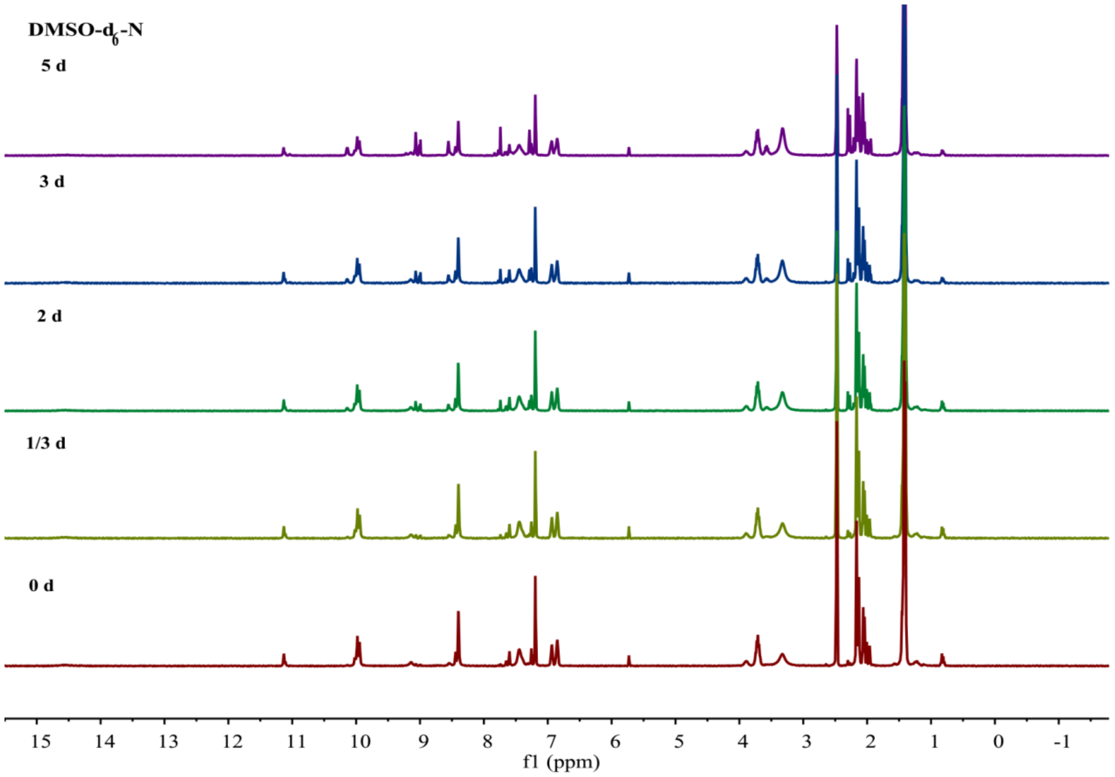

Figure S29. ^{1}H NMR spectrum of the gossypol in DMSO-d6 solution under normal condition without protect from air and light storage condition in different days (0-5 days).

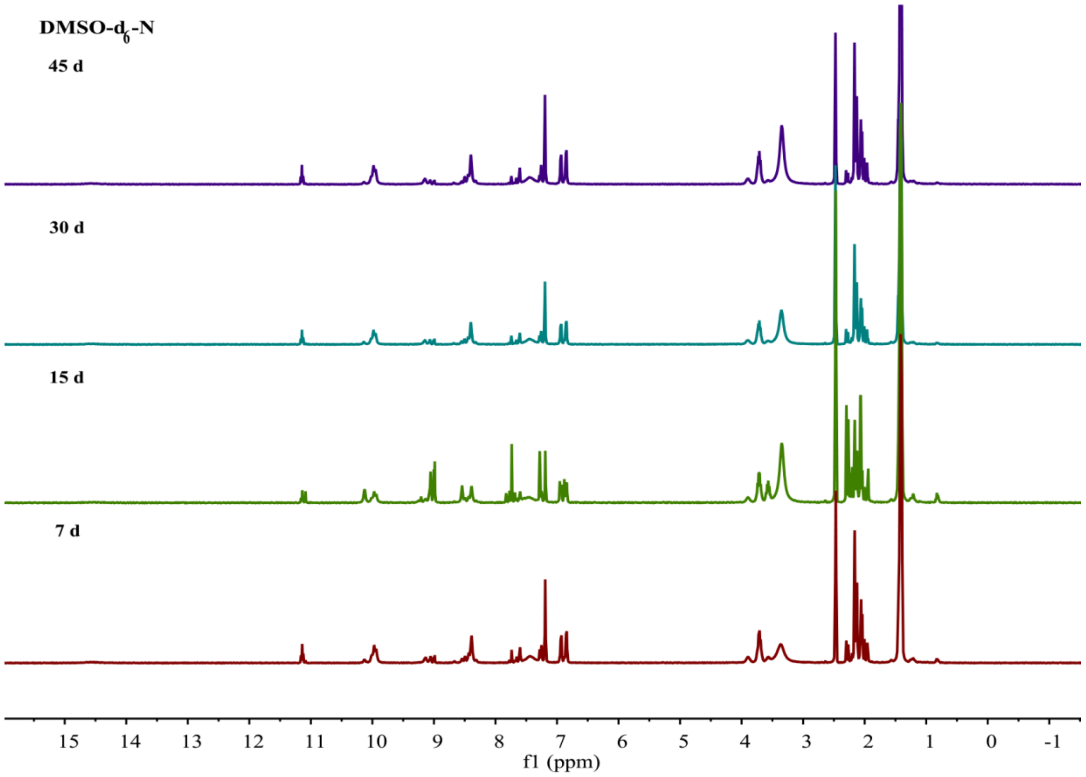

Figure S30. ^{1}H NMR spectrum of the gossypol in DMSO-d_6 solution under normal condition without protect from air and light storage condition in different days (7-45 days).

2. UV-Vis absorbance spectra of Gossypol solutions.

Sample Preparation for UV-Vis absorbance spectra Analysis: 5.0 mg of gossypol was dissolved in 1.0 mL of menthol, chloroform, and Methyl sulfoxide in a screw-capped vial respectively. The samples were stored under normal condition without protecting from light and air for 0 days, 3 days, 5 days, 7days, 15 days, 30 days and 45 days at room temperature and UV-Vis measurements were taken at different time intervals.

UV-Vis measurements were taken with Shimadzu UV-2600 double-beam UV-Vis spectrophotometer equipped with 10-mm quartz absorption cells. The spectrums of the Gossypol solutions samples were recorded in the wavelength range 200-600nm.

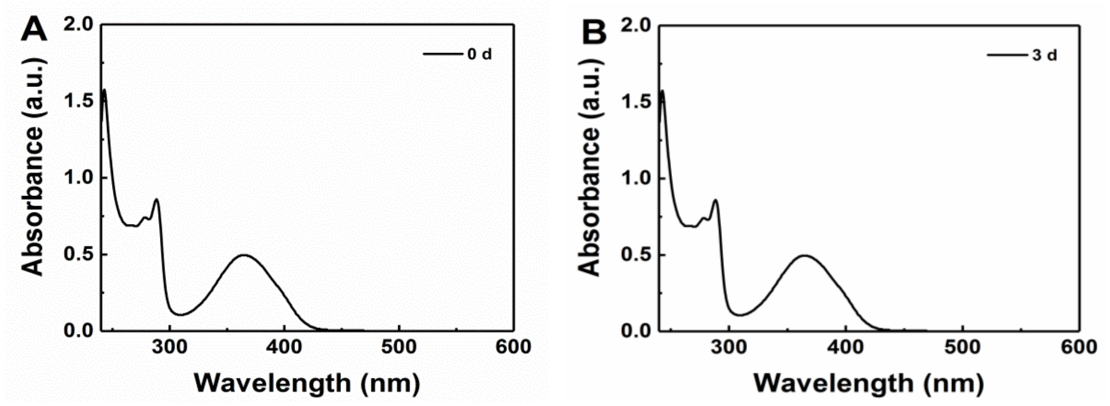

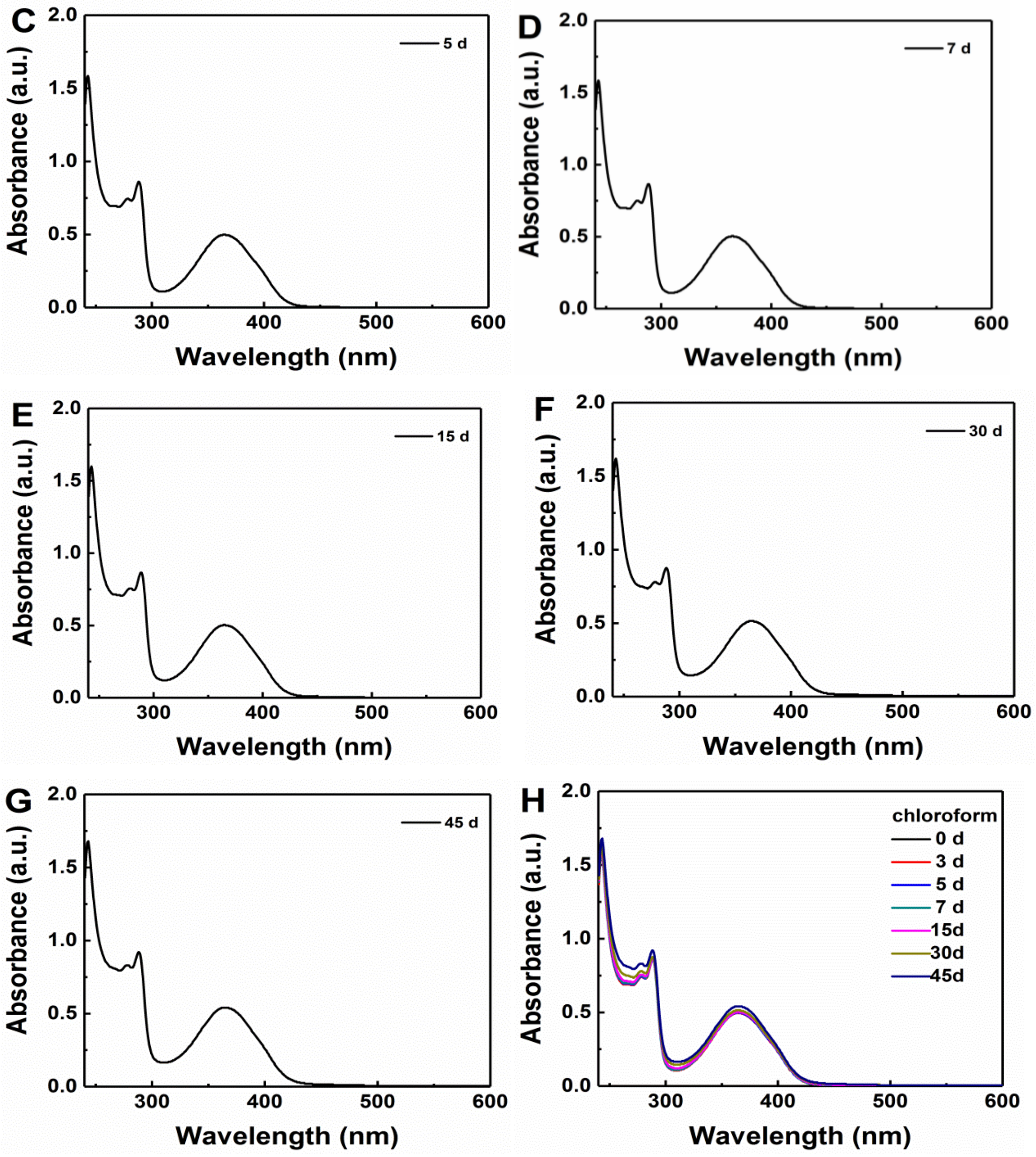

Figure S31. UV-VIS absorbance spectra of gossypol in $CHCl_3$ solvent under normal condition without protect from air and light storage condition (A) for 0 day (B) for 3 days (C) for 5 days (D) for 7 days (E) for 15 days (F) for 30 days (G) for 45 days (H) for different days

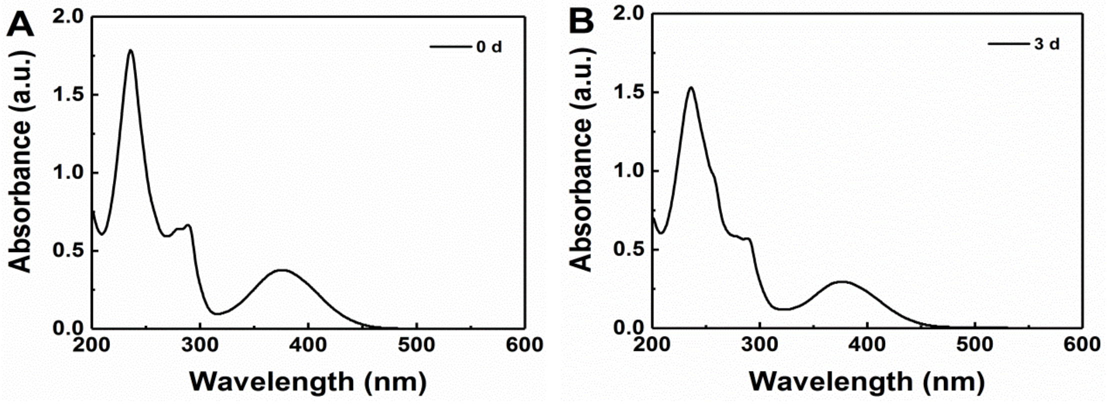

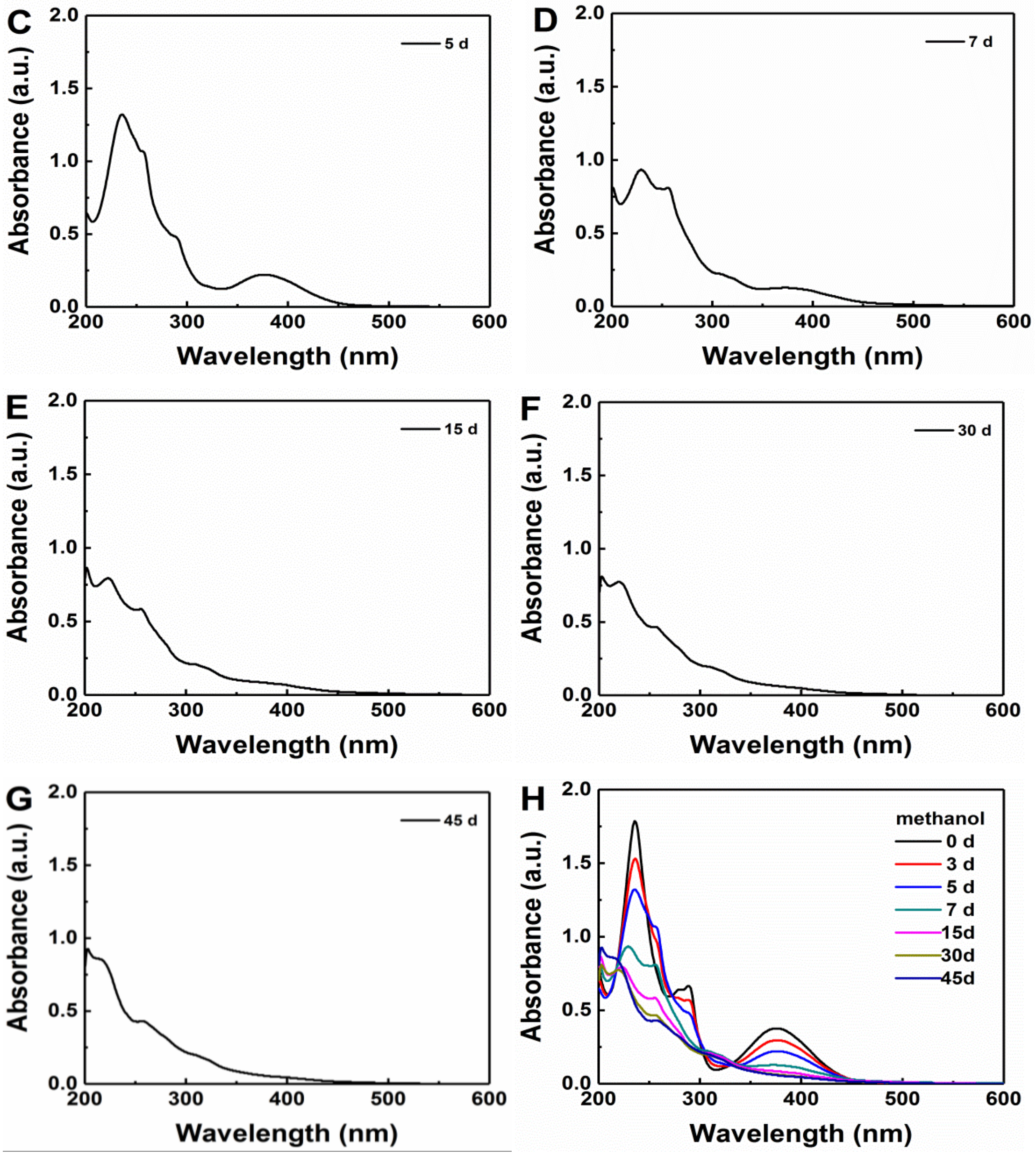

Figure S32. UV-VIS absorbance spectra of gossypol in CH_3OH solvent under normal condition without protect from air and light storage condition (a) for 0 day (b) for 3 days (c) for 5 days (d) for 7 days (e) for 15 days (f) for 30 days (g) for 45 days (h) for different days.

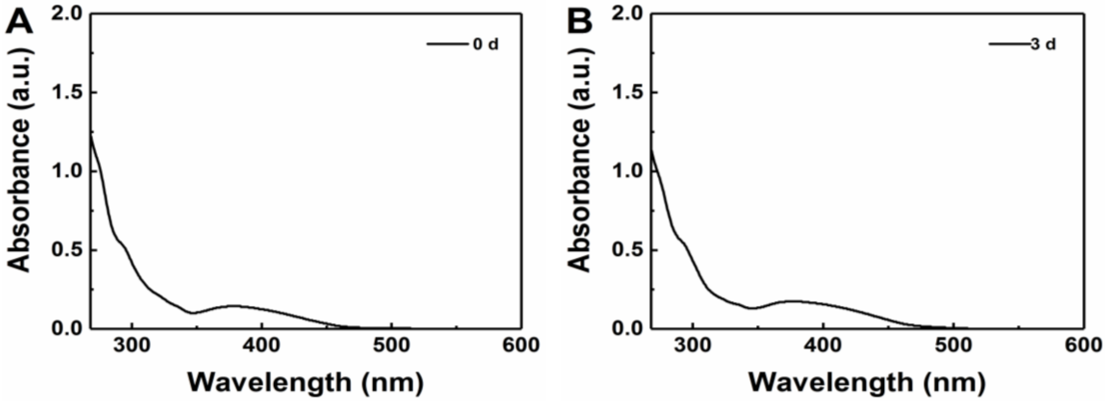

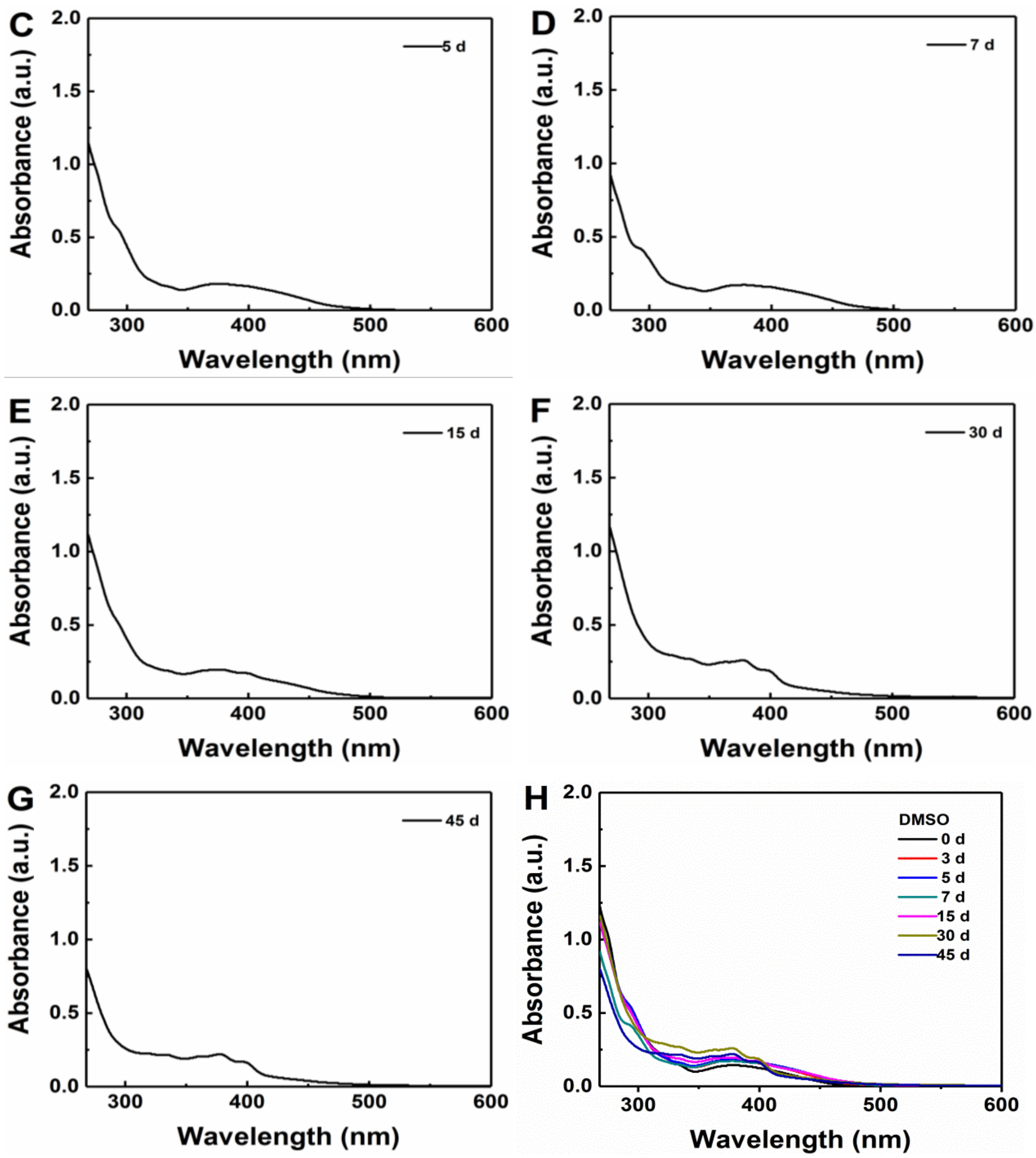

Figure S33. UV-VIS absorbance spectra of gossypol in DMSO solvent under ambient air storage condition (a) for 0 day (b) for 3 days (c) for 5 days (d) for 7 days (e) for 15 days (f) for 30 days (g) for 45 days (h) for different days

3. ATR-FTIR spectra of Gossypol solutions

Sample Preparation for FT-IR spectra Analysis: 5.0 mg of gossypol was dissolved in 1.0 mL of menthol, chloroform, and Methyl sulfoxide in a screw-capped vial respectively. The samples were stored under normal condition without protecting from light and air for 0 days, 3 days, 5 days, 7days, 15 days, 30 days and 45 days at room temperature and gossypol solution was dropwise added into the ATR attachment (ZnSe crystal), then spectra of the gossypol was measurement by ATR-IR after the solution evaporated at room temperature.

Attenuated Total Reflectance Fourier Transform Infrared spectra were obtained with Nicolet 6700 Recording Infrared Spectrophotometer. The temperature of the instrument was maintained at 25±1℃ by water circulating from a constant temperature bath.

The ATR-FTIR spectra of gossypol in chloroform and in methanol over 45 days at room temperature were compared in Figure S34, S35 and S36. The spectra of the gossypol in

chloroform and in methanol were comparable in the region below 2000 cm^{-1} but showed significantly different above this wave number, especially in the region of 3700-3000 cm^{-1}. These signals were due to the ν (OH) vibrations.

The spectra of gossypol in KBr and gossypol in methanol revealed broad bands of ν(OH) vibrations in the region of 3700-3000 cm^{-1}. The spectra of gossypol in KBr showed three bands at 3514, 3421 and 3308 cm^{-1} which were assigned to hydroxyl groups involved in the hydrogen bonds of different strength.

In the spectrum of gossypol in methanol, the band assigned to the ν(OH) stretching vibrations shifted to ca. 3490 cm^{-1} (3496 cm^{-1} for 15 days, 3482 cm^{-1} for 30 days and 45 days) relative to the band at ca.3500 and 3400 cm^{-1} in the spectrum of gossypol for 0 day, 3 days, 5 days and 7 days (3519 and 3393 cm^{-1} for 0 day, 3494 and 3396 cm^{-1} for 3 days, 3675 and 3495 cm^{-1} for 5 days, 3501 and 3419 cm^{-1} for 7 days). The broad band assigned to the stretching vibrations of OH groups at the 7, 7′ positions and to the stretching vibrations of C_{15}-OH and $C_{15'}$-OH groups showed in ca. 3490 cm^{-1}. The lactol-lactol tautomeric structure of gossypol in methanol could be observed in the region.

The signal of the ν(OH) and ν(C=O) stretching vibrations were also compared in Figure S34 and S35(H). The gossypol in KBr showed the vibrations of the strongest $O_{7,7'}$-H hydrogen-bonded proton which was well observed at 3422 cm^{-1}. For gossypol in chloroform, the ν (OH) vibration showed two bands at ca. 3650 cm^{-1} and 3500 cm^{-1}. And the band at ca. 1616 cm^{-1} was assigned to the *ν*(C=O) vibrations. The most important spectral feature was the signal of ν(C=C) vibrations at 1596 and 1507 cm^{-1} which were the characteristic of naphthalene ring in aldehyde–aldehyde tautomeric form of gossypol [1].

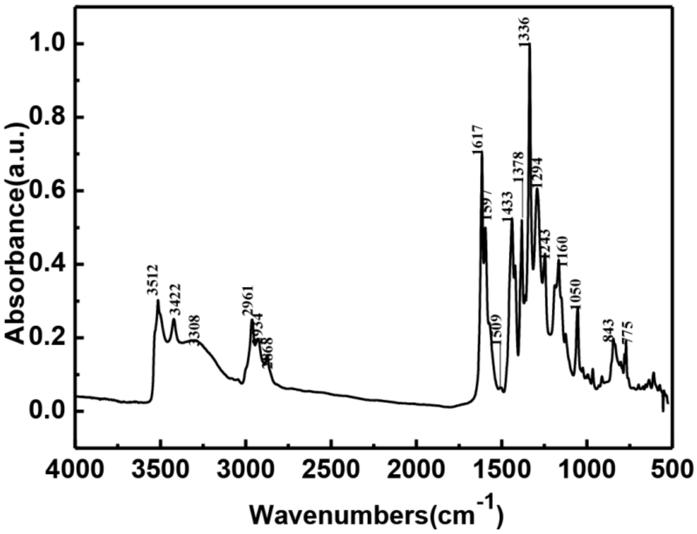

Figure S34. FT-IR spectra of G-KBr solid

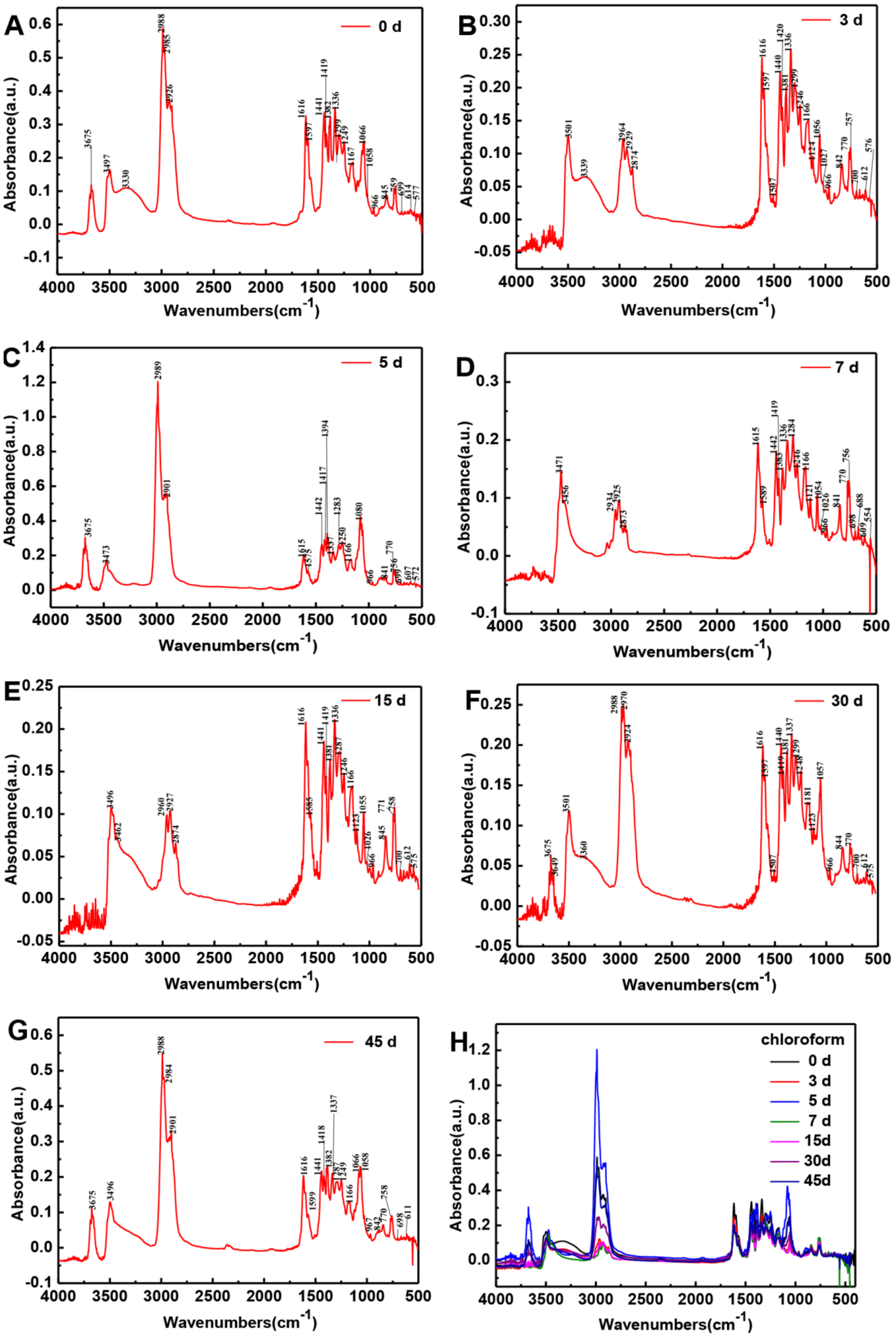

Figure S35. FT-IR spectra of gossypol in $CHCl_3$ solvent under normal condition without protect from air and light storage condition (a) for 0 day (b) for 3 days (c) for 5 days (d) for 7 days (e) for 15 days (f) for 30 days (g) for 45 days (h) for different days.

Table S1 Observed FT-IR wavenumbers and vibrational frequencies $cm^{-1}$are in $CHCl_3$ solvent for different days

G-KBr Wave number(cm-1)	G-$CHCl_3$ Wave number(cm-1)						
	0Day	3Days	5Days	7Days	15Days	30Days	45Days
3514	3675	3501	3676	3472	3496	3675	3675
3422	3498	3339	3474	3456	3462	3649	3497
2963	3330	2964	2990	2934	2960	3502	2989
2928	2989	2929	2901	2925	2927	2988	2984
2874	2985	2874	1617	2873	2874	2971	2901
1616	2927	1617	1575	1615	1616	2925	1617
1596	1616	1597	1443	1589	1507	1617	1599
1440	1597	1441	1418	1443	1585	1597	1441
1421	1441	1420	1394	1419	1441	1507	1418
1382	1419	1381	1337	1383	1420	1441	1383
1336	1383	1337	1283	1337	1381	1419	1337
1293	1337	1299	1251	1285	1420	1420	1287
1246	1300	1247	1166	1246	1337	1382	1250
1188	1250	1167	1080	1166	1288	1337	1167
1166	1167	1124	966	1122	1247	1299	1067
1125	1124	1056	841.3	1055	1167	1248	1058
1055	1067	1026	770.1	1026	1123	1182	966.8
1026	1058	966.3	756.7	966.0	1056	1124	842.5
966.5	966.3	842.9	698.7	841.1	1026	1058	699.8
845.4	845.3	770.7	607.3	770.1	966.4	966.4	758.0
780.3	758.7	757.9	572.6	756.8	844.7	844.5	668.8
771.2	699.0	699.7		698.0	771.0	770.9	611.8
697.8	577.0	612.8		668.7	758.3	612.6	
609.0		576.0		609.0	699.5	575.5	
573.0				553.9	612.8		
					575.6		

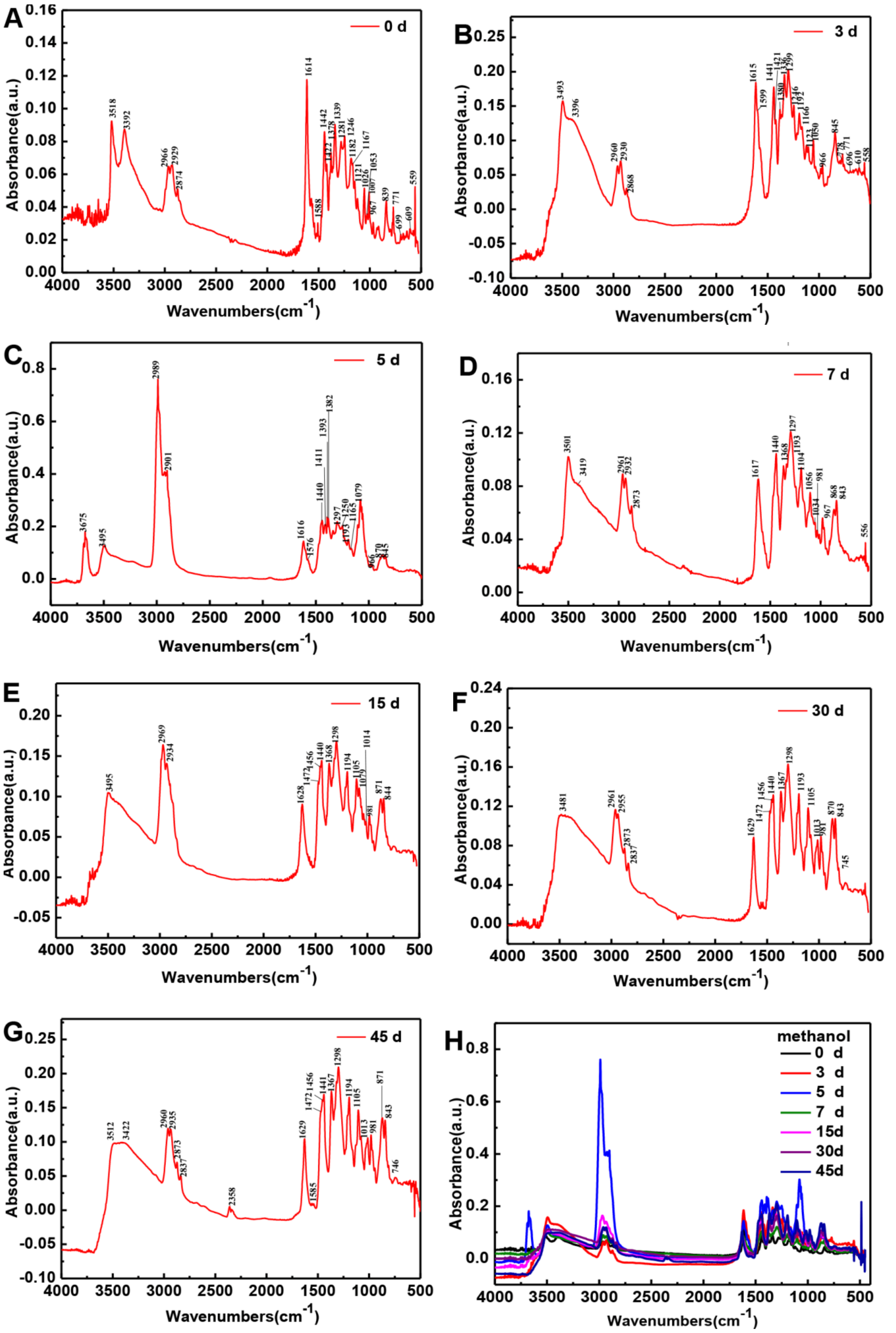

Figure S36. FT-IR spectra of gossypol in CH_3OH solvent under normal condition without protect from air and light storage condition (a) for 0 day (b) for 3 days (c) for 5 days (d) for 7 days (e) for 15 days (f) for 30 days (g) for 45 days (h) for different days.

Table S2 Observed FT-IR wave numbers and vibration frequencies cm^{-1} are in CH_3OH solvent for different days

G-KBr	G-CH_3OH						
Wave number(cm-1)	Wave number(cm-1)						
	0Day	3Days	5Days	7Days	15Days	30Days	45Days
3514	3519	3494	3675	3501	3496	3482	3482
3422	3393	3396	3495	3419	2970	2961	2960
2963	2966	2961	2989	2961	2935	2955	2935
2928	2930	2931	2901	2932	1628	2873	2873
2874	2874	2868	1616	2873	1472	2838	2837
1616	1615	1616	1576	1617	1456	1629	1629
1596	1588	1599	1441	1441	1441	1472	1472
1440	1442	1440	1412	1369	1369	1456	1456
1421	1422	1421	1394	1297	1299	1441	1441
1382	1379	1380	1382	1193	1194	1367	1368
1336	1340	1337	1298	1104	1105	1298	1299
1293	1281	1300	1250	1056	1079	1194	1194
1246	1246	1246	1194	1035	1013	1105	1106
1188	1183	1192	1165	981.4	981.3	1013	1013
1166	1167	1166	1080	967.0	871.4	981.2	981.4
1125	1121	1123	981.2	868.6	844.0	870.3	871.7
1055	1054	1055	966.9	843.2		843.2	843.9
1026	1026	966.9	870.1	556.7			746.4
966.5	1008	845.1	845.1				
845.4	966.8	778.0					
780.3	839.5	771.8					
771.2	771.0	696.2					
697.8	699.0	610.0					
609.0	577.0	558.5					
573.0	559.1						

4. HPLC-QTOF-MS spectrum of Gossypol solutions

Sample Preparation for LC-MS spectra Analysis**:** 5.0 mg of gossypol was dissolved in 1.0 mL of menthol, chloroform, and Methyl sulfoxide in a screw-capped vial respectively. The samples were stored under normal condition without protecting from light and air for 0 days, 3 days, 5 days, 7days, 15 days, 30 days and 45 days at room temperature and LC-MS measurements were taken at different time intervals.

The LC-MS analyses were performed on an Agilent liquid chromatography coupled to a 4000 QST Elite mass spectrometer, equipped with electro spray ionization (ESI) source and a quadrupole-time of flight-mass spectrometry. 5μL of the diluted gossypol solutions was injected onto a Phenomenex column(250×4.60mm, Synergi 4u Hydro-RP 80A) at 30°Cwith a flow rate of 300 μL/min, Mobile phase ACN (A) and 0.1%(v/v) formic acid in water (B) were used for chromatographic separation. Gossypol were eluted with a gradient of 50%A and 50% B (v/v)from 0-8min; 100% A from 8-20min. Mass detection was carried out after electro spray ionization (ESI) in negative -ion full scan mode. The settings of the mass spectrometer were as

follows: spray voltage, 4.5 kV; Source Temperature, 450℃; maxium injection time, 250 ms; scan rage, m/z 100 to1500; Nitrogen was used as sheath gas (pressure 40psi) and auxiliary gas (pressure 60 psi). The in-source collision induced dissociation energy (CID) was set at 50 eV.

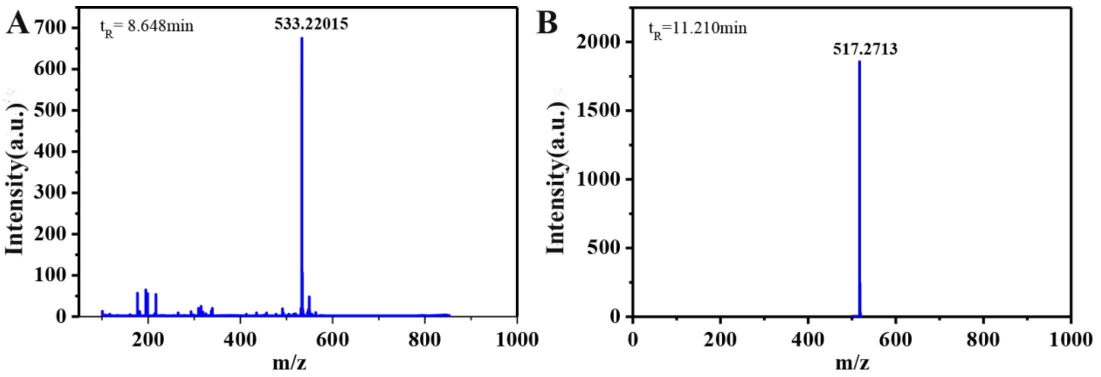

Figure S37. LC-MS chromatograms of gossypol in $CHCl_3$ solution normal condition without protect from air and light storage condition in 0 day. (A) Mass spectrum acquired in retention time (8.65min) in correspond chromatogram. (B) Mass spectrum acquired in retention time (11.21min) in correspond chromatogram.

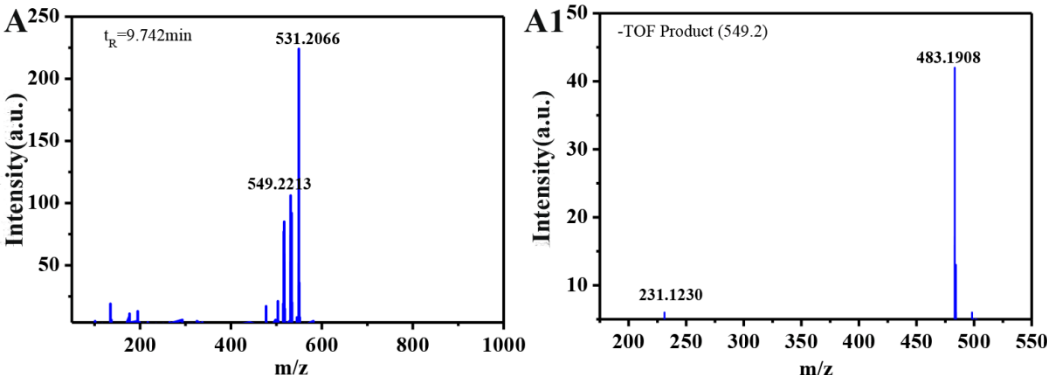

Figure S38. LC-MS chromatograms of gossypol in $CHCl_3$ solution normal condition without protect from air and light storage condition in 3 days. (A) Mass spectrum acquired in retention time (9.74min) in correspond chromatogram. (A1) Fragment of molecular ion at m/z 549.2.

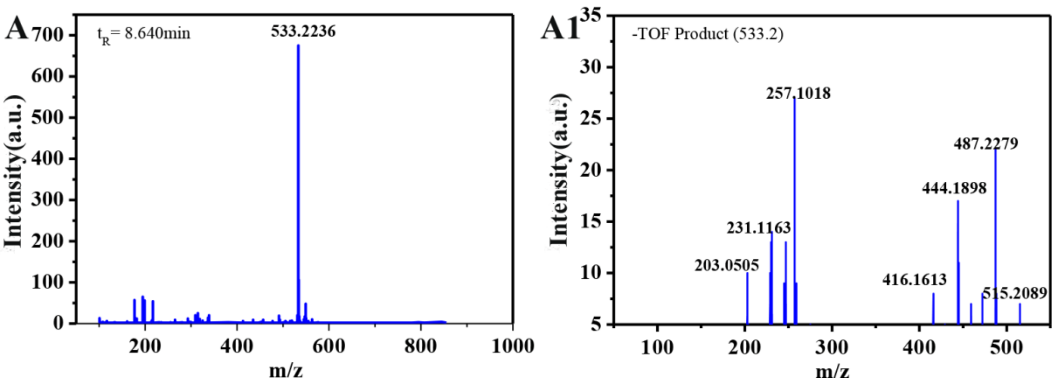

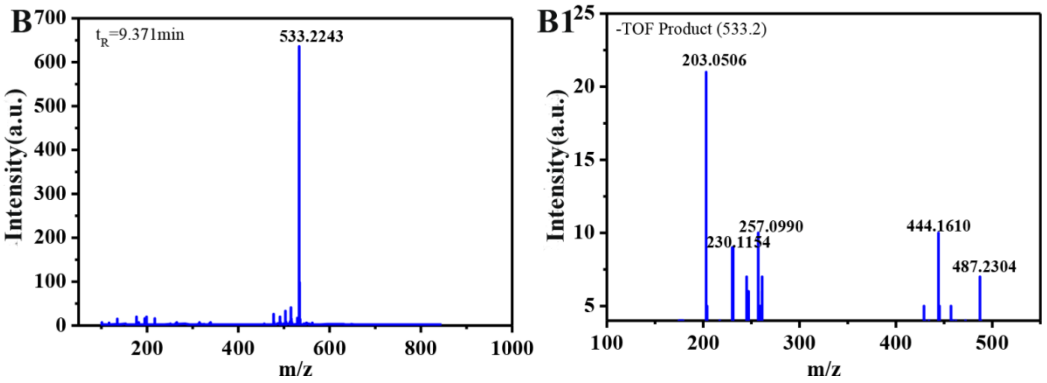

Figure S39. LC-MS chromatograms of gossypol in $CHCl_3$ solution normal condition without protect from air and light storage condition in 3 days. (A) Mass spectrum acquired in retention time (8.64min) in correspond chromatogram. (A1) Fragment of molecular ion at m/z 533.2. (B) Mass spectrum acquired in retention time (9.37min) in correspond chromatogram. (B1) Fragment of molecular ion at m/z 533.2.

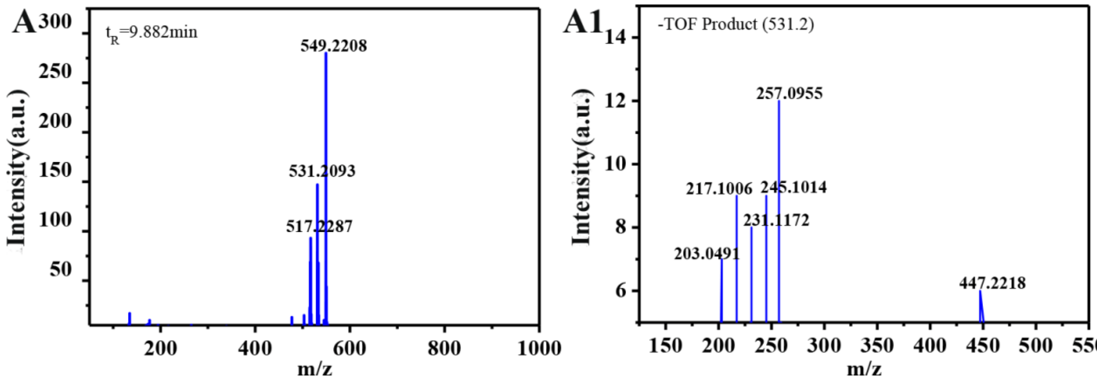

Figure S40. LC-MS chromatograms of gossypol in $CHCl_3$ solution normal condition without protect from air and light storage condition in 3 days. (A) Mass spectrum acquired in retention time (9.88min) in correspond chromatogram. (A1) Fragment of molecular ion at m/z 531.2.

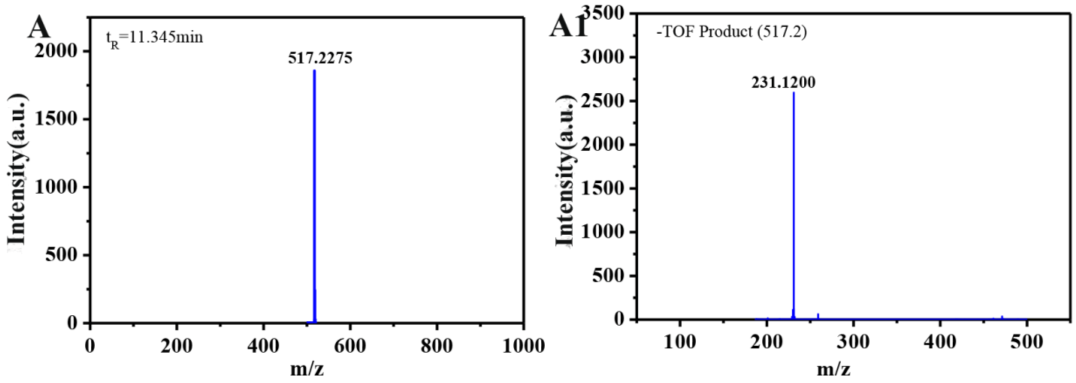

Figure S41. LC-MS chromatograms of gossypol in $CHCl_3$ solution normal condition without protect from air and light storage condition in 3 days. (A) Mass spectrum acquired in retention time (11.35min) in correspond chromatogram. (A1) Fragment of molecular ion at m/z 517.2.

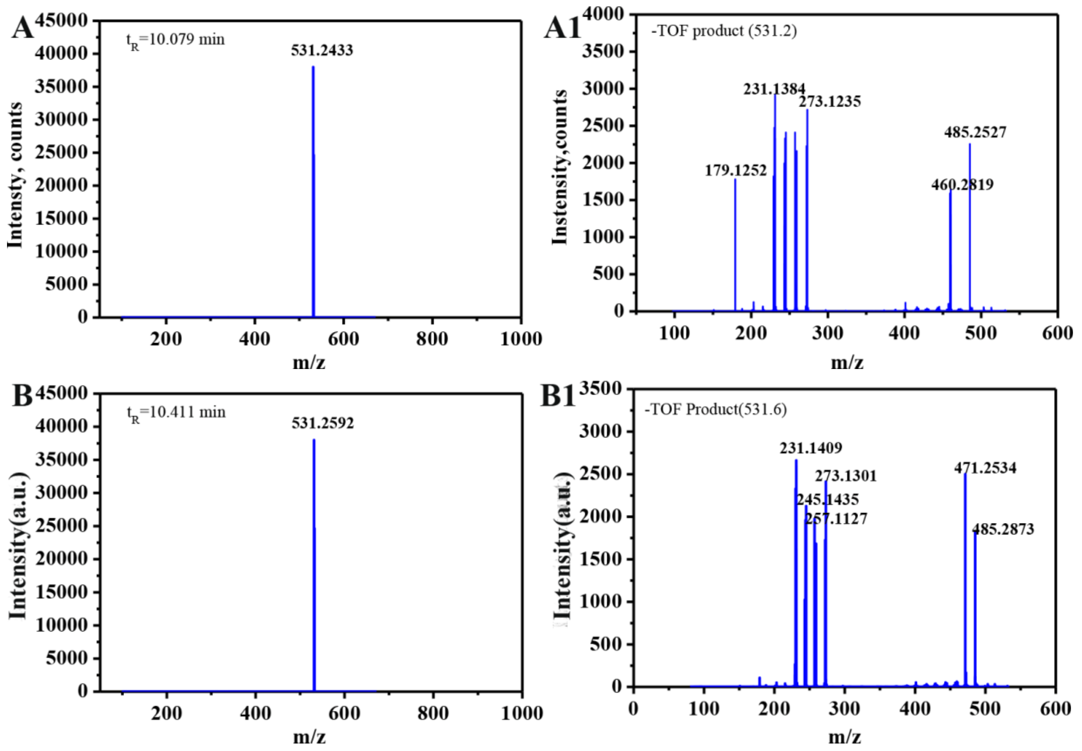

Figure S42. LC-MS chromatograms of gossypol in $CHCl_3$ solution normal condition without protect from air and light storage condition in 5 days. (A) Mass spectrum acquired in retention time (10.08min) in correspond chromatogram. (A1) Fragment of molecular ion at m/z 531.2. (B) Mass spectrum acquired in retention time (10.411min) in correspond chromatogram. (B1) Fragment of molecular ion at m/z 531.6.

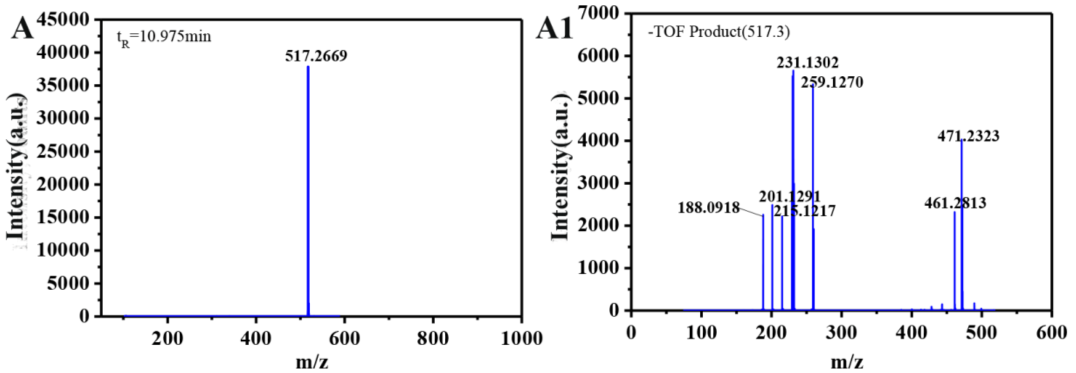

Figure S43. LC-MS chromatograms of gossypol in $CHCl_3$ solution normal condition without protect from air and light storage condition in 5 days. (A) Mass spectrum acquired in retention time (10.98min) in correspond chromatogram. (A1) Fragment of molecular ion at m/z 517.3.

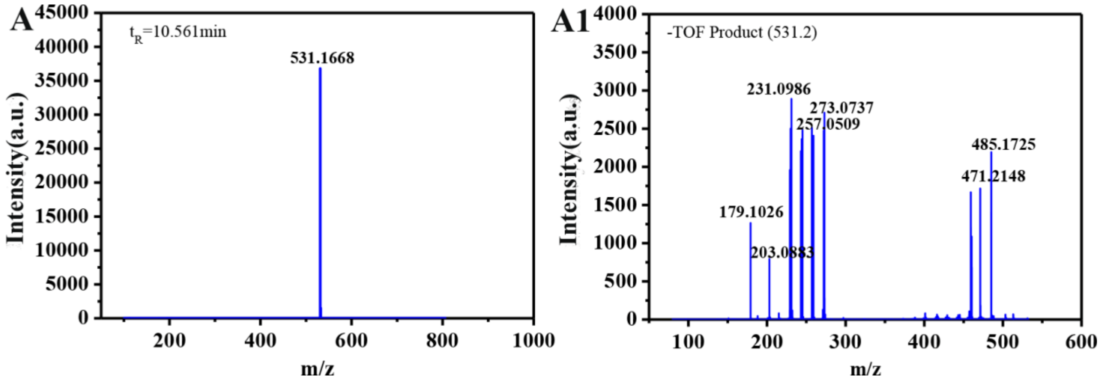

Figure S44. LC-MS chromatograms of gossypol in $CHCl_3$ solution normal condition without protect from air and light storage condition in 7 days. (A) Mass spectrum acquired in retention time (10.56min) in correspond chromatogram. (A1) Fragment of molecular ion at m/z 531.2.

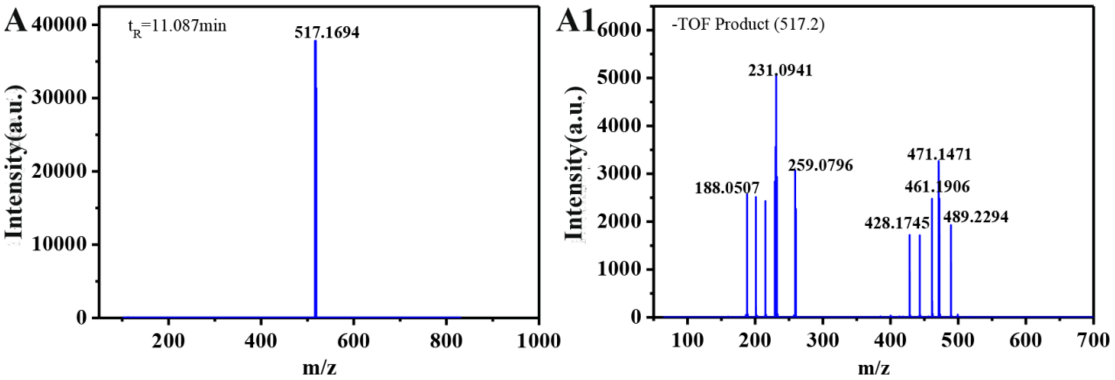

Figure S45. LC-MS chromatograms of gossypol in $CHCl_3$ solution normal condition without protect from air and light storage condition in 7 days. (A) Mass spectrum acquired in retention time (11.48min) in correspond chromatogram. (A1) Fragment of molecular ion at m/z 517.2.

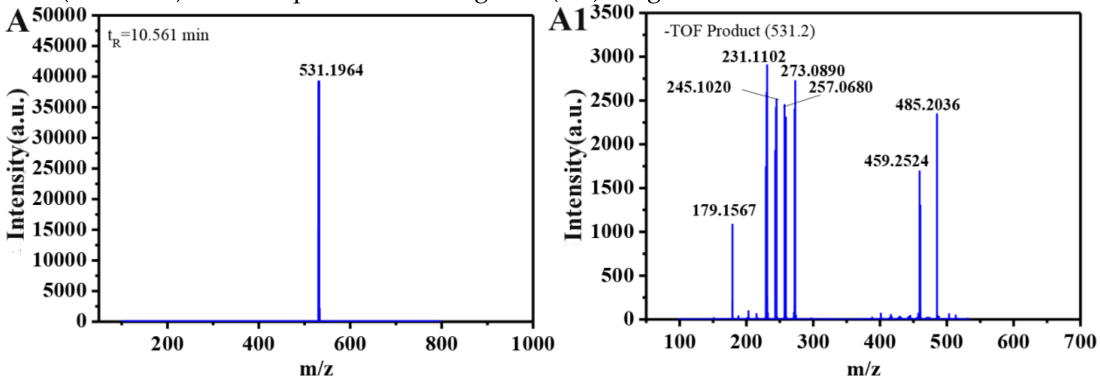

Figure S46. LC-MS chromatograms of gossypol in $CHCl_3$ solution normal condition without protect from air and light storage condition in 15 days. (A) Mass spectrum acquired in retention time (10.56min) in correspond chromatogram. (A1) Fragment of molecular ion at m/z 531.2.

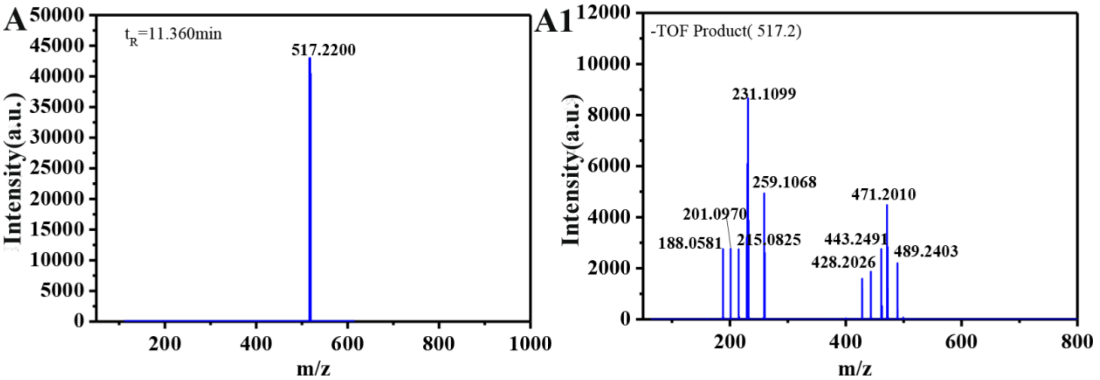

Figure S47. LC-MS chromatograms of gossypol in $CHCl_3$ solution normal condition without protect from air and light storage condition in 15 days. (A) Mass spectrum acquired in retention time (11.36min) in correspond chromatogram. (A1) Fragment of molecular ion at m/z 517.2.

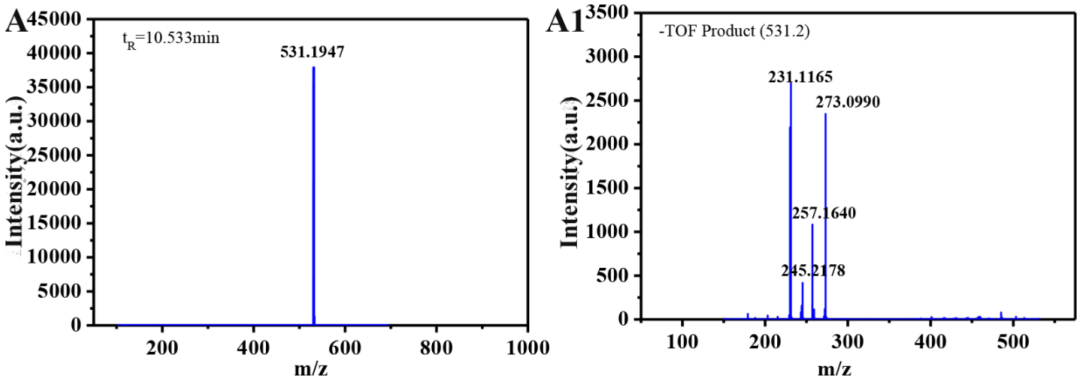

Figure S48. LC-MS chromatograms of gossypol in $CHCl_3$ solution normal condition without protect from air and light storage condition in30 days. (A) Mass spectrum acquired in retention time (10.53min) in correspond chromatogram. (A1) Fragment of molecular ion at m/z 531.2.

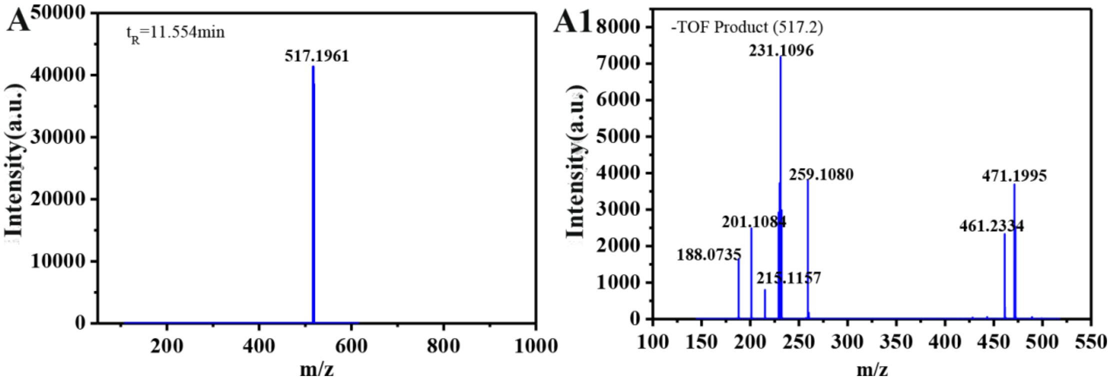

Figure S49. LC-MS chromatograms of gossypol in $CHCl_3$ solution normal condition without protect from air and light storage condition in 30 days. (A) Mass spectrum acquired in retention time (11.54min) in correspond chromatogram. (A1) Fragment of molecular ion at m/z 517.2.

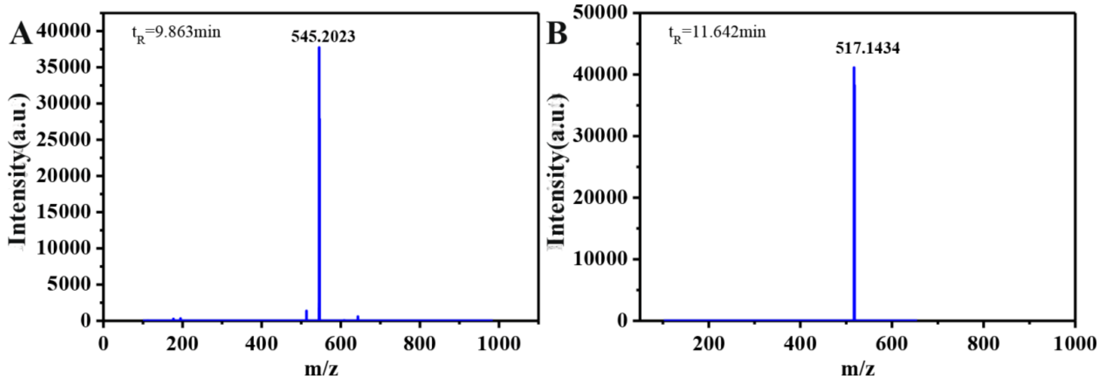

Figure S50. LC-MS chromatograms of gossypol in CH_3OH solution normal condition without protect from air and light storage condition in 0 day. (A) Mass spectrum acquired in retention time (9.86min) in correspond chromatogram. (B) Mass spectrum acquired in retention time (11.64min) in correspond chromatogram.

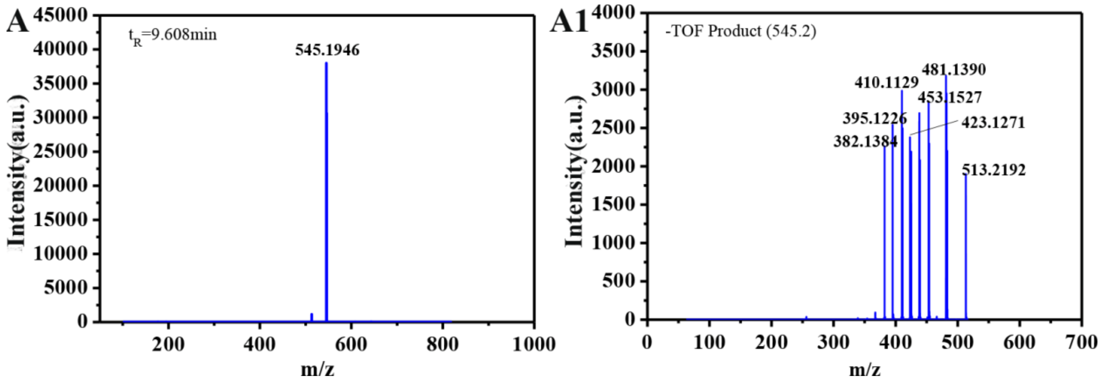

Figure S51. LC-MS chromatograms of gossypol in CH_3OH solution normal condition without protect from air and light storage condition in 3 days. (A) Mass spectrum acquired in retention time (9.61min) in correspond chromatogram. (A1) Fragment of molecular ion at m/z 545.2.

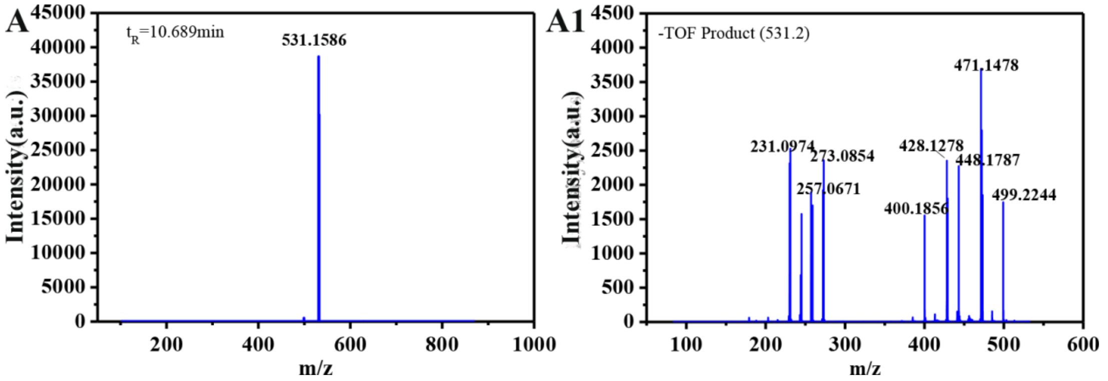

Figure S52. LC-MS chromatograms of gossypol in CH_3OH solution normal condition without protect from air and light storage condition in 3 days. (A) Mass spectrum acquired in retention time (10.69min) in correspond chromatogram. (A1) Fragment of molecular ion at m/z 531.2.

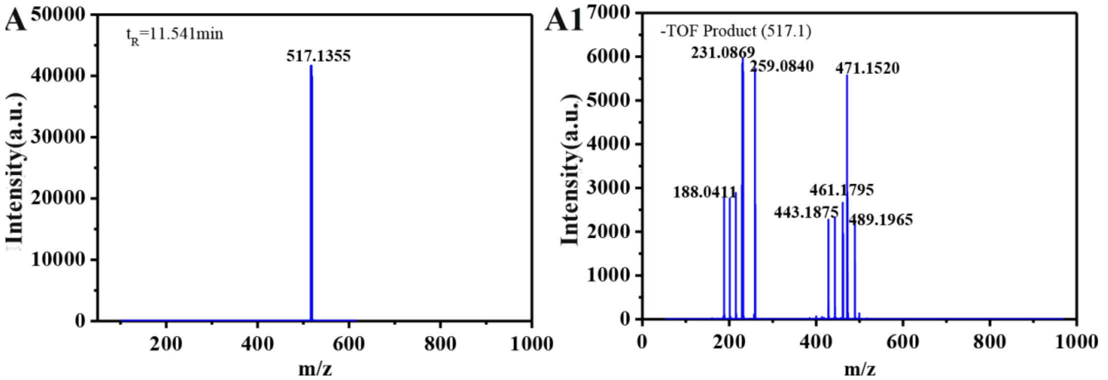

Figure S53. LC-MS chromatograms of gossypol in CH_3OH solution normal condition without protect from air and light storage condition in 3 days. (A) Mass spectrum acquired in retention time (11.54min) in correspond chromatogram. (A1) Fragment of molecular ion at m/z 517.1

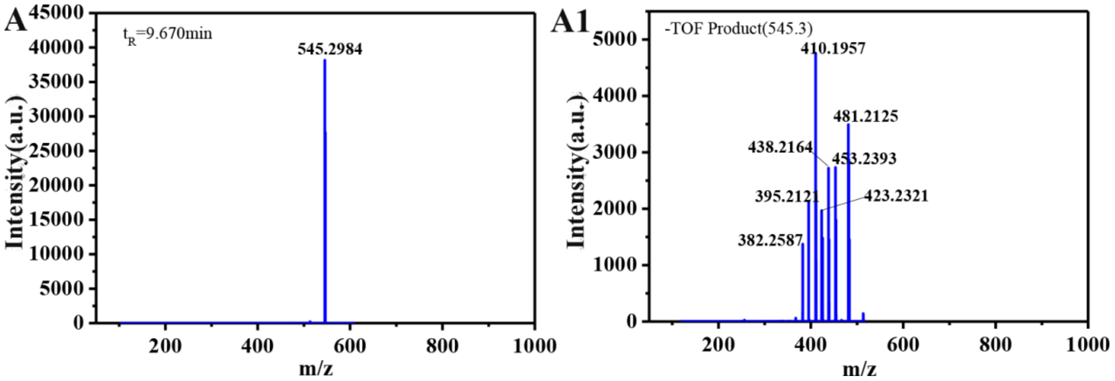

Figure S54. LC-MS chromatograms of gossypol in CH_3OH solution normal condition without protect from air and light storage condition in 5 days. (A) Mass spectrum acquired in retention time (9.67min) in correspond chromatogram. (A1) Fragment of molecular ion at m/z 545.3.

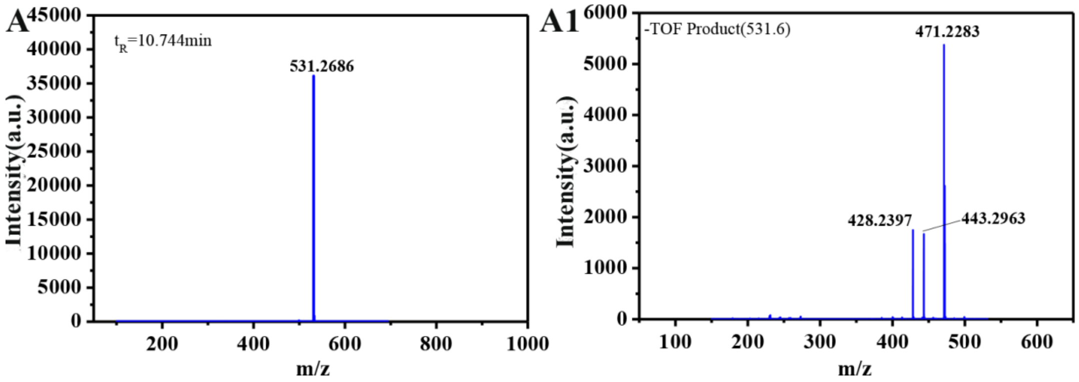

Figure S55. LC-MS chromatograms of gossypol in CH_3OH solution normal condition without protect from air and light storage condition in 5 days. (A) Mass spectrum acquired in retention time (10.74min) in correspond chromatogram. (A1) Fragment of molecular ion at m/z 531.6.

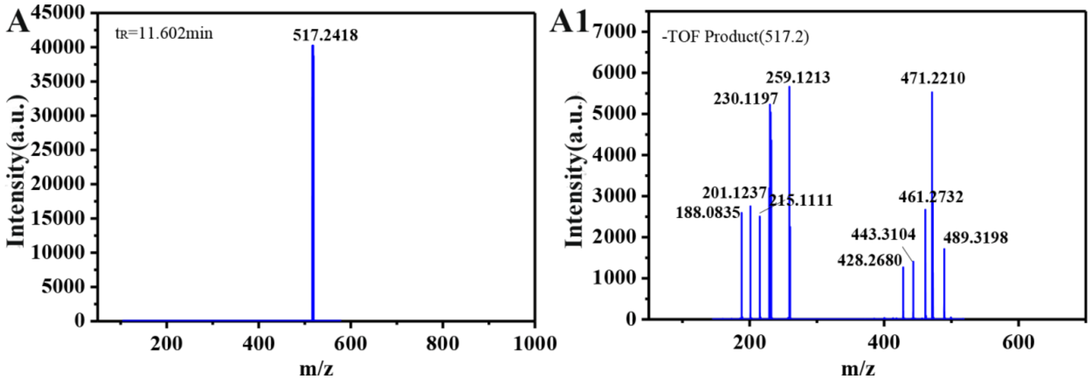

Figure S56. LC-MS chromatograms of gossypol in CH_3OH solution normal condition without protect from air and light storage condition in 5 days. (A) Mass spectrum acquired in retention time (11.60min) in correspond chromatogram. (A1) Fragment of molecular ion at m/z 517.2.

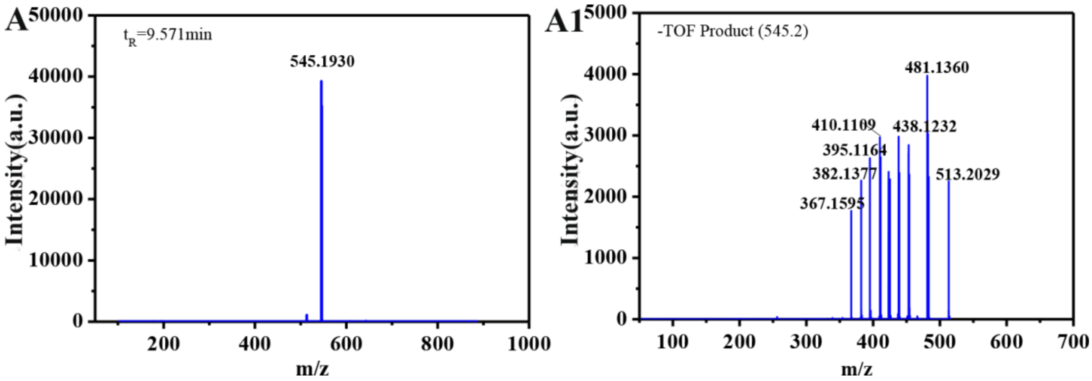

Figure S57. LC-MS chromatograms of gossypol in CH_3OH solution normal condition without protect from air and light storage condition in 7 days. (A) Mass spectrum acquired in retention time (9.57min) in correspond chromatogram. (A1) Fragment of molecular ion at m/z 545.2.

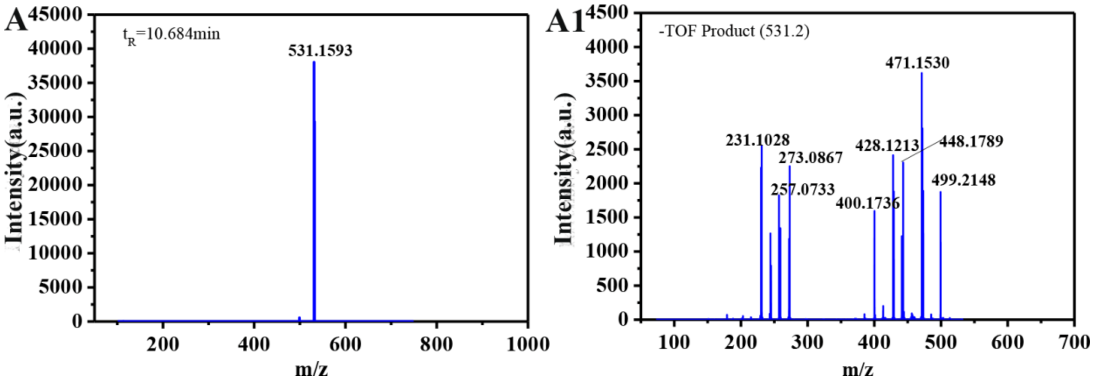

Figure S58. LC-MS chromatograms of gossypol in CH_3OH solution normal condition without protect from air and light storage condition in 7 days. (A) Mass spectrum acquired in retention time (10.68min) in correspond chromatogram. (A1) Fragment of molecular ion at m/z 531.2.

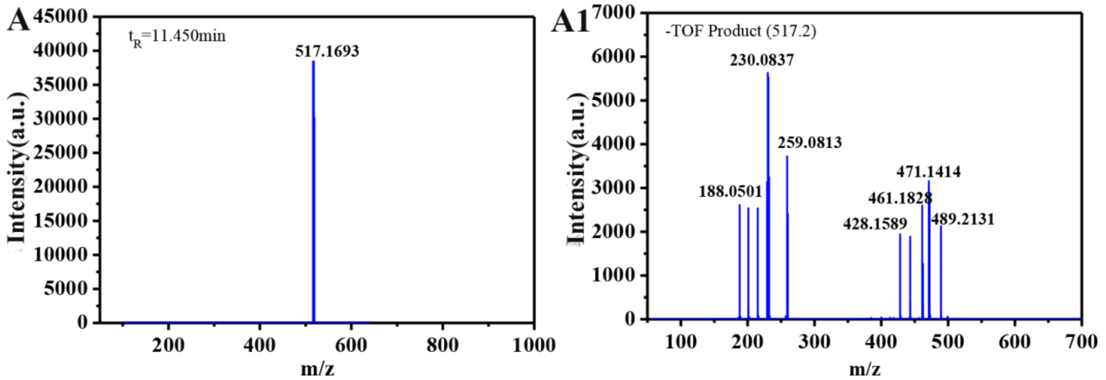

Figure S59. LC-MS chromatograms of gossypol in CH_3OH solution normal condition without protect from air and light storage condition in 7 days. (A) Mass spectrum acquired in retention time (11.45min) in correspond chromatogram. (A1) Fragment of molecular ion at m/z 517.2.

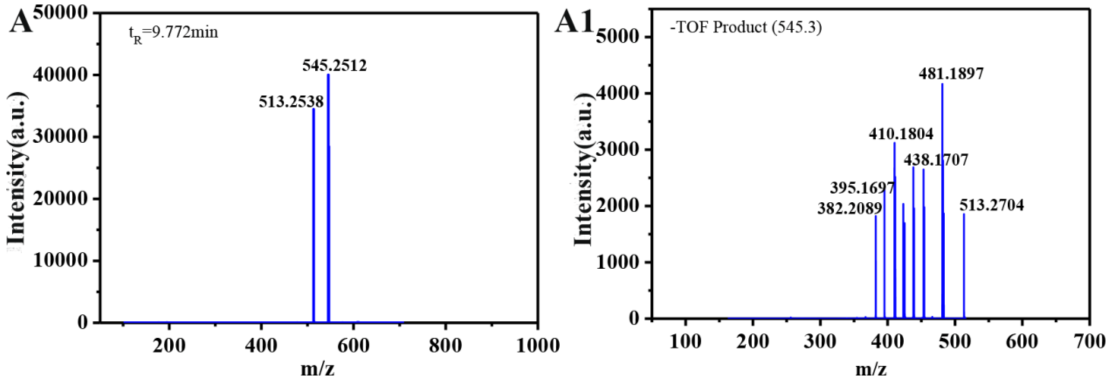

Figure S60. LC-MS chromatograms of gossypol in CH_3OH solution normal condition without protect from air and light storage condition in 15 days. (A) Mass spectrum acquired in retention time (9.77min) in correspond chromatogram. (A1) Fragment of molecular ion at m/z 545.3.

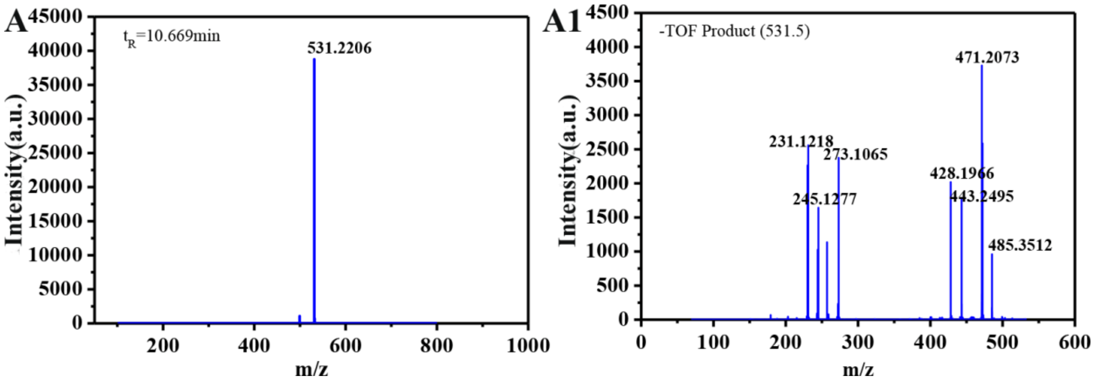

Figure S61. LC-MS chromatograms of gossypol in CH_3OH solution normal condition without protect from air and light storage condition in 15 days. (A) Mass spectrum acquired in retention time (10.67min) in correspond chromatogram. (A1) Fragment of molecular ion at m/z 531.5.

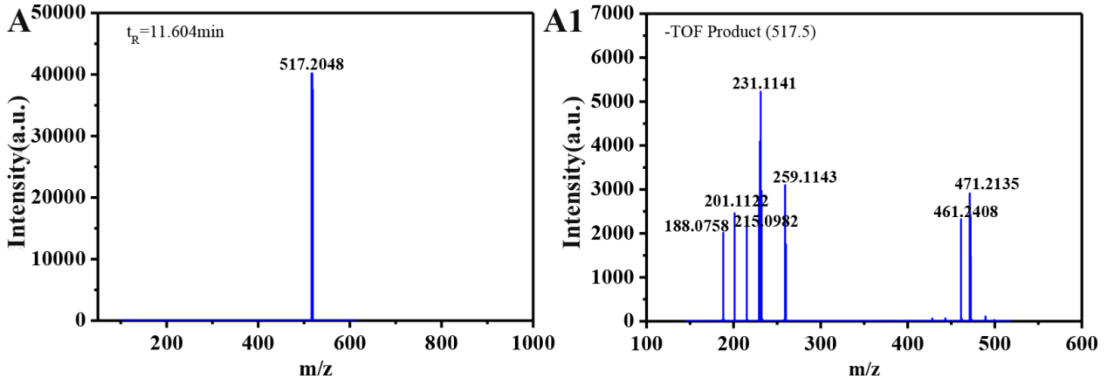

Figure S62. LC-MS chromatograms of gossypol in CH_3OH solution normal condition without protect from air and light storage condition in 15 days. (A) Mass spectrum acquired in retention time (11.60min) in correspond chromatogram. (A1) Fragment of molecular ion at m/z 517.2.

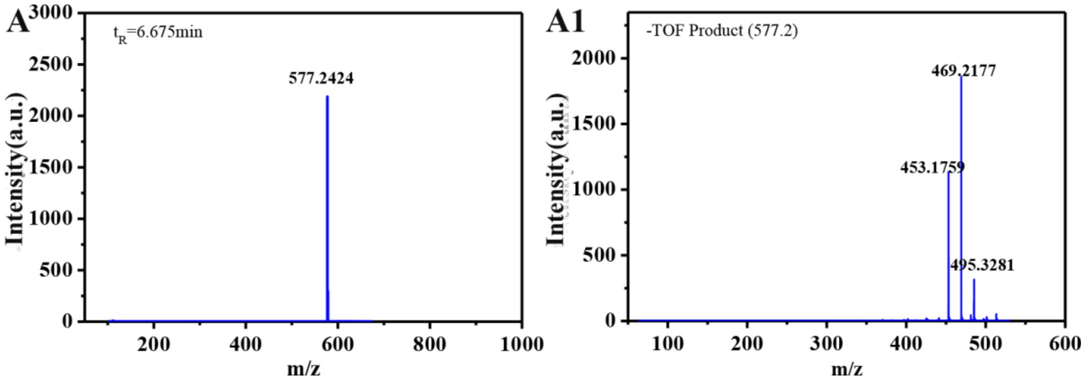

Figure S63. LC-MS chromatograms of gossypol in CH_3OH solution normal condition without protect from air and light storage condition in 30 days. (A) Mass spectrum acquired in retention time (6.67min) in correspond chromatogram. (A1) Fragment of molecular ion at m/z 577.2

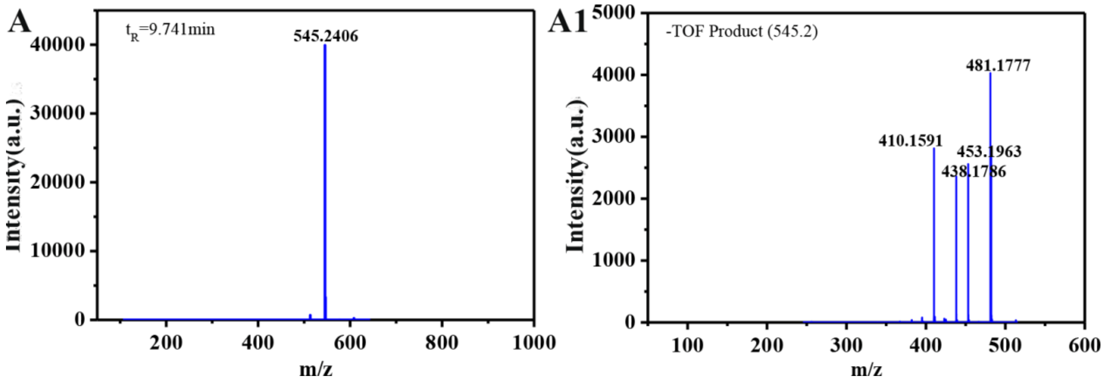

Figure S64. LC-MS chromatograms of gossypol in CH_3OH solution normal condition without protect from air and light storage condition in 30 days. (A) Mass spectrum acquired in retention time (9.74min) in correspond chromatogram. (A1) Fragment of molecular ion at m/z 545.2.

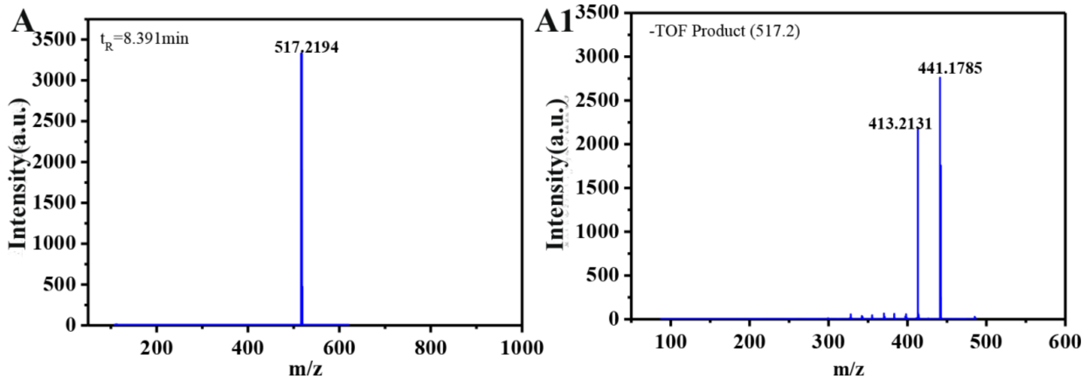

Figure S65. LC-MS chromatograms of gossypol in CH_3OH solution normal condition without protect from air and light storage condition in 30 days. (A) Mass spectrum acquired in retention time (8.4min) in correspond chromatogram. (A1) Fragment of molecular ion at m/z 517.2.

Table S3 Diff of main fragmentation of gossypol molecular ion

Formula	Calculated Mass (m/z)	Measured Mass (m/z)	Diff (ppm)
$C_{30}H_{29}O_8$	517.1862	517.1864	0.303024
$C_{31}H_{31}O_8$	531.2019	531.2014	-0.92876
$C_{30}H_{29}O_9$	533.1812	533.1838	4.955289
$C_{14}H_{15}O_3$	231.1021	231.1017	-1.81531
$C_{32}H_{33}O_8$	545.2175	545.2172	-0.07968
$C_{29}H_{27}O_6$	471.1808	471.1852	9.414727
$C_{29}H_{29}O_7$	489.1913	489.1924	2.190059
$C_{30}H_{27}O_7$	499.1757	499.1743	-2.76168
$C_{29}H_{25}O_5$	453.1702	453.1711	1.98777
$C_{30}H_{25}O_6$	481.1651	481.1641	-2.10706
$C_{31}H_{29}O_7$	513.1913	513.1904	-1.80954

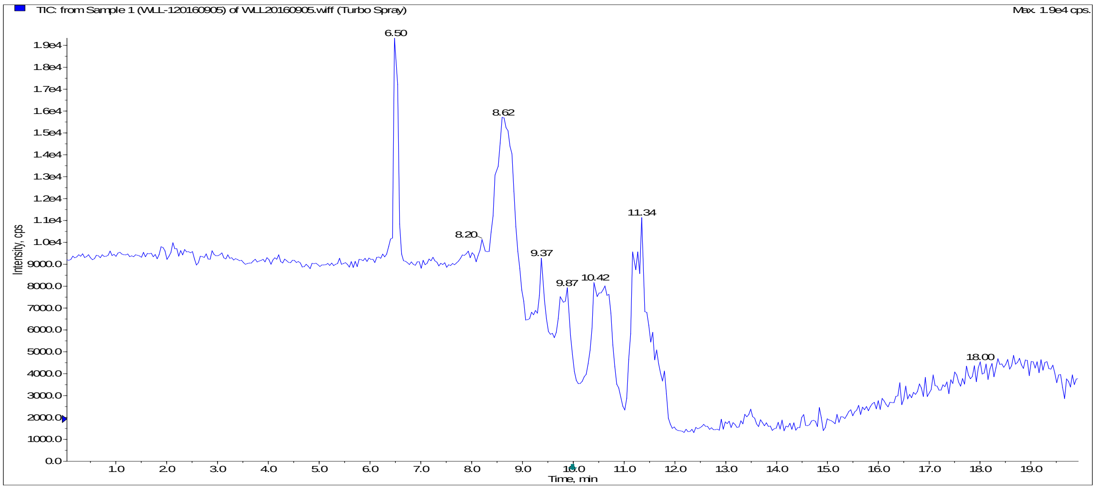

Figure S66. TIC of gossypol in $CHCl_3$ solution normal condition without protect from air and light storage condition in 3 days.

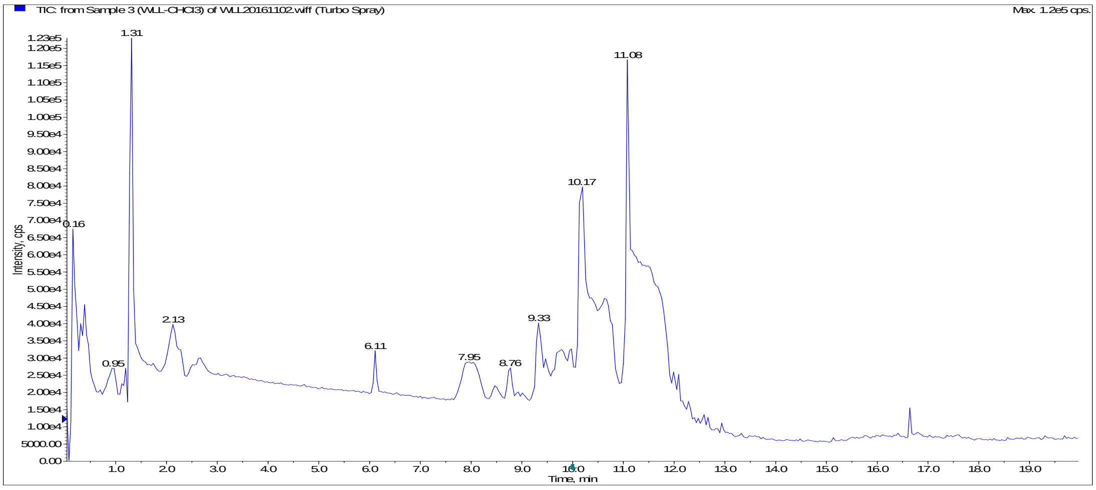

Figure S67. TIC of gossypol in $CHCl_3$ solution normal condition without protect from air and light storage condition in 5 days.

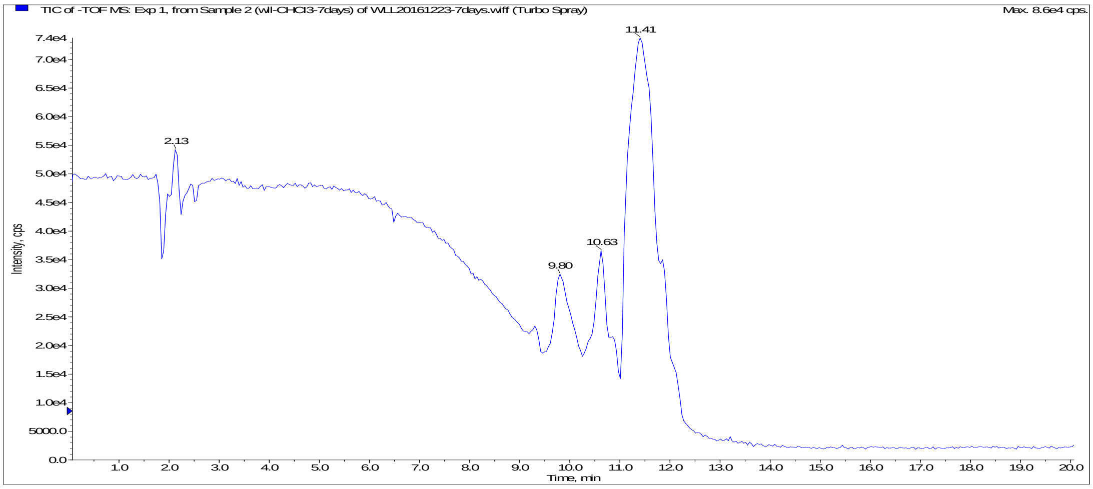

Figure S68. TIC of gossypol in $CHCl_3$ solution normal condition without protect from air and light storage condition in 7 days.

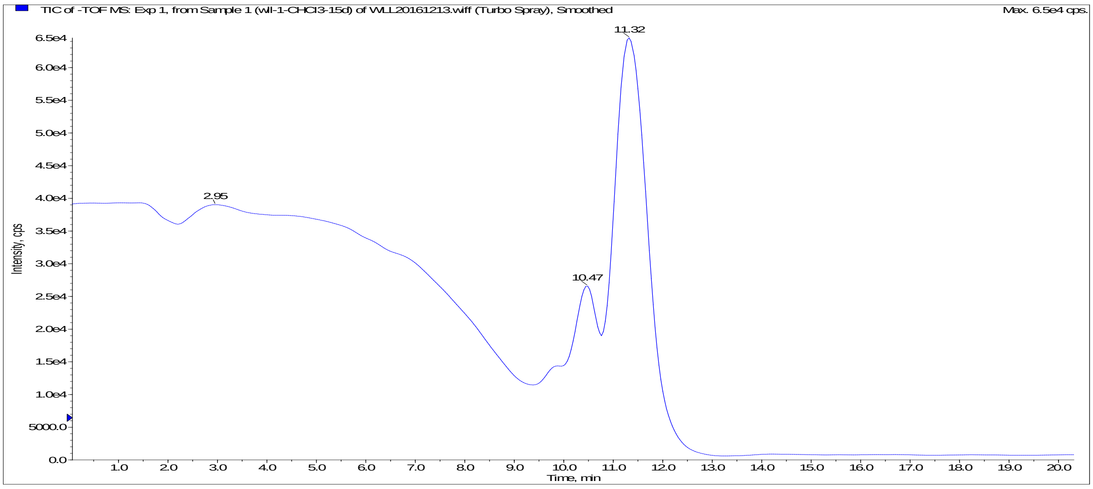

Figure S69. TIC of gossypol in $CHCl_3$ solution normal condition without protect from air and light storage condition in 15 days.

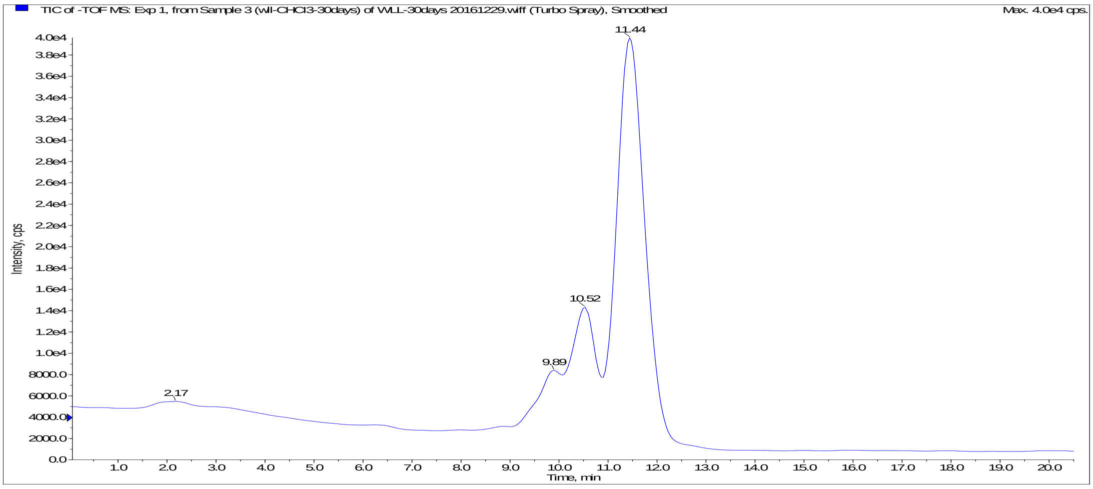

Figure S70. TIC of gossypol in $CHCl_3$ solution normal condition without protect from air and light storage condition in 30 days.

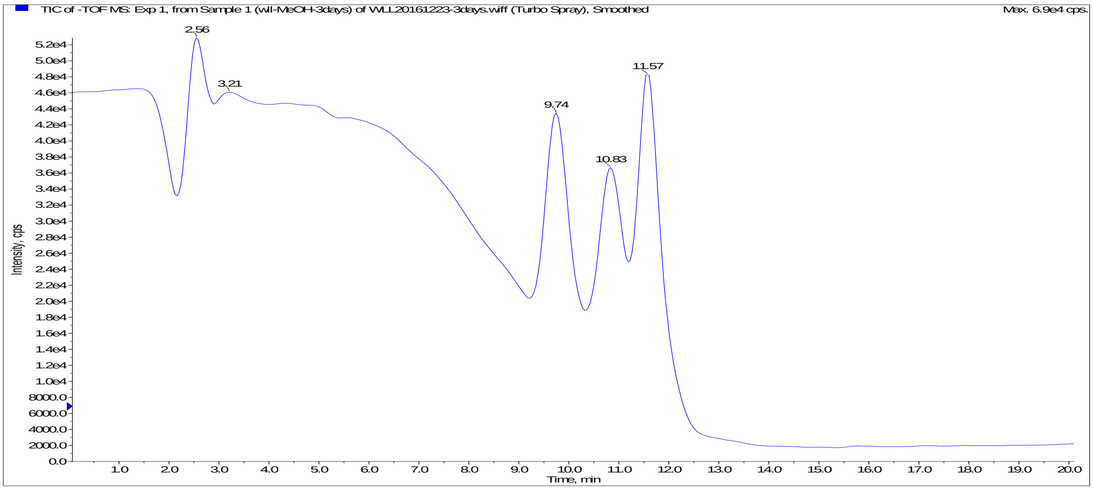

Figure S71. TIC of gossypol in CH_3OH solution normal condition without protect from air and light storage condition in 3 days.

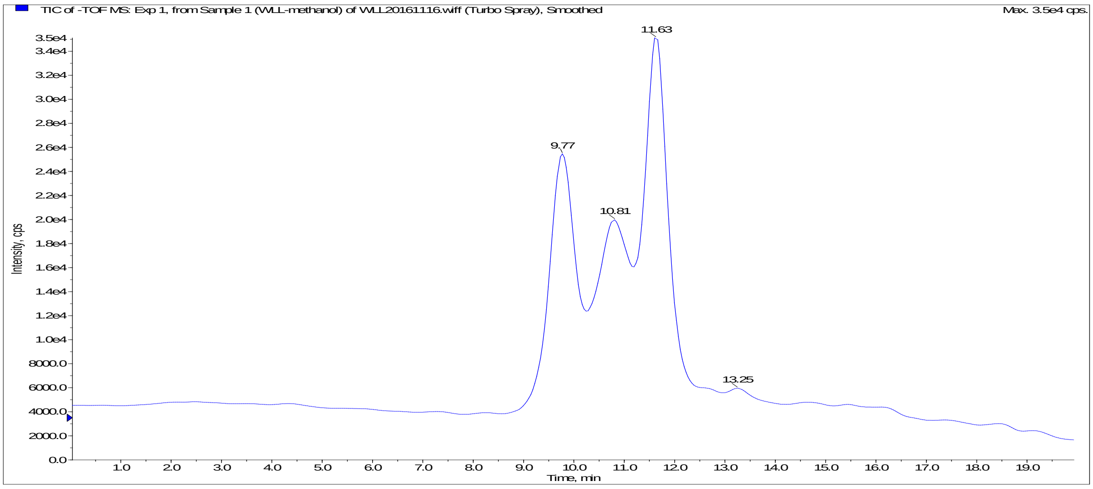

Figure S72. TIC of gossypol in CH_3OH solution normal condition without protect from air and light storage condition in 5 days.

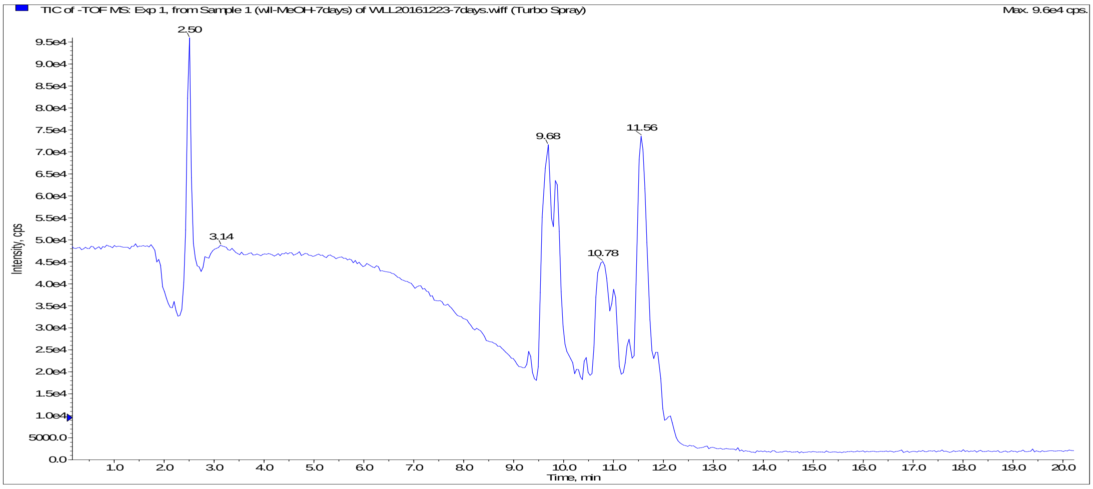

Figure S73. TIC of gossypol in CH_3OH solution normal condition without protect from air and light storage condition in 7 days.

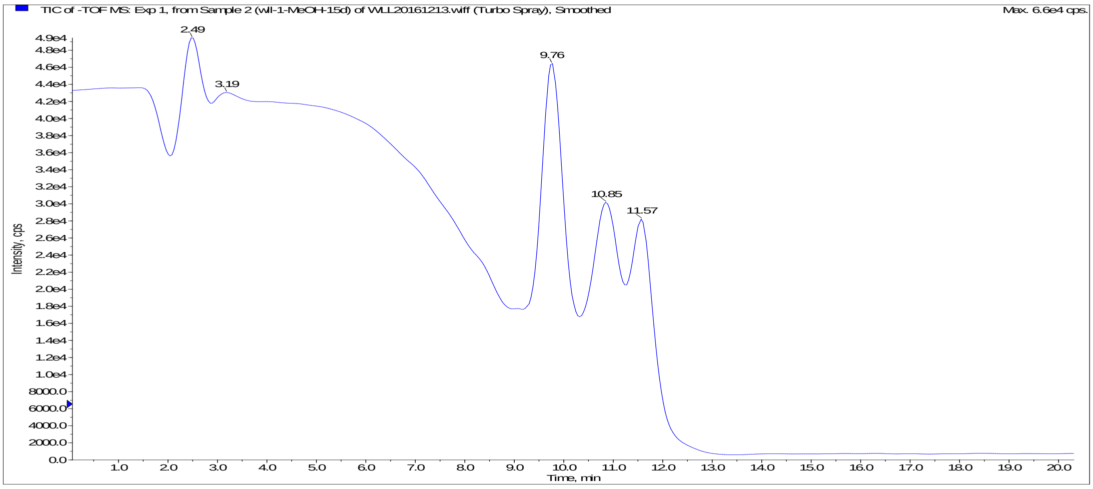

Figure S74. TIC of gossypol in CH_3OH solution normal condition without protect from air and light storage condition in 15 days.

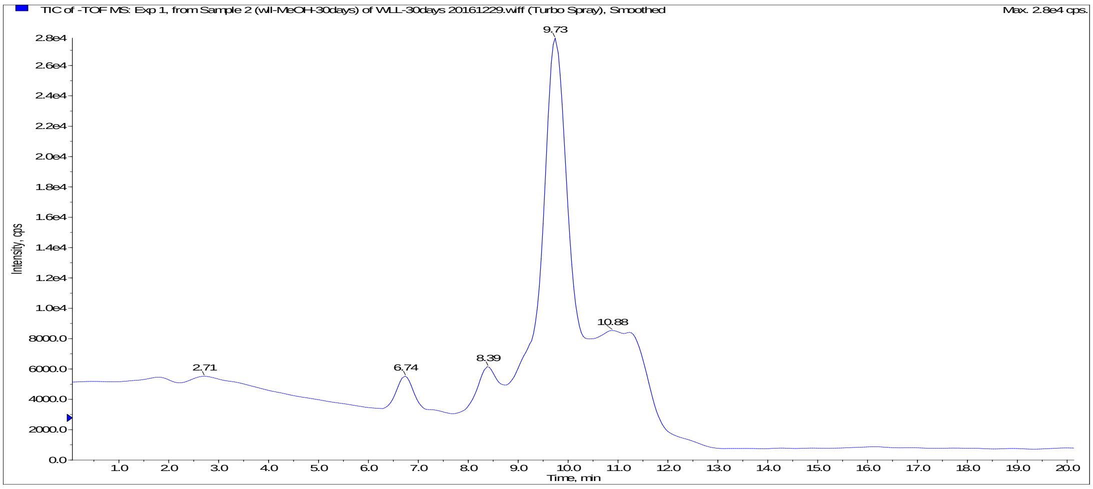

Figure S75. TIC of gossypol in CH_3OH solution normal condition without protect from air and light storage condition in 30 days.

Reference

1. Brzezinski, B.; Rozwadowski, J.;Marciniak, B.; Paszyc, S. Spectroscopic study of gossypol–lanthanide cation complexes in acetonitrile solution. *J. Mol. Struct*. **1997**,435, 275-279. doi: 10.1016/S0022-2860(97)00197-X.
